# TRIM63/IRF-8 axis promotes tumor progression and immunosuppression of melanoma with BRAF mutation

**DOI:** 10.1038/s41419-025-08216-5

**Published:** 2025-11-28

**Authors:** Fei Yi, Shuotong Liu, Yan Ma, Ye Li, Banchen Chen, Bo Yu

**Affiliations:** 1 Department of Dermatology, Peking University Shenzhen Hospital, Shenzhen, Guangdong China; 2 Shenzhen Key Laboratory for Translational Medicine of Dermatology, Biomedical Research Institute, Shenzhen Peking University-the Hong Kong University of Science and Technology Medical Center, Shenzhen, Guangdong China; 3 SDU-ANU Joint Science College, Shandong University, Weihai, Shandong China; 4 Department of Pathology, The First Affiliated Hospital of Soochow University, Suzhou, Jiangsu China; 5 Department of Oncology, The First Medical Center of Chinese PLA General Hospital, Beijing, China; 6 Oncology Laboratory, Chinese PLA General Hospital, Beijing, China

**Keywords:** Oncogenes, Ubiquitylation, Growth factor signalling

## Abstract

The E3 ligase TRIM63 demonstrates a robust correlation with melanoma malignancy, particularly in cases involving BRAF mutants. Meanwhile, BRAF mutants, such as V600E, represent a prominent mutation observed in melanoma patients, yet the underlying mechanism remains elusive. In this study, we demonstrate that TRIM63 exhibits overexpression in melanoma cells and exerts its full oncogenic potential upon activation of the MAPK signaling pathway. Mechanistically, BRAF mutation induces ERK1/2-mediated phosphorylation of TRIM63 at serine 69 (S69). TRIM63 S69 is localized in the RING domain, and its phosphorylation enhances TRIM63 binding with IRF-8. Subsequently, TRIM63 leads to the ubiquitination of IRF-8 at lysine 250 (K250). The degradation of IRF-8 ultimately contributes to tumor progression enhancement. Clinically, the presence of pS69 on TRIM63 is associated with tumor immunosuppression and poor prognosis among melanoma patients, highlighting its potential as a promising therapeutic target.

## Introduction

Melanoma, a mortal cutaneous tumor, presents clinical challenges due to its heterogeneity and varying genetic alterations [1–3]. BRAF mutations, particularly the BRAF V600E, are the most common molecular feature of melanoma, playing a pivotal role in the pathogenesis [4, 5]. BRAF mutations lead to the constitutive activation of the MAPK/ERK signaling pathway, thereby contributing to tumor growth and metastasis of melanocytes [6, 7]. While BRAF and MEK inhibitors have shown promise for patients with BRAF-mutated melanoma, melanoma’s propensity for developing resistance to therapies complicates treatment regimens, especially in advanced stages of the disease [8, 9]. Understanding the oncogenic role of BRAF mutations in melanoma is crucial for the development of effective therapeutic approaches and personalized medicine.

The E3 ubiquitin-protein ligase TRIM63, a member of the TRIM family of proteins, plays a crucial role in the pathophysiology of various diseases through its E3 ubiquitin ligase activity, regulating protein stability and sarcous signaling pathways, such as contractile proteins and telethonin [10–13]. While the function and regulatory mechanisms of TRIM63 have been extensively studied in skeletal muscle under different conditions, such as cancer, sepsis, diabetes, and renal failure [14–16], its involvement in tumors remains relatively understudied. Intriguingly, we observed significantly elevated expression levels of TRIM63 with strong clinical correlations in melanoma patients. This highlights an “uncultivated land” of TRIM63 in melanoma that warrants further investigation. Moreover, previous studies have reported that the regulation of TRIM63 primarily occurs at the transcriptional level through NF-κB, p63, and FoxO [17–19]. However, limited attention has been given to its post-translational modifications. A comprehensive understanding of the functional and mechanistic dynamics of TRIM63 may unveil potential therapeutic targets for melanoma treatment.

IRF-8 (Interferon Regulatory Factor 8) is a transcription factor that plays a crucial role in the regulation of immune responses and the development of various immune cells, including macrophages and dendritic cells [20–22]. In the context of tumors, IRF-8 has garnered attention for its dual role, acting both as a tumor suppressor and, in some cases, being associated with tumor promotion, depending on the cancer type and the microenvironment [23, 24]. In melanoma and breast cancer, IRF-8 has been shown to exhibit tumor-suppressive functions by enhancing anti-tumor immunity through the promotion of effective T cell responses for producing of pro-inflammatory cytokines and the differentiation and activation of myeloid-derived immune cells for recognizing and eliminating tumor cells [22, 25]. However, despite its presence in tumor cells, the role of IRF-8 in the tumorigenic properties of cancer cells remains largely unexplored. Our study reveals that IRF-8 exerts a tumor suppressive role in neoplastic cells, wherein the reinstatement of IRF-8 through TRIM63 abrogation elicits diverse cellular processes leading to tumor cell eradication. The intricate role of IRF-8 in cancer highlights its potential as a therapeutic target. A deeper understanding of IRF-8’s mechanisms can provide insights into developing novel strategies for cancer treatment and personalized medicine.

## Results

### TRIM63 is critical for melanoma progression

**Fig. 1:**
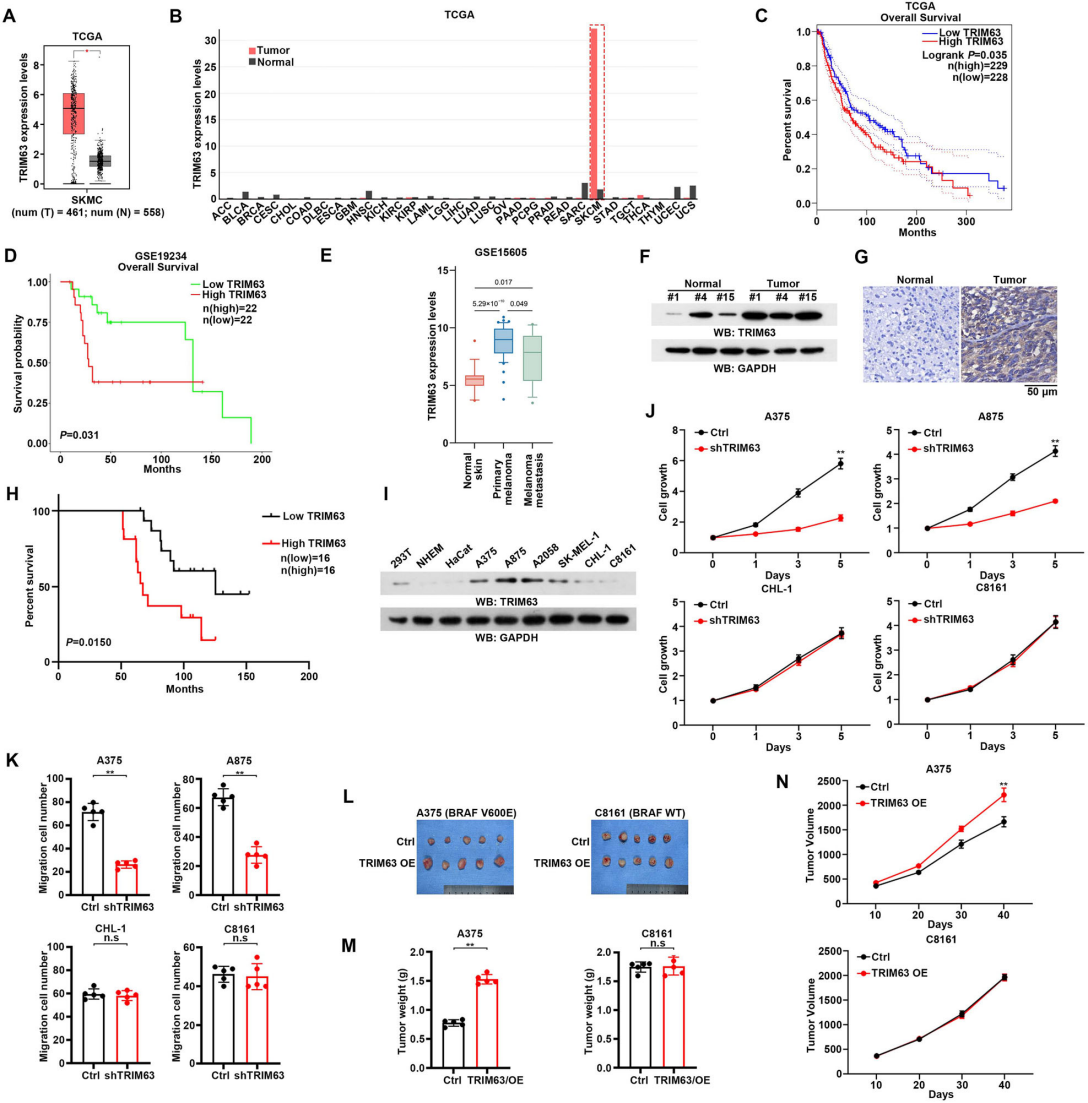
TRIM63 is critical for melanoma progression. **A** TRIM63 expression levels in SKCM patients in TCGA database. Box plots depict TRIM63 mRNA expression levels in tumor samples (*n* = 461) and adjacent normal skin samples (*n* = 558). Statistical significance was assessed using the *t*-test *P < 0.05). **B** TRIM63 expression levels across various cancer types in the TCGA database. Box plots depict TRIM63 mRNA expression levels in tumor samples and adjacent normal samples. The dotted box represents SKCM, where the changes are most significant. **C** Kaplan-Meier curves for overall survival (OS) of patients with SKCM stratified by TRIM63 mRNA expression levels. Patients from the TCGA SKCM cohort were divided into high (*n* = 229) and low (*n* = 228) TRIM63 expression groups using the median expression level as the cutoff. Statistical significance was determined by the log-rank test (*P* = 0.035). **D** Kaplan–Meier survival analysis of patients with melanoma based on TRIM63 expression levels in the GSE19234 database. Kaplan-Meier curves for overall survival (OS) of patients with SKCM stratified by TRIM63 mRNA expression levels. Patients from the GSE19234 cohort were divided into high (*n* = 16) and low (*n* = 16) TRIM63 expression groups using the median expression level as the cutoff. Statistical significance was determined by the log-rank test (*p* = 0.015). **E** TRIM63 expression levels in normal skin, primary melanoma, and metastatic melanoma in the GSE15605 database. Box plots depict TRIM63 mRNA expression levels in normal skin samples (*n* = 17), primary melanoma samples (*n* = 46), and metastatic melanoma (*n* = 12). Statistical significance was assessed using the t-test *p < 0.05). **F** Immunoblotting analyses of TRIM63 expression levels in normal skin and melanoma tissues. The number represents the patient’s identification. **G** Immunohistochemistry analyses of TRIM63 expression levels in normal skin and melanoma tissues. **H** Kaplan–Meier survival analysis of 32 collected clinical melanoma samples based on TRIM63 IHC score, High TRIM63 (*n* = 16), Low TRIM63 (*n* = 16). **I** Immunoblotting analyses of TRIM63 expression levels in the indicated cells. NHEM (normal human epidermal melanocytes), HaCaT (human immortalized epidermal cells), melanoma cell (BRAF V600E: A375, A875, A2058, SK-MEL-1, BRAF WT: CHL-1, C8161). **J** The cell growth of A375, A875, CHL-1, and C8161 cells with or without TRIM63 shRNA was measured by CCK8. Data were presented as mean ± SD from 5 independent experiments. **P < 0.001. **K** Depletion of TRIM63 in A375, A875, CHL-1, and C8161 cells, and measured the migration ability of these cells by migration assay. Data were presented as mean ± SD from 3 independent experiments. **P < 0.001, no significance. **L**–**N** A375 and C8161 cells (5 × 10^5^) with or without TRIM63 overexpression were subcutaneously injected into athymic nude mice (*n* = 5 per group). Images of tumors in mice were shown (**L**). Bar graph showing meaning tumor weight ± standard deviation (SD), **P < 0.001, n.s, no significance (**M**). The growth of tumors was counted by tumor volume, **P < 0.001, n.s, no significance (**N**).

The ubiquitin system is complex and critical, which is often disordered in tumor progression. We analyzed TCGA database and observed that TRIM63, a E3 ligase, was significant higher in Skin Cutaneous Melanoma (SKCM), compared to that in normal skin tissues (Fig. 1A). Furthermore, the basal levels of TRIM63 were specifically highly expressed in SKCM across all the tumor types (Fig. 1B). Those data prompted that the TRIM63 may play a vital role in the progression of melanoma. Further analysis indicated that high expression of TRIM63 represented a worse prognosis of melanoma patients (Fig. 1C). Similar outcomes were also observed from other datasets (GSE19234, GSE15605), which suggested the generality of high TRIM63 expression in melanoma (Fig. 1D, E). Moreover, a total of 65 melanoma samples were collected from clinical settings. The protein levels of TRIM63 from our clinical samples were also higher in melanoma than in normal skin tissues (Fig. 1F, G). The clinical information also indicates that patients with high TRIM63 expression levels showed worse survival duration than those with low TRIM63 expression levels (Fig. 1H). Furthermore, we determined that the TRIM63 expression in different cell lines, which showed higher levels of TRIM63 in melanoma cells compared to the normal human epidermal melanocytes, meanwhile, the melanoma cell lines with BRAF V600E showed higher ERK activation compared with those without BRAF mutation (Fig. 1I and Supplementary Fig. 1A).

To investigate the impact of TRIM63, we conducted interference experiments in various cell lines (Supplementary Fig. 1B–E). Notably, after TRIM63 depletion, the oncogenic effect of TRIM63 exhibited significant alterations exclusively in melanoma cells harboring BRAF mutations, while no such changes were observed in cells without BRAF mutation (Fig. 1J). Consistent results were also obtained from migration assays (Fig. 1K and Supplementary Fig. 1F). Furthermore, in vivo studies demonstrated that TRIM63 overexpression exerted effects solely on melanoma cells with BRAF mutations rather than those with wild-type BRAF (Fig. 1L–N and Supplementary Fig. 1G). In addition, to confirm the TRIM63 carcinogenic effect in the cells expressing low TRIM63 levels, CHL-1 cells, which exhibit relatively low endogenous TRIM63 expression, were transfected to overexpress TRIM63 with or without EGF treatment. The results demonstrated that, in the absence of EGF, overexpression of TRIM63 had a moderate effect. In contrast, a significant effect was observed when both TRIM63 overexpression and EGF treatment were applied (Supplementary Fig. 1H). Collectively, these findings strongly support the notion that TRIM63 functions as an oncogene and its protein levels are not the most critical factor, but rather the activation states of TRIM63 play the most important role.

### ERK1/2 protein interacts with and phosphorylates TRIM63 at residue S69

**Fig. 2:**
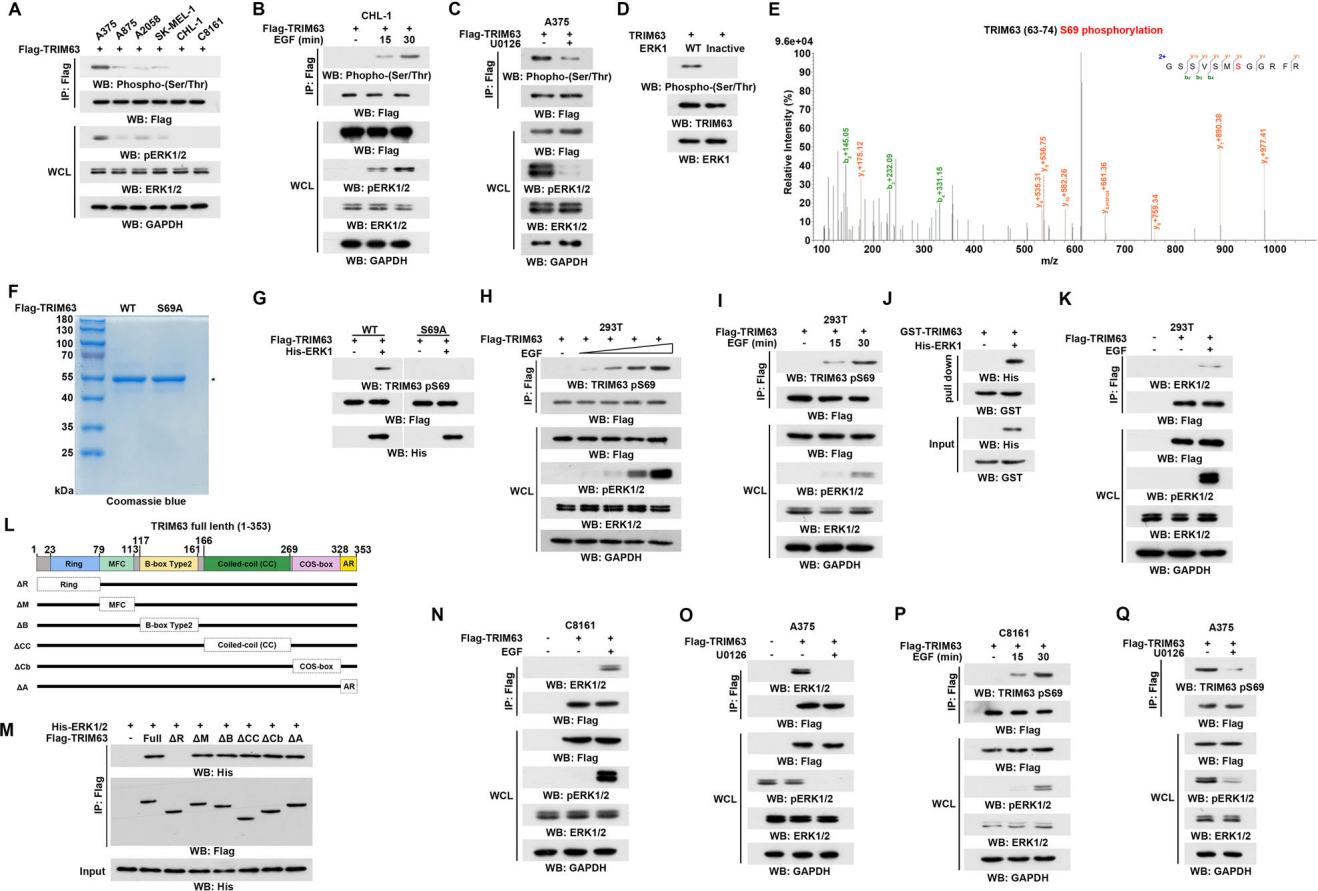
ERK1/2 protein interacts with and phosphorylates TRIM63 at residue S69. **A** Indicated cells transfected with Flag-TRIM63 (2 μg) were lysed and performed by immunoprecipitation with Flag antibody. Then, immunoblotting was performed with indicated antibodies. **B** CHL-1 cells transfected with Flag-TRIM63 (2 μg) were treated with EGF (100 ng/ml) for 15- and 30-min. Immunoprecipitation with Flag antibody and immunoblotting were performed with indicated antibodies. **C** A375 cells were transfected with Flag-TRIM63 (2 μg) and treated with U1026 (20 μM) for 30 min. Immunoprecipitation was performed by incubating the Flag antibodies (10 mg) with 1 mg of cell lysates at 4 °C overnight, followed by incubation of protein A/G-agarose beads for 3 hours. The beads were then washed with lysis buffer 4 times. Immunoblotting analyses were performed with indicated antibodies. **D** In vitro kinase assays were performed by mixing bacterially purified TRIM63 (2 μg) with active ERK1 (1 μg) or inactive ERK1(1 μg). Immunoblotting analyses were performed. **E** Flag-TRIM63 expressed in A375 cells was immunoprecipitated and analyzed by mass spectrometry. **F** Coomassie staining of purified Flag-TRIM63 WT and Flag-TRIM63 S69A mutant from 293 T cells. **G** Validation of antibody against TRIM63 pS69. In vitro kinase assays were performed by mixing bacterially purified Flag-TRIM63 WT and Flag-TRIM63 S69A mutant with or without active ERK1. Immunoblotting analyses were performed with indicated antibodies. **H** 293 T cells were transfected with Flag-TRIM63 and treated with dose escalation EGF gradually (0, 10, 20, 50, 100 ng/ml). Immunoprecipitation was performed by incubating the Flag antibodies (10 mg) with 1 mg of cell lysates at 4 °C overnight, followed by incubation of protein A/G-agarose beads for 3 hours. The beads were then washed with lysis buffer 4 times. Immunoblotting analyses were performed with indicated antibodies. **I** 293 T cells were transfected with Flag-TRIM63 and treated with EGF for 15- and 30-min. Immunoprecipitation was performed by incubating the Flag antibodies (10 mg) with 1 mg of cell lysates at 4 °C overnight, followed by incubation of protein A/G-agarose beads for 3 hours. The beads were then washed with lysis buffer 4 times. Immunoblotting analyses were performed with the indicated antibodies. **J** Pull down analyses of GST-TRIM63 and His-ERK1. Immunoblotting analyses were performed with indicated antibodies. **K** 293 T cells were transfected with Flag-TRIM63 and treated with EGF (100 ng/ml) for 30 min. Immunoprecipitation was performed by incubating the Flag antibodies (10 mg) with 1 mg of cell lysates at 4 °C overnight, followed by incubation of protein A/G-agarose beads for 3 hours. The beads were then washed with lysis buffer 4 times. Immunoblotting analyses were performed with indicated antibodies. **L** Schematic representation of full-length and truncated mutation of TRIM63. **M** The purified His-ERK1 was incubated with purified full-length or truncated TRIM63 for 4 hours in vitro. Pull-down and immunoblotting analyses with indicated antibodies were performed. **N** C8161 cells were transfected with Flag-TRIM63 and treated with EGF (100 ng/ml) for 30 min. Immunoprecipitation was performed by incubating the Flag antibodies (10 mg) with 1 mg of cell lysates at 4 °C overnight, followed by incubation of protein A/G-agarose beads for 3 hours. The beads were then washed with lysis buffer 4 times. Immunoblotting analyses were performed with indicated antibodies. **O** A375 cells were transfected with Flag-TRIM63 and treated with U1026 (20 μM) for 30 min. Immunoprecipitation was performed by incubating the Flag antibodies (10 mg) with 1 mg of cell lysates at 4 °C overnight, followed by incubation of protein A/G-agarose beads for 3 hours. The beads were then washed with lysis buffer 4 times. Immunoblotting analyses were performed with indicated antibodies. **P** C8161 cells were transfected with Flag-TRIM63 and treated with EGF (100 ng/ml) for 15 and 30 min. Immunoprecipitation was performed by incubating the Flag antibodies (10 mg) with 1 mg of cell lysates at 4 °C overnight, followed by incubation of protein A/G-agarose beads for 3 hours. The beads were then washed with lysis buffer 4 times. Immunoblotting analyses were performed with indicated antibodies. **Q** A375 cells were transfected with Flag-TRIM63 and treated with U1026 (20 μM) for 30 min. Immunoprecipitation was performed by incubating the Flag antibodies (10 mg) with 1 mg of cell lysates at 4 °C overnight, followed by incubation of protein A/G-agarose beads for 3 hours. The beads were then washed with lysis buffer 4 times. Immunoblotting analyses were performed with indicated antibodies.

BRAF mutations induce MAPK signaling activation and subsequent ERK1/2-mediated phosphorylation. Considering TRIM63-dysfunction in melanoma cells carrying BRAF mutant, we speculated that TRIM63 may be associated with BRAF mutation. As anticipated, we observed that TRIM63 is significantly phosphorylated in melanoma cells with BRAF V600E compared to those with wild-type BRAF, while the expression levels remained comparable (Fig. 2A). Further, ERK1/2 inhibition by U0126 or activation by EGF showed that MAPK activation may be mainly in charge of TRIM63 phosphorylation (Fig. 2B, C). To determine whether TRIM63 is a substrate of ERK, we performed in vitro protein kinase analyses and showed that purified active ERK1 phosphorylated bacterially purified TRIM63 (Fig. 2D). Further, to investigate the phosphorylated site of TRIM63, we overexpressed Flag-TRIM63 in A375 cells, followed by immunoprecipitation and mass spectrometry (MS) analyses. MALDI-TOF/TOF MS suggested that ERK1/2 phosphorylated TRIM63 at serine (S) 69 (Fig. 2E). To specifically detect TRIM63 phosphorylation, the antibody recognizing TRIM63 pS69 were obtained. For testing antibody specificity, TRIM63 mutation of S to alanine (A) is established to abrogate the modification of phosphorylation. Then, TRIM63 WT and S69A were purified bacterially (Fig. 2F). The signal of TRIM63 pS69 was observed in the recombination proteins of TRIM63 WT but not in the mutant protein (Fig. 2G). Furthermore, purification of TRIM63 from HEK293T cells demonstrated that the phosphorylation levels of TRIM63 pS69 increased in a dose- and time-dependent manner following EGF treatment (Fig. 2H, I).

To investigate the interaction between TRIM63 and ERK1, we primarily conducted pull-down assays, showing that TRIM63 could bind with ERK1 (Fig. 2J). Subsequently, in vivo assays also demonstrated the interaction between TRIM63 and ERK1, which exhibited a response to EGF treatment (Fig. 2K). To further detect the binding domain of TRIM63 with ERK1/2, we established the truncated fragments of TRIM63, respectively (Fig. 2L). Pull-down assays showed that the deletion of RING domain presented the most significant reduction of interaction (Fig. 2M). Subsequently, the interaction between TRIM63 and activated ERK1/2 was investigated in melanoma cells. The activation of the MAPK pathway led to the binding of ERK1/2 with TRIM63, resulting in TRIM63 phosphorylation specifically within BRAF wild-type cells (Fig. 2N, P). Consistently, treatment with the MAPK inhibitor U0126 abolished the interaction between ERK and TRIM63, and inhibited TRIM63 phosphorylation in melanoma cells carrying BRAF V600E (Fig. 2O, Q). Collectively, the data indicated that TRIM63 interacts with ERK1/2 and undergoes phosphorylation by ERK1/2 within melanoma cells harboring BRAF mutations.

### ERK1/2-induced TRIM63 pS69 enhances its oncogenic efficacy

**Fig. 3:**
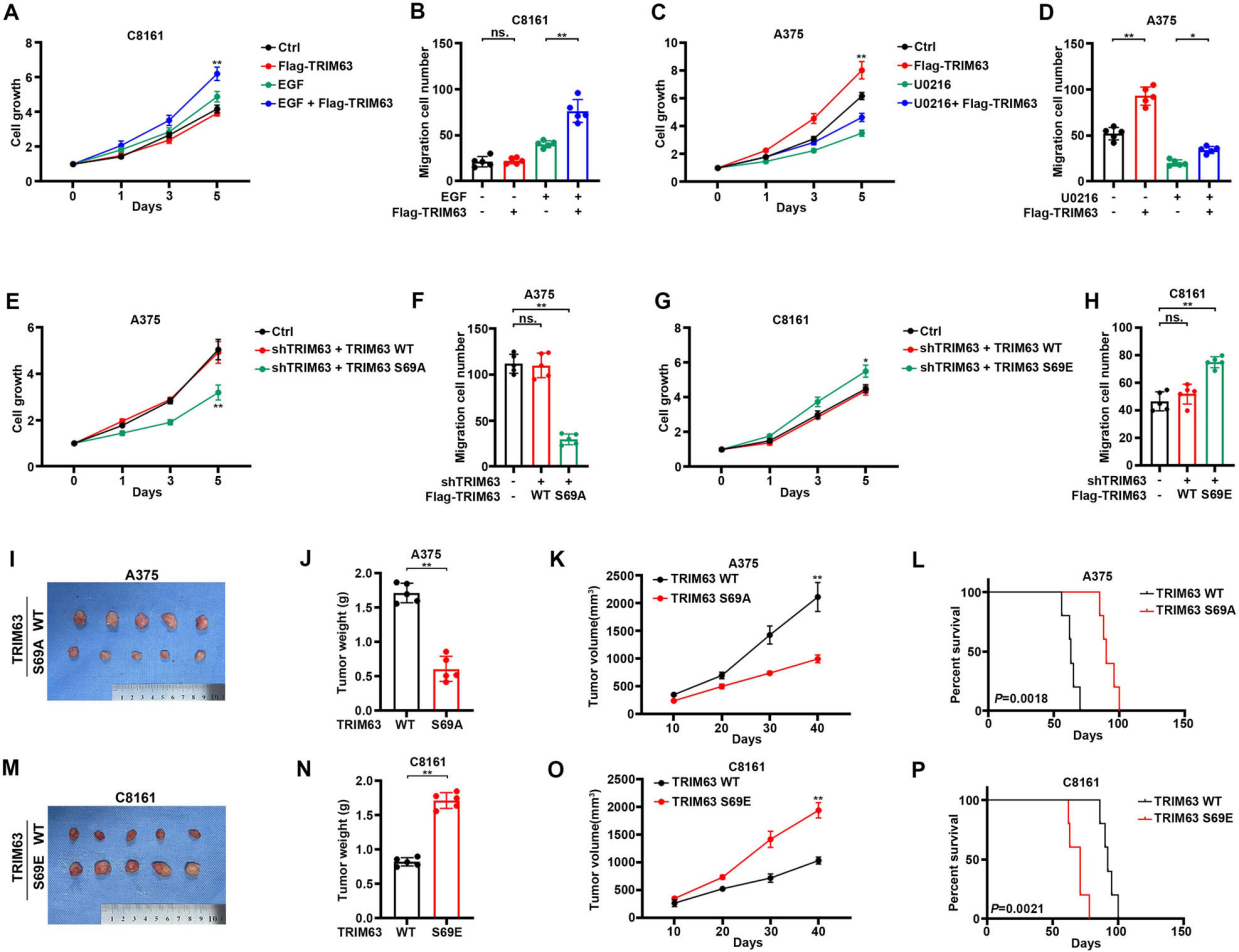
ERK1/2-induced TRIM63 pS69 enhances its oncogenic efficacy. **A**, **B** C8161 cells were transfected with or without Flag-TRIM63 and treated with or without EGF (100 ng/ml). **A** The growth of C8161 cells measured by CCK8-assay. Cell growth was expressed as optical density (OD) values. Values were standardized by 0 day. Data were presented as mean ± SD from 5 independent experiments. **P < 0.001. **B** The migration ability of C8161 cells was measured by migration assay. Histograms represent the analysis of the migration assay. Data were presented as mean ± SD from 3 independent experiments. **P < 0.001, no significance. **C**, **D** A375 cells transfected with or without Flag-TRIM63 and treated with or without U0126 (20 μM). **C** The growth of A375 cells measured by CCK8-assay. Cell growth was expressed as optical density (OD) values. Values were standardized by 0 day. Data were presented as mean ± SD from 5 independent experiments. **P < 0.001. **D** The migration ability of A375 cells was measured by migration assay. Histograms represent the analysis of the migration assay. **P < 0.001, *P < 0.05. E-F. A375 cells were transfected with or without TRIM63 shRNA and then transfected with Flag-TRIM63 WT or S69A mutant. **E** The growth of A375 cells measured by CCK8-assay. Cell growth was expressed as optical density (OD) values. Values were standardized by 0 day. Data were presented as mean ± SD from 5 independent experiments. **P < 0.001. **F** The migration ability of A375 cells was measured by migration assay. Histograms represent the analysis of the migration assay. **P < 0.001, no significance. **G**, **H** C8161 cells were transfected with or without TRIM63 shRNA and then transfected with Flag-TRIM63 WT or S69E mutant. **G** The growth of C8161 cells measured by CCK8-assay. Cell growth was expressed as optical density (OD) values. Values were standardized by 0 day. Data were presented as mean ± SD from 5 independent experiments. *P < 0.05. **H** The migration ability of C8161 cells was measured by migration assay. Histograms represent the analysis of the migration assay. **P < 0.001, no significance. I-L. 5 × 10^5^ of A375 cells expressing TRIM63 WT or S69A were subcutaneously injected into athymic nude mice (*n* = 5 per group). **I** Image of a Tumor in mice was shown. **J** Bar graph showing mean tumor weight ± standard deviation (SD), **P < 0.001. **K** The growth of tumors was counted by tumor volume. Statistical significance at 40 days after tumor vaccination. **P < 0.001. **L** Kaplan–Meier survival curve of mice bearing subcutaneous tumors, *P* = 0.0018. M-P. 5 × 10^5^ of C8161 cells expressing TRIM63 WT or S69E were subcutaneously injected into athymic nude mice (*n* = 5 per group). **M** Image of Tumor in mice was shown. **N** Bar graph showing mean tumor weight ± standard deviation (SD), **P < 0.001. **O** The growth of tumors was counted by tumor volume. Statistical significance at 40 days after tumor vaccination. **P < 0.001. **P** Kaplan–Meier survival curve of mice bearing subcutaneous tumors, *P* = 0.0021.

To investigate the functional role of ERK1/2-induced TRIM63 phosphorylation, we subjected C8161 (BRAF WT) to EGF stimulation while concurrently overexpressing TRIM63 or not. Dramatically, cell proliferation and migration are significantly increased in the group overexpressing TRIM63 (Fig. 3A, B, Supplementary Fig. 1A). Consistently, repression of ERK activity by U0126 treatment in A375 cells (BRAF V600E) abrogates the impact of TRIM63 overexpression in proliferation and migration (Fig. 3C, D, Supplementary Fig. 1B). The data prompted that for TRIM63 to exert its carcinogenic effect, both MAPK signal activation and TRIM63 overexpression are essential. To further confirm the effect of TRIM63 pS69, we knocked in TRIM63 S69A in A375 cells to abolish S69 phosphorylation (Supplementary Fig. 1C). Meanwhile, C8161 (BRAF WT) cells were knocked in TRIM63 S69E to mimic S69 phosphorylation (Supplementary Fig. 1D). Then, TRIM63 S69A resulted in impaired ability of proliferation and migration of A375 cells, compared with TRIM63 WT (Fig. 3E, F). On the contrary, TRIM63 S69E resulted in enhanced proliferation and migration of C8161 cells, compared with TRIM63 WT (Fig. 3G, H).

To test in vivo effect of TRIM63 pS69, tumor xenograft models were established. A375 cells with or without TRIM63 S69A were subcutaneously implanted nude mice. And TRIM63 S69A knock-in resulted in a significant reduction in tumor weight (Fig. 3I, J), growth rate (Fig. 3K), and improved survival duration (Fig. 3L) compared to the group with TRIM63 WT. Similarly, the mice bearing C8161 with TRIM63 S69E showed a significant increase in tumor weight (Fig. 3M, N), growth rate (Fig. 3O), and worse survival duration (Fig. 3P) compared to the group with TRIM63 WT. Conclusively, we have demonstrated that the potential carcinogenic effect of TRIM63 is contingent upon ERK1/2-induced phosphorylation of TRIM63 at S69.

### ERK1/2-induced TRIM63 pS69 impairs IRF-8 protein stability

**Fig. 4:**
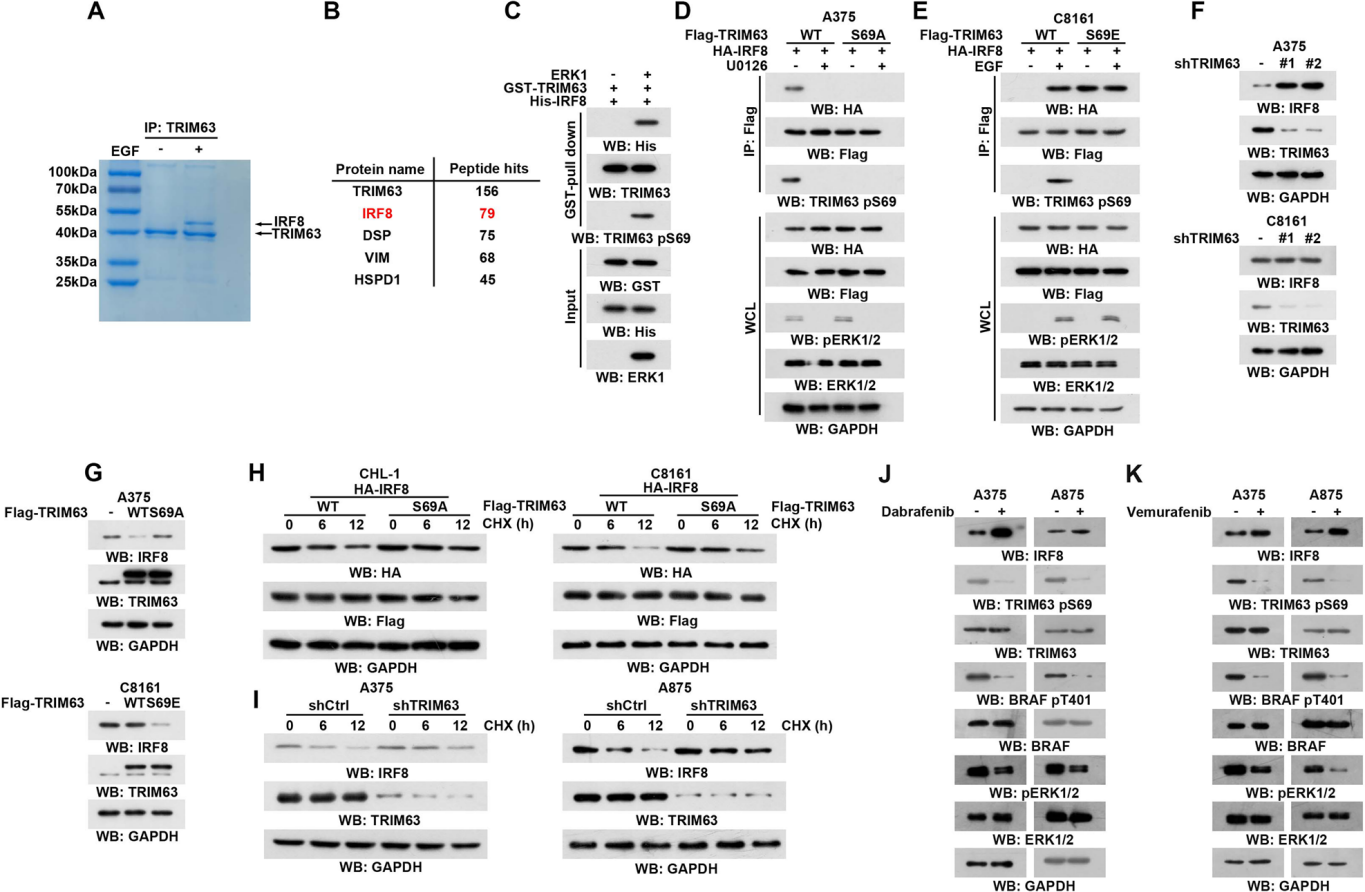
ERK1/2-induced TRIM63 pS69 impairs IRF-8 protein stability. **A**, **B** 293 T cells were treated with or without EGF (100 ng/mL) for 30 min. **A** Immunoprecipitation of TRIM63 binding protein with an anti-TRIM63 antibody was eluted and separated using sodium dodecyl sulfate (SDS)-polyacrylamide gel electrophoresis (PAGE) and stained with Coomassie brilliant blue. **B** The indicated protein band was excised for mass spectrometric analysis and identified as IRF8. **C** GST pull-down analyses of GST-TRIM63 and His-IRF8 treatment with or without ERK1. Immunoblotting analyses were performed with the indicated antibodies. **D** A375 cells treated with or without U0126 (20 μM) were transfected with HA-IRF8 and Flag-TRIM63 WT or S69A mutant. Immunoprecipitation was performed by incubating the Flag antibodies (10 mg) with 1 mg of cell lysates at 4 °C overnight, followed by incubation of protein A/G-agarose beads for 3 hours. The beads were then washed with lysis buffer 4 times. Immunoblotting analyses were performed with the indicated antibodies. **E** C8161 cells treated with or without EGF (100 ng/ml) were transfected with HA-IRF8 and Flag-TRIM63 WT or S69E mutant. Immunoprecipitation was performed by incubating the Flag antibodies (10 mg) with 1 mg of cell lysates at 4 °C overnight, followed by incubation of protein A/G-agarose beads for 3 hours. The beads were then washed with lysis buffer 4 times. Immunoblotting analyses were performed with the indicated antibodies. **F** A375 and C8161 cells were transfected with negative control shRNA or shTRIM63 RNA #1 and #2 for 72 h. Immunoblotting analyses were performed with the indicated antibodies. **G** A375 cells were transfected with Flag-TRIM63 WT or S69A mutant (2 μg). C8161 cells were transfected with Flag-TRIM63 WT or S69E mutant (2 μg). Immunoblotting analyses were performed with the indicated antibodies. **H** CHL-1 and C8161 cells treated with EGF were transfected with HA-IRF8 and Flag-TRIM63 WT or S69A mutant and incubated with cycloheximide (CHX) (50 μM) for the indicated times. Immunoblotting analyses were performed with the indicated antibodies. **I** A375 and A875 cells expressing HA-IRF8 were transfected with negative control shRNA or TRIM63 shRNA and incubated with cycloheximide (CHX) (50 μM) for the indicated times. Immunoblotting analyses were performed with the indicated antibodies. **J** A375 and A875 cells were treated with BRAF inhibitor Dabrafenib (1 μM) for 1 h and lysed. Immunoblotting analyses were performed with the indicated antibodies. **K** A375 and A875 cells were treated with BRAF inhibitor Vemurafenib (50 nM) for 1 h and lysed. Immunoblotting analyses were performed with the indicated antibodies.

To elucidate the underlying oncogenic mechanism of ERK1/2-induced TRIM63 phosphorylation, we conducted a protein binding profile analysis of TRIM63 in HEK293T cells with or without EGF stimulation. Notably, upon EGF treatment, an observed band at approximately 48 kDa was detected (Fig. 4A). LC/MS analysis showed that this specific band was IRF-8 (Fig. 4B). We further validated the ERK-mediated interaction in vitro using GST-tagged TRIM63 and His-tagged IRF-8 (Fig. 4C). In vivo, ERK1/2 induces TRIM63 phosphorylation and its subsequent binding with IRF-8, a process that is disrupted by the TRIM63 S69A mutant (Fig. 4D). Conversely, the TRIM63 S69E mutant mimics phosphorylation-mediated IRF-8 binding independent of ERK1/2 activation (Fig. 4E). Thus, the significance of ERK1/2-induced TRIM63 pS69 in IRF-8 binding is pivotal based on the obtained data.

Subsequently, to investigate the impact of TRIM63 on IRF-8, we conducted TRIM63 depletion, which resulted in an increase in IRF-8 protein levels specifically in melanoma cells harboring BRAF V600E mutation, while no such effect was observed in cells with wild-type BRAF (Fig. 4F). Consistently, TRIM63 overexpression led to a reduction in IRF-8 levels specifically in BRAF mutated cells, while no such effect was observed in the wild-type cells. (Fig. 4G). To investigate the mechanism, we primarily detect that protein stability of IRF-8 in BRAF V600E melanoma cells, indicating that cycloheximide treatment significantly decreased IRF-8 protein levels, partially restored by TRIM63 deletion (Fig. 4H). Further, TRIM63 S69A, the non-phosphorylated mutant, also shows similar effects as the deletion (Fig. 4I). Dabrafenib and Vemurafenib function as inhibitors of B-Raf, targeting cancers associated with mutations in the BRAF gene. To investigate whether inhibition of ERK activation prevents TRIM63 phosphorylation and IRF-8 degradation, we found that treatment of BRAF-mutant tumor cells with Dabrafenib and Vemurafenib suppressed TRIM63 phosphorylation at S69 while concurrently increasing IRF-8 protein levels (Fig. 4J, K). Together, these data suggest that TRIM63 is an IRF-8-interacting protein responsible for the regulation of IRF-8 proteostasis.

### TRIM63 induces the K48-linked polyubiquitination of IRF-8

**Fig. 5:**
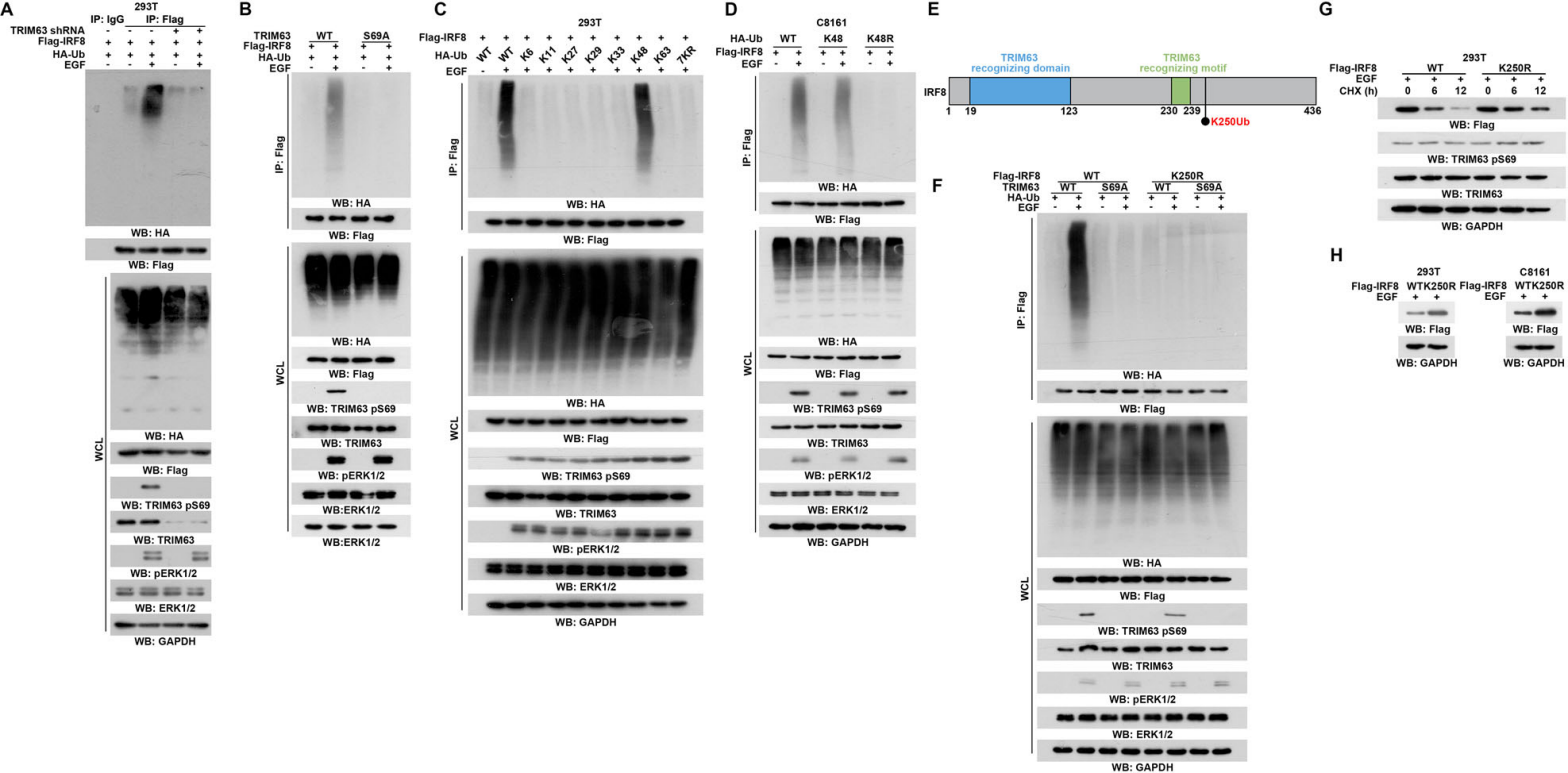
TRIM63 induces the K48-linked polyubiquitination of IRF-8. **A** 293 T cells depletion with or without TRIM63 were transfected with Flag-IRF8 and HA-ubiquitin together treated with EGF (100 ng/ml) or not, and then treated with MG132 (20 µM) for 6 h. Immunoprecipitation and immunoblotting analyses were performed with the indicated antibodies. **B** 293 T cells expressing of TRIM63 WT or S69A mutant were transfected with Flag-IRF8 and HA-ubiquitin together treated with EGF (100 ng/ml) or not, and then treated with MG132 (20 µM) for 6 h. Immunoprecipitation and immunoblotting analyses were performed with the indicated antibodies. **C** 293 T cells were transfected with Flag-IRF8 and indicated mutant HA-ubiquitin, together treated with EGF (100 ng/ml) or not and then treated with MG132 (20 µM) for 6 h. Immunoprecipitation and immunoblotting analyses were performed with the indicated antibodies. **D** C8161 cells were transfected with Flag-IRF8 and HA-ubiquitin K48 or K48R together treated with EGF (100 ng/ml) or not and then treated with MG132 (20 µM) for 6 h. Immunoprecipitation and immunoblotting analyses were performed with the indicated antibodies. “K48” indicates that lysine 48 is not mutated, while all other lysine residues are mutated, whereas “K48R” indicates that only lysine 48 is mutated. **E** Schematic representation of the IRF8 recognized domain by TRIM63. **F** 293 T cells expressing TRIM63 WT or S69A mutant were transfected with Flag-IRF8 WT or K250R and HA-ubiquitin together treated with EGF (100 ng/ml) or not, and then treated with MG132 (20 µM) for 6 h. Immunoprecipitation and immunoblotting analyses were performed with the indicated antibodies. **G** 293 T cells treated with EGF (100 ng/ml) were transfected with Flag-IRF8 WT or K250R mutant and incubated with cycloheximide (CHX) (50 μM) for the indicated times. Immunoblotting analyses were performed with the indicated antibodies. **H** 293 T and C8161 cells treated with EGF (100 ng/ml) were transfected with Flag-IRF8 WT or K250R mutant. Immunoblotting analyses were performed with the indicated antibodies.

Protein stability is mainly controlled by the ubiquitination system. Considering that TRIM63 is an E3 ligase, we wondered whether TRIM63 mediates the ubiquitination of IRF-8 to regulate its protein stability. To investigate this, we overexpressed Flag-IRF8 and TRIM63 shRNA along with HA-Ub in HEK293T cells and performed Co-immunoprecipitation assays. The findings demonstrated that TRIM63 ubiquitinates IRF-8 in response to ERK1/2 activation (Fig. 5A). Additionally, IRF-8 ubiquitination is dependent on TRIM63 S69 phosphorylation (Fig. 5B). Polyubiquitination can occur through seven different Lys residues on ubiquitin (K6, K11, K27, K29, K33, K48, and K63) [26, 27]. We thus used a panel of ubiquitin mutants in which only one Lys residue was retained, and we found that TRIM63 enhanced K48-linked polyubiquitination of IRF-8 in HEK293T (Fig. 5C). What is consistent is that reproducible results were also observed and validated in melanoma cells (Fig. 5D). Subsequently, we employed a bioinformatic tool (http://ubibrowser.bio-it.cn/ubibrowser_v3/) to predict the potential ubiquitinated residue of IRF-8 [28], revealing that lysine 250 (K250) is the identified site for ubiquitination (Fig. 5E). Then, we generated the ubiquitination-resistant mutant IRF-8 K250R to experimentally validate the predicted outcome. Consistent with expectations, IRF-8 K250R abrogated ERK1/2-induced ubiquitination, independent of TRIM63 phosphorylation (Fig. 5F). Furthermore, IRF-8 K250R also enhanced its stability, compared to IRF-8 WT under ERK1/2 activation (Fig. 5G). The protein levels of IRF-8 were elevated in the mutant group, whereas they remained decreased in the WT group upon ERK1/2 activation (Fig. 5H). Cumulatively, the data suggest that TRIM63 mediates IRF-8 ubiquitination, leading to a reduction in its protein abundance.

### TRIM63-induced IRF-8 degradation enhances tumor progression and immunosuppression

**Fig. 6:**
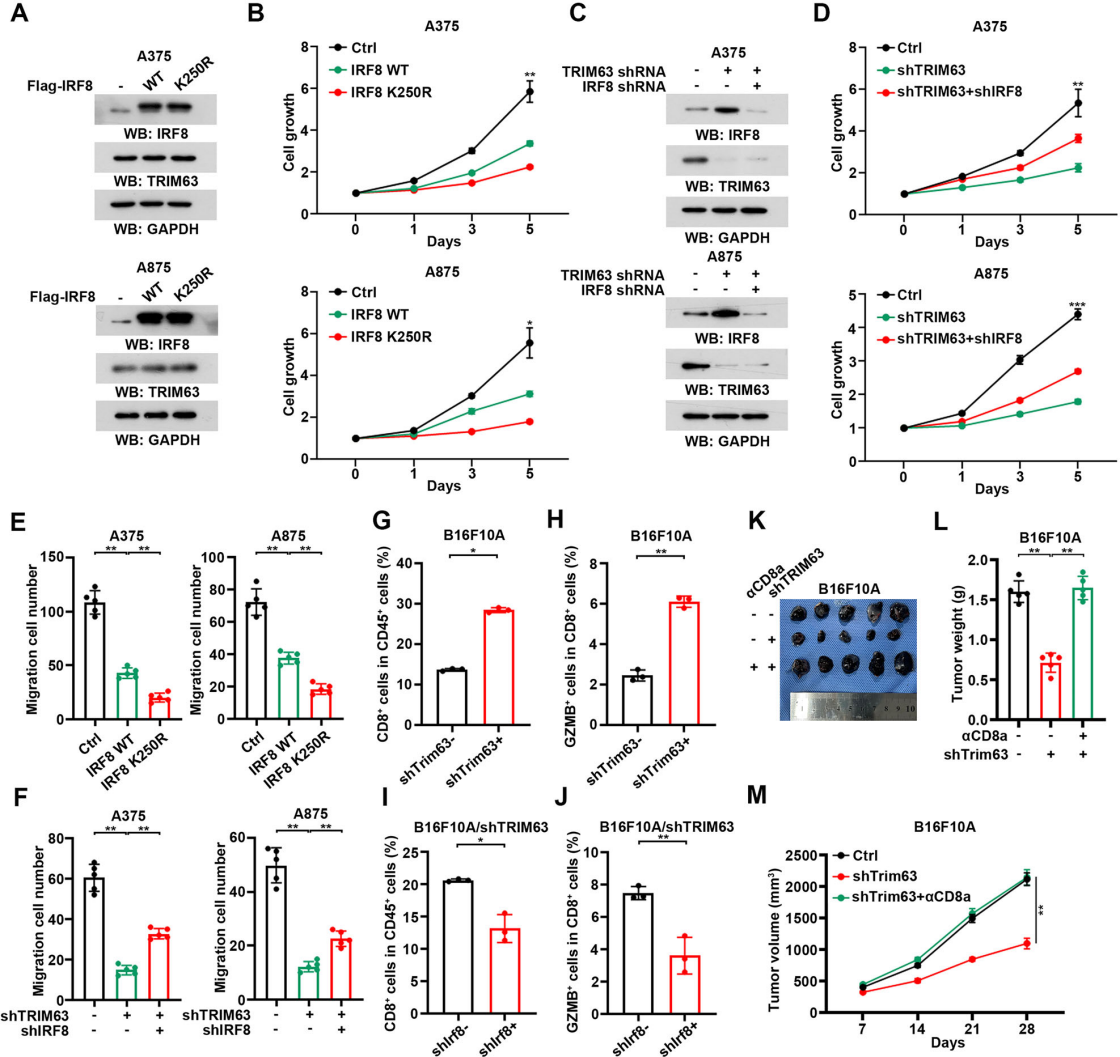
TRIM63-induced IRF-8 degradation enhances tumor progression and immunosuppression. **A**, **B** A375 and A875 cells, treated with MG132, were transfected with Flag-IRF8 WT or K250R mutant. **A** Immunoblotting analyses were performed with the indicated antibodies. **B** The growth of cells was measured by CCK8-assay. Cell growth was expressed as optical density (OD) values. Values were standardized by 0 day. **P < 0.001, *P < 0.05 C-D. A375 and A875 cells were transfected with negative control shRNA or TRIM63 shRNA together transfected with IRF8 shRNA or not. **C** Immunoblotting analyses were performed with the indicated antibodies. **D** The growth of cells was measured by CCK8-assay. Cell growth was expressed as optical density (OD) values. Values were standardized by 0 day. **P < 0.001. **E** A375 and A875 cells were transfected with Flag-IRF8 WT or K250R mutant. The migration ability of A375 and A875 cells was measured by the migration assay. Histograms represent the analysis of the migration assay. Data were presented as mean ± SD (*n* = 5). **P < 0.001. **F** A375 and A875 cells were transfected with negative control shRNA or TRIM63 shRNA together transfected with IRF8 shRNA or not. The migration ability of A375 and A875 cells were measured by the migration assay. Histograms represent the analysis of the migration assay. Data were presented as mean ± SD (*n* = 5). ** P < 0.001. G-H. C57BL/6 mice were subcutaneously injected with shCtrl and shTrim63 B16F10A cells. The percentage of CD8^+^ T cells (**G**) and GZMB production (**H**) were measured. Data were presented as mean ± SD (*n* = 3). ** *P* < 0.001. **I**-**J**. C57BL/6 mice were subcutaneously injected with shCtrl and shIrf8 B16F10A cells depleted with Trim63. The percentage of CD8^+^ T cells (**I**) and GZMB production (**J**) were measured. Data were presented as mean ± SD (*n* = 3). ** *P* < 0.001. K-M. C57BL/6 mice were subcutaneously injected with shCtrl and shTrim63 B16F10A cells and treated with αCD8a antibody (200 μg/mouse, twice a week, *n* = 5). **K** Images of tumor in mice were shown. **L** Bar graph showing mean tumor weight ± standard deviation (SD), ** P < 0.001. (**M**) The growth of tumors was counted by tumor volume, **P < 0.001.

To investigate the role of TRIM63/IRF-8 axis, we primary attempted to check the effect of IRF-8 in tumor cells. Wild-type IRF-8 and the K250R mutant, a ubiquitination-defective variant, were overexpressed in BRAF-mutant melanoma cell lines (Fig. 6A). Our results demonstrated that IRF-8 overexpression significantly suppressed cell proliferation. Notably, the overexpression of the K250R mutant of IRF-8 exhibited a more pronounced inhibitory effect on melanoma cell proliferation compared to the wild-type protein (Fig. 6B). Then, TRIM63 knock-down was performed, resulting in the inhibition of cell proliferation. When both IRF8 and TRIM63 were simultaneously knocked down, the cell proliferation abilities were partially restored, suggesting that the TRIM63/IRF8 axis plays a critical role in promoting melanoma cell proliferation (Fig. 6C, D). Additionally, migration ability was significantly impaired by the overexpression IRF-8 WT and K250R (Fig. 6E, Supplementary Fig. 3A). The migratory defects caused by TRIM63 deletion could also be partially rescued by the knockdown of IRF-8 (Fig. 6F, Supplementary Fig. 3B). These findings collectively indicate that the TRIM64/IRF-8 axis plays an oncogenic signaling role in tumor cells.

To check the effect of TRIM63/IRF-8 axis on tumor immunity. We diminished Trim63 in B16F10A cells by shRNA transfection (Supplementary Fig. 3C). The deletion of Trim63 resulted in enhanced recruitment of CD8^+^ T cells and a more potent immune response (Fig. 6G, H, Supplementary Fig. 3D, E). As expected, both the weight and volume of tumor growth were significantly decreased following Trim63 knock-down (Supplementary Fig. 3F–H). Moreover, the abrogation of Irf-8 within the context of Trim63 deletion eliminated the beneficial effects on both the recruitment and functional capabilities of CD8^+^ T cells (Fig. 6I, J, Supplementary Fig. 3I–K). Also, the decreased tumor weight and volume were abolished after Irf-8 knock-down (Supplementary Fig. 3L–N). In addition, we have performed reversion experiments with the treatment of αCD8. Notably, the immunodepleting of CD8^+^ T cells eliminates the benefit of TRIM63-depletion on tumor growth (Fig. 6K–M). Thus, these findings demonstrated that TRIM63 promotes the growth of BRAF mutant melanoma by suppression of CD8^+^ T cells.

In summary, the findings from our data analysis suggest that the regulatory axis of TRIM63/IRF-8 plays a significant role in promoting tumor progression, both within tumor cells and the immune microenvironment.

### TRIM63 pS69 correlates with IRF-8 protein levels and poor prognosis of melanoma patients

**Fig. 7:**
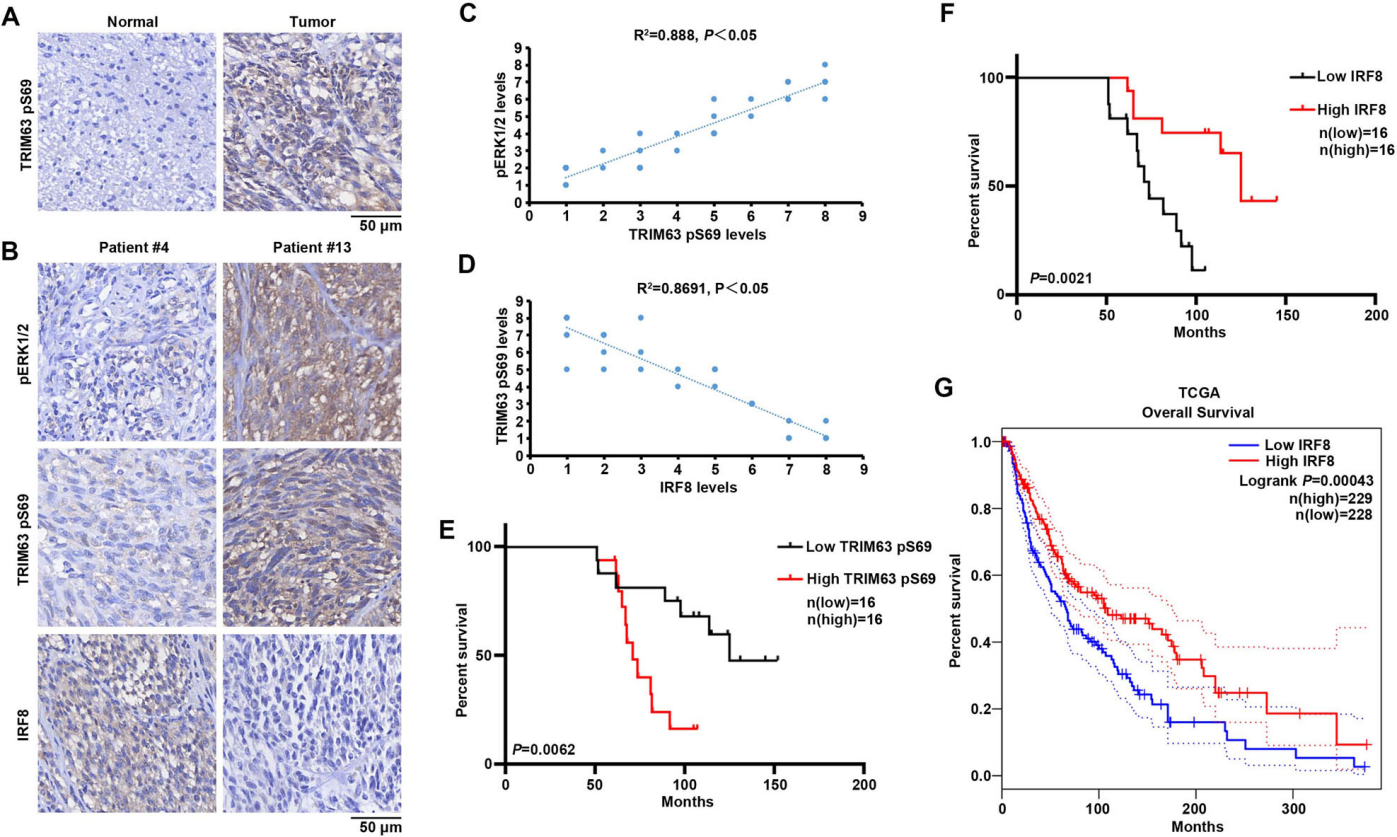
TRIM63 pS69 correlates with IRF-8 protein levels and poor prognosis of melanoma patients. **A** Immunohistochemistry analyses of TRIM63 pS69 expression levels in normal skin and melanoma tissues. **B** Immunohistochemistry analyses of pERK1/2, TRIM63 pS69 and IRF8 expression levels in melanoma tissues. The number represents the patient identification. **C** Correlation between levels of pERK1/2 and TRIM63 pS69 in 32 melanoma patients. Pearson correlation tests were performed, *P* < 0.05. Note that some of the dots represented more than one specimen. **D** Correlation between levels of TRIM63 pS69 and IRF8 in 32 melanoma patients. Pearson correlation tests were performed, *P* < 0.05. Note that some of the dots represented more than one specimen. **E** Kaplan–Meier survival analysis of 32 collected clinical melanoma samples based on TRIM63 pS69 IHC score, High TRIM63 pS69 (*n* = 16), Low TRIM63 pS69 (*n* = 16). **F** Kaplan–Meier survival analysis of 32 collected clinical melanoma samples based on IRF8 IHC score, High IRF8 (*n* = 16), Low IRF8 (*n* = 16). **G** Kaplan-Meier curves for overall survival (OS) of patients with SKCM stratified by IRF8 mRNA expression levels. Patients from TCGA SKCM cohort were divided into high (*n* = 229) and low (*n* = 228) IRF8 expression groups using the median expression level as the cutoff. Statistical significance was determined by the log-rank test (*P* = 0.0043).

To evaluate the clinical significance of TRIM63 pS69, we performed immunohistochemical staining on both normal human skin and melanoma tissues using an anti-TRIM63 pS69 antibody. Our results demonstrated that TRIM63 pS69 was barely detectable in normal skin samples, whereas it was significantly overexpressed in melanoma samples (Fig. 7A). Further, the signal was robustly detected in melanoma samples and exhibited a positive correlation with phosphorylation levels of ERK1/2, while displaying a negative correlation with IRF-8. (Fig. 7B). Quantification of the IHC staining indicated that these correlations were significant (Fig. 7C, D). Notably, high levels of TRIM63 pS69 were correlated with decreased overall durations of survival in melanoma patients (Fig. 7E). IRF-8 has been identified as a potential tumor suppressor in the progression of melanoma [25]. We also observed that higher expression levels of IRF-8 are associated with a better prognosis (Fig. 7F). The consistent results were also validated in the TCGA database, demonstrating that higher IRF-8 levels are associated with improved survival duration (Fig. 7G). In conclusion, these results suggested that the regulatory axis of TRIM63 pS69/IRF-8 plays a critical role in clinical outcomes of human melanoma.

## Discussion

In this study, we elucidate a novel mechanism by which BRAF mutations activate the MAPK signaling pathway, leading to ERK1/2 activation and subsequent phosphorylation of TRIM63 at S69, thereby unleashing its complete oncogenic potential. Moreover, TRIM63, an E3 ubiquitin ligase, ultimately associates with IRF-8 resulting in degradation of the tumor suppressor gene to promote tumor progression. This study identifies a novel oncogene, TRIM63, and elucidates its functional mechanisms that drive melanoma progression associated with BRAF mutations commonly observed in melanoma.

Several TRIM family members have been reported acting critical roles in melanoma progression [29, 30]. TRIM17 and TRIM28 are identified as antagonistic regulators that maintain the balance of BCL2A2 protein levels, thereby modulating melanoma progression [29]. TRIM28 mediates the ubiquitination-driven degradation of BCL2A2, whereas TRIM17 stabilizes TRIM63 by inhibiting the interaction between TRIM28 and BCL2A2 [29]. In addition, TRIM14, another member of the TRIM protein family, plays a significant role in regulating melanoma malignancy by impairing the anti-tumor function of PTEN [30]. Those studies suggest that TRIM family members may serve as a pivotal factor in the progression of melanoma, highlighting their critical role in this complex biological process. Here, we observe that TRIM63 is another oncogenic TRIM protein in melanoma progression, highly associated with poor prognosis of patients. Also, TRIM63 is a key regulator in many other types of cancer, such as lung cancer, breast cancer, and kidney cancer [12, 31, 32]. TRIM63 has been proven to conduct its function via WNT/β-catenin signaling [31]. The underlying mechanisms of TRIM63 in melanoma are still uncharted. One of the critical contributions of our research is the identification of the TRIM63 performs its oncogenic role by the degradation of IRF-8 in the particular cellular condition of BRAF mutation. Meanwhile, BRAF is the most predominant genetic mutation in melanoma [33, 34]. Previous literature has documented the activation of the MAPK pathway following BRAF mutations, primarily focusing on its role in promoting cell proliferation and survival [35]. However, the downstream effects, especially ubiquitination regulation of key factors, have not been extensively characterized. This finding fills a notable gap in the understanding of how BRAF-driven signaling can subvert the process of tumor suppressor gene ubiquitination in melanoma.

Furthermore, IRF-8 is a critical transcription factor governing diverse immune cell functions. It has been acknowledged for its role in modulating immune responses and regulating apoptosis, serving as a pivotal component in tumor immunity [36]. In physiological conditions, IRF-8 is predominantly expressed in immune cells such as dendritic cells, macrophages, and T cells, where it governs the development and functionality of these cells [37, 38]. In dendritic cells (DCs), IRF-8 is essential for plasmacytoid DC development and type I interferon (IFN-I) production upon viral sensing, driving antiviral and antitumor activity [39]. For macrophages, IRF-8 promotes classical (M1) activation, enhancing pro-inflammatory cytokine production (e.g., IL-12, TNF-α) and microbicidal responses [21]. In B cells, IRF-8 regulates germinal center formation, isotype class switching, and plasma cell differentiation [40]. Within T cells, IRF-8 modulates CD8^+^ T cell effector functions and helps balance Th1/Th17 differentiation [41]. In the context of tumor immunity, IRF-8 enhances the presentation of tumor antigens by promoting T cell activation and fostering a robust anti-tumor immune response [42, 43]. It accomplishes this by modulating the expression of crucial genes involved in immune signaling pathways, including cytokine production and upregulation of major histocompatibility complex (MHC) molecules [43]. This facilitates improved recognition of cancer cells by the immune system [44]. Low levels of IRF-8 have been strongly associated with tumor immunosuppression. However, the precise role of IRF-8 in tumor cells remains unclear. Herein, we identify that TRIM63-mediated degradation of IRF-8 provides a mechanistic explanation for observed immune evasion in numerous melanoma cases. This novel perspective sheds light on how melanoma cells may persist despite undergoing immune surveillance.

In conclusion, this study significantly enhances our understanding of the molecular mechanisms underlying BRAF-driven melanoma progression by elucidating the TRIM63/IRF-8 axis. However, further research is needed to fully explore the implications of these findings, particularly regarding therapeutic strategies and melanoma heterogeneity. As we continue to unravel these complexities, we move closer to developing more effective treatments for patients with this challenging cancer.

## Materials and methods

### Materials

MG132 (S2619) and Cycloheximide (S7418) were from Selleckchem (Boston, MA, USA). Anti-mouse and anti-rabbit secondary antibodies were purchased from Santa Cruz Biotechnology (Santa Cruz, CA, USA). Rabbit polyclonal antibodies against TRIM63 S69 phosphorylation (TRIM63 pS69) were produced by Affinity Biosciences LTD (Cincinnati, OH, USA). FITC anti-CD45 (103107), PE anti-CD8 (140408), APC anti-GZMB (396407,) and MojoSort™ Mouse CD45 Nanobeads (480027) were purchased from Biolegend (San Diego, CA, USA). Anti-Flag M2 Affinity Gels (A2220), streptavidin agarose (S1638), and anti-pan Phospho-Serine/Threonine (SAB5701877) were purchased from Sigma-Aldrich (Shanghai, China). HisPur™ Ni-NTA Resin and Fixable Viability Dye eFluor™ 780 (65-0865-14) was purchased from ThermoFisher Scientific (Waltham, MA, USA). Anti-TRIM63 (55456-1-AP), anti-IRF-8 (18977-1-AP), and GAPDH (60004-1-Ig) were purchased from Proteintech (Wuhan, China). Anti-Flag (DYKDDDDK) tag (F1804) was purchased from Sigma-Aldrich (Shanghai, China). Anti-ubiquitin (58395), anti-pERK1/2 (9101,) and anti-ERK1/2 (137F5) were purchased from Cell Signaling Technology (Beverly, MA, USA). The other chemicals utilized in the present study were obtained from a commonly available commercial source.

### Experimental animals

The C57BL/6 and BALB/c-Nude mice, aged 6 weeks, were obtained from SLAC Laboratory Animal Co., Ltd in Shanghai, China. They were housed in a Specific Pathogen Free (SPF) animal facility and provided with sterilized food and water. Human- or murine-derived melanoma cells (1 × 10^5^) were subcutaneously injected into the mice. After 7 days of injection, the mice were randomly assigned to their respective experimental groups. Tumor volumes were measured at regular intervals using a caliper after tumor formation. The dimensions of the tumors were recorded by measuring their length (L) and width (W). The tumor volume (V) was calculated using the formula: V = (L × W²) / 2, where L represents the longest dimension and W represents the shortest dimension. For tumor weight, after euthanizing the tumor-bearing mice, we completely removed the tumors, then washed away the bloodstains with physiological saline, and finally dried the excess saline and weighed them.

### Cell culture and transfection

The human-derived cell lines A375, A875, A2058, SK-MEL-1, CHL-1, C8161, and HEK293T cells, as well as the mouse-derived B16-F10 cells, were obtained from American Type Culture Collection (ATCC) and cultured in DMEM high glucose medium supplemented with 10% fetal bovine serum and 1% penicillin/streptomycin (Gibco). The cells were cultured at a temperature of 37 °C with a CO_2_ concentration of 5%. Cells were plated at a density of 4 × 10^5^ per 60-mm dish or 1 × 10^5^ per well of a 6-well plate 18 h before transfection. Transfection was performed using polyjet transfection reagent (SignaGen Laboratories) according to the manufacturer’s instructions. Briefly, prepare complexes by diluting DNA in serum-free or reduced-serum medium. Separately, dilute the appropriate volume of PolyJet Reagent in the same medium. Combine the diluted PolyJet with the diluted DNA, immediately vortex or pipette mix, and incubate at room temperature for 15–30 minutes to form transfection complexes. Gently add the DNA-PolyJet complexes dropwise onto cells in their culture medium (with or without serum). Gently rock the plate and return cells to the incubator. After 24 hours, replace the medium with fresh complete growth medium if needed. Analyze gene expression 24–72 hours post-transfection.

### Cell viability assay

The indicated Cells were seeded into 96-well culture plates at a density of 5000 per well for 24 h. After indicated treatments, cell viability was examined by Cell Counting Kit-8 (Dojindo). Briefly, add CCK-8 reagent directly to each well, using 10% of the original culture medium volume. Gently mix the plate by tapping or swirling carefully to avoid bubbles. Incubate the plate at 37 °C in a humidified CO₂ incubator for 30 min. Protect the plate from light during incubation. Measure the absorbance at 450 nm using a microplate reader.

### Cell migration assay

Cell migration was assessed using 24-well chambers with an 8 μm pore size (Corning, USA). The upper chamber was seeded with cells (5 × 10^4^/well) in 100 μL DMEM without FBS, while the lower chamber contained 500 μL DMEM supplemented with 10% FBS to promote cell migration. Following a 24-hour incubation period, non-migrated cells were removed using a cotton swab, and the bottom cells were fixed with 3% paraformaldehyde. These cells were then stained with crystal violet (0.1%) and imaged in three independent fields per well at a magnification of x10. Membranes were air-dried, and fixed cells were eluted for decolorization by adding acetic acid (33%, 200 μL/well) for fifteen minutes at room temperature. The resulting solution was transferred to a microplate reader (BMG Labtech, Germany), where absorbance values were measured at a wavelength of 570 nm from each well on a separate plate.

### Mass spectrometry analysis

Immunoprecipitated TRIM63 protein and its binding profile were purified. For digestion, the protein solution was reduced with 5 mM dithiothreitol for 30 min at 56 °C and alkylated with 11 mM iodoacetamide for 15 min at room temperature in darkness. The protein sample was then diluted by adding 100 mM TEAB to a urea concentration less than 2 M. Finally, chymotrypsin was added at a 1:50 chymotrypsin-to-protein mass ratio for the first digestion overnight and a 1:50 chymotrypsin-to-protein mass ratio for a second 4 h-digestion. Following chymotryptic digestion, the prepared peptides were analyzed by MALDI-TOF/TOF MS (MALDI-7090, Shimadzu Kratos). The peptide mass fingerprints and peptide ion MS/MS spectra were acquired on MALDI-7090. The total MS/MS data were searched against the SwissProt Database using the following parameters: chymotrypsin digestion allowing up to 1 missed cleavage, fixed modifications of cysteine (carbamidomethylation), variable modifications of methionine (oxidation) and cysteine (succination), precursor peptide tolerance of 0.05 Da, and MS/MS tolerance of 0.2 Da. Search results with e values less than 0.01 were judged as positive identifications.

### DNA construction and mutagenesis

Human TRIM63 and its variants obtained through PCR amplification were inserted into either pColdI (His) or pcDNA3.1/hygro (+)-Flag vector. Human wild-type TRIM63 and its mutants obtained through PCR amplification were inserted into either pcDNA3.1-Flag or pcDNA3-His vector. The generation of IRF-8 and mutated versions of TRIM63 was accomplished using the QuikChange site-directed mutagenesis kit from Stratagene in La Jolla, CA, USA.

### Purification of recombinant proteins

The purification of 6×His-tagged proteins was performed using BL21 (DE3) cells. Bacteria expressing TRIM63 with a His-tag were cultured in 400 ml lysogeny broth (LB) medium supplemented with ampicillin at a ratio of 1:1000. Upon reaching an OD value of 0.4–0.6, the expression of targeted proteins was induced by adding 0.5 mM isopropyl β-D-1-thiogalactopyranoside (IPTG, Sigma-Aldrich) and incubating overnight at a temperature of 16 °C. The following day, the bacteria were centrifuged and lysed, and the resulting lysates were then subjected to overnight incubation with Ni-NTA beads (ThermoFisher Scientific) at a temperature of 4 °C. To remove non-specific binding proteins, the mixture was washed eight times with a solution containing 35 mM imidazole before eluting the purified proteins using a solution containing 350 mM imidazole. Finally, higher concentration proteins were obtained by condensing them using spin columns with cut-off sizes of either 3-kDa or 10-kDa (Millipore). For Flag-tagged proteins, HEK293T cells were transfected with TRIM63 tagged with Flag and subsequently lysed for immunoprecipitation using anti-Flag affinity gels. After washing five times with PBS, competitive elution was performed to acquire the targeted Flag-tagged proteins by utilizing PBS containing Flag peptide at a concentration of 500 mg/ml (Sigma-Aldrich).

### Immunoprecipitation and immunoblotting analysis

The proteins were extracted from cultured cells using a modified buffer containing 50 mM Tris-HCl (pH 7.5), 0.01% SDS, 1% Triton X-100, 150 mM NaCl, 1 mM dithiothreitol, 0.5 mM EDTA, 1% proteinase inhibitor cocktail, and additional components such as sodium orthovanadate (100 μM), sodium pyrophosphate (100 μM), and sodium fluoride (1 mM). Finally, immunoprecipitation and immunoblot analyses were conducted using appropriate antibodies.

### Tissue dissociation and flow cytometry analysis

B16-F10 cell-derived xenograft mice in different groups were perfused transcardially with cold phosphate-buffered saline (PBS) to clear away blood cells from tumor tissues. The tumor tissues were dissociated enzymatically to obtain a single-cell suspension. The cell suspension was filtered through a 70 μm strainer and centrifuged at 500×g, 4 °C for 10 min. Next, tumor cells and fibroblast cells were removed by centrifugation on a 30% Percoll gradient (No. 17089101, GE Healthcare). Cell suspension was centrifuged at 2000 rpm, 4 °C for 30 min without acceleration and brakes. Finally, cells were collected for flow cytometric analysis. Cells were incubated for 15 min with Mouse BD Fc Block™ (BD, 553141) in FACS Buffer to block FcγRIII/II and reduce unspecific antibody binding. For surface marker analysis, cells were re-suspended in FACS buffer and stained with FITC anti-CD45, PE anti-CD8 at 4 °C for 30 min. For intracellular staining, the cells were fixed and permeabilized using a fixation/permeabilization solution (BD Cytofix/Cytoperm), as per the instructions provided by the manufacturer. Subsequently, they were stained with APC anti-GZMB. Data were acquired by the BD FACSymphony A5 and analyzed with FlowJo software.

### Quantification and statistical analysis

Unless specifically indicated, all statistical analyses were conducted with a two-tailed unpaired Student’s t test. All data represent the mean ± SD of three independent experiments/samples unless otherwise specified. p < 0.05 was considered statistically significant. Analyses were performed using Microsoft Excel or GraphPad software.

## Supplementary information

Fig. S1

Fig. S2

Fig. S3

Supplementary figure legend

Raw data (wb)
