# Supplementary figures and images for: TRIM63/IRF-8 axis promotes tumor progression and immunosuppression of melanoma with BRAF mutation
Source: Cell Death Dis. 2025 Nov 28;16(1):869. doi: 10.1038/s41419-025-08216-5 (PMC12663380; doi:10.1038/s41419-025-08216-5)

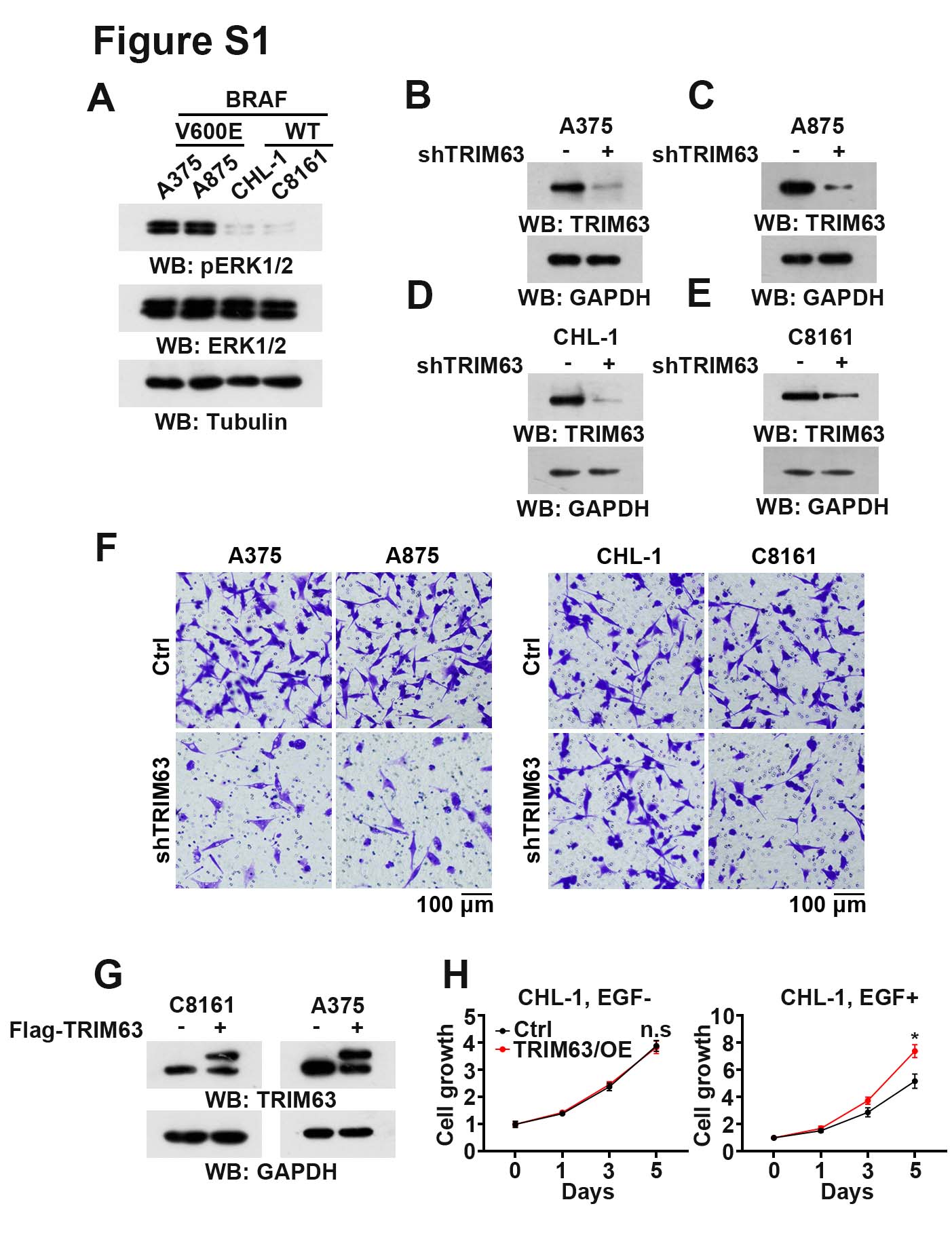

Supplement: Supplementary file 1 — Fig. S1 [file 41419_2025_8216_MOESM1_ESM.jpg]

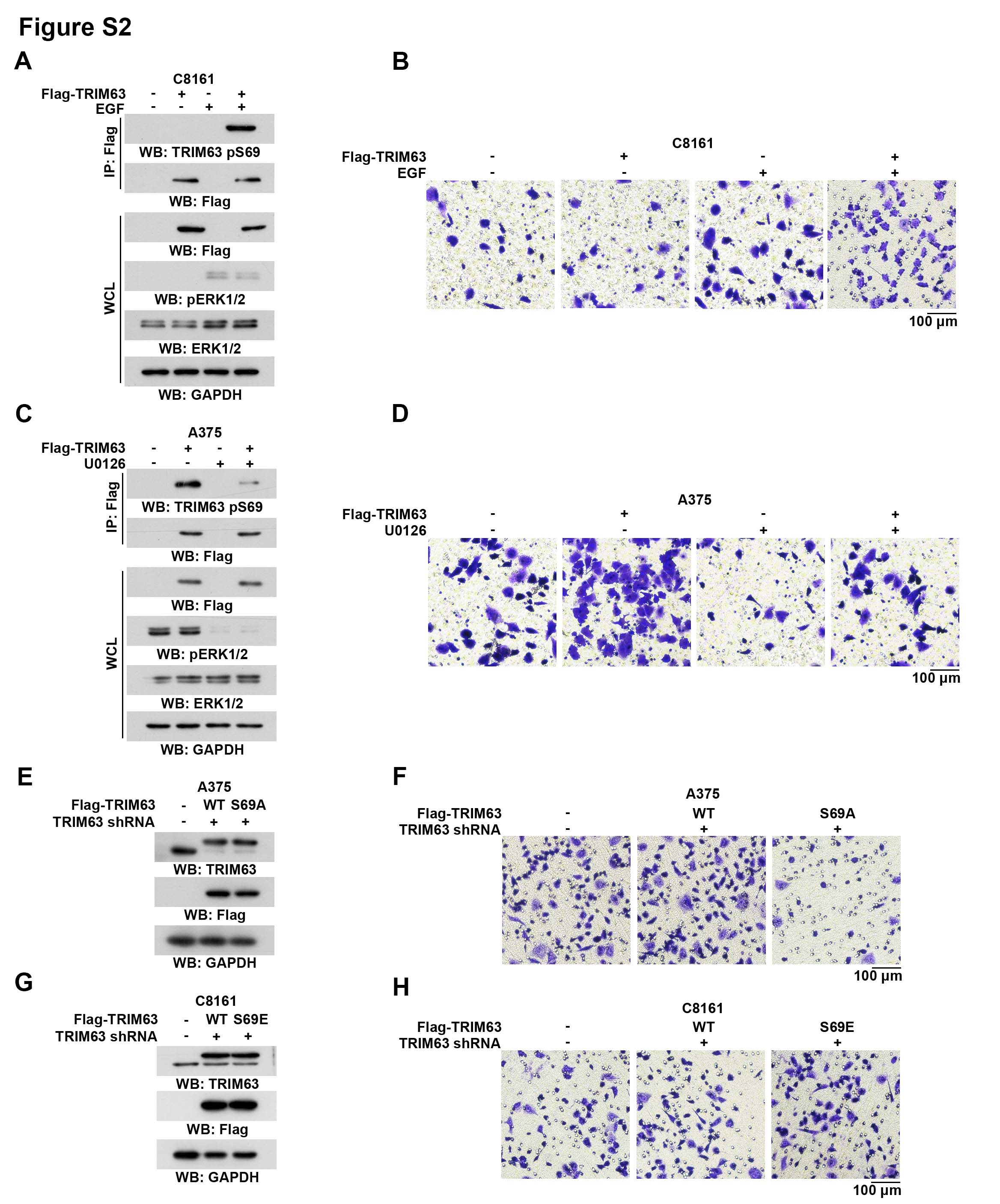

Supplement: Supplementary file 2 — Fig. S2 [file 41419_2025_8216_MOESM2_ESM.jpg]

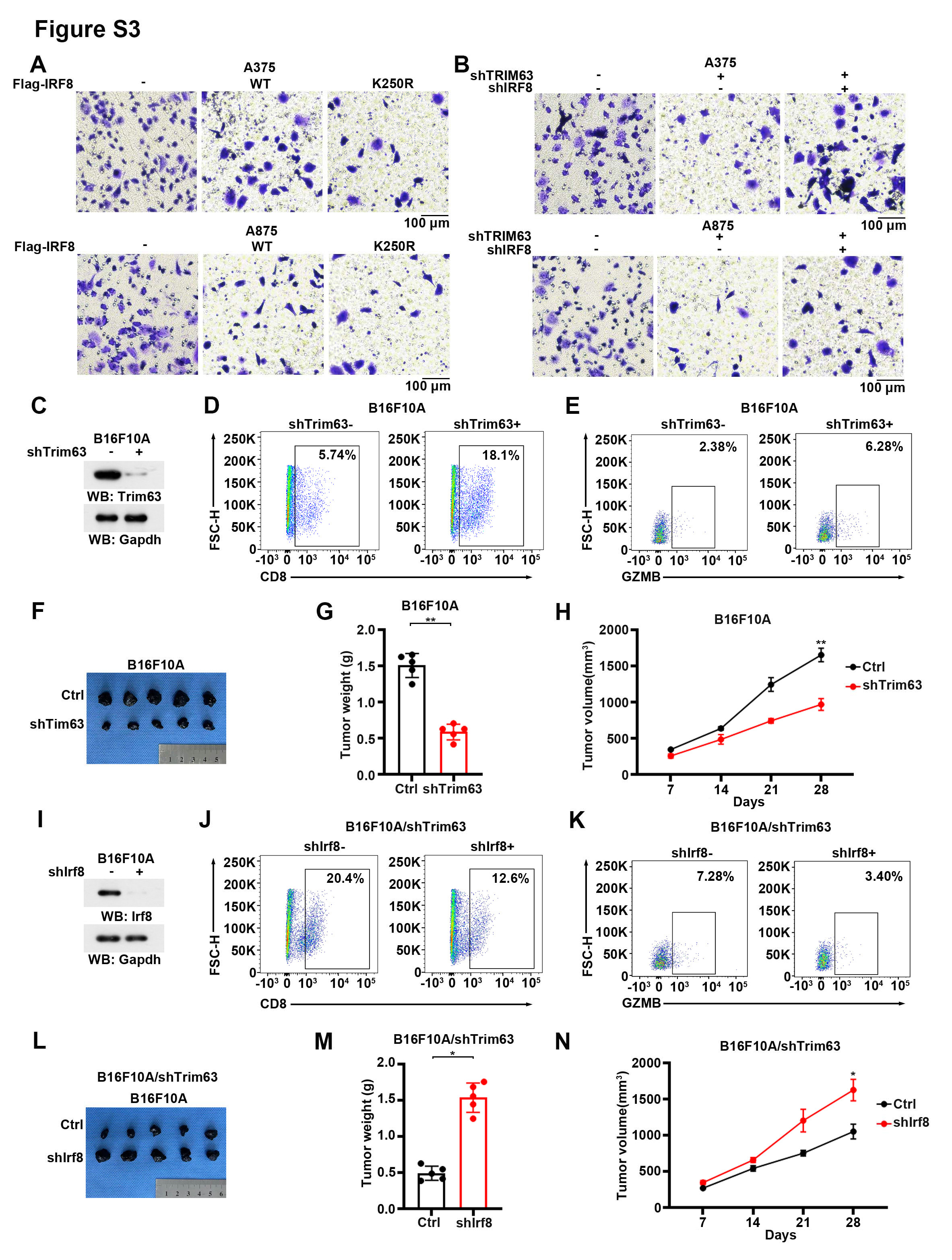

Supplement: Supplementary file 3 — Fig. S3 [file 41419_2025_8216_MOESM3_ESM.jpg]
