# Supplementary material for: TRIM63/IRF-8 axis promotes tumor progression and immunosuppression of melanoma with BRAF mutation
Source: Cell Death Dis. 2025 Nov 28;16(1):869. doi: 10.1038/s41419-025-08216-5 (PMC12663380; doi:10.1038/s41419-025-08216-5)
Supplement: Supplementary file 4 — Supplementary figure legend [file 41419_2025_8216_MOESM4_ESM.docx]

**Supplementary figure 1. Referred to figure 1.**

A. 1×10^6^ A375 (BRAF V600E), A875 (BRAF V600E), CHL-1 (BRAF WT) and C8161 (BRAF WT) cells were lysed. Immunoblotting analyses were performed with the indicated antibodies. pERK1/2 expression levels were higher in BRAF mutant cells.

B-E. A375 (BRAF V600E), A875 (BRAF V600E), CHL-1 (BRAF WT) and C8161 (BRAF WT) cells were transfected with negative control shRNA or TRIM63 shRNA and lysed. Immunoblotting analyses were performed with the indicated antibodies. TRIM63 expression levels were reduced after treated with TRIM63 shRNA. F. Depletion of TRIM63 in A375, A875, CHL-1 and C8161 cells and measured the migration ability of these cells by migration assay. Representative images were shown, scale bar, 100 μm.

G. 1×10^6^ C8161 and A375 cells were transfected with Flag-TRIM63 (2 μg) for 72 h. Immunoblotting analyses were performed with the indicated antibodies.

H. Cell growth assessed by CCK-8 assay in CHL-1 cells expressing or not expressing Flag-TRIM63 without EGF treatment (left). Cell growth assessed by CCK-8 assay in CHL-1 cells expressing or not expressing Flag-TRIM63 following EGF treatment (right).

**Supplementary figure 2. Referred to figure 3.**

A. C8161 cells were transfected with or without Flag-TRIM63 and treated with or without EGF (100ng/ml). Immunoprecipitation was performed by incubating the Flag antibodies (10 mg) with 1 mg of cell lysates at 4 °C overnight, followed by incubation of protein G-agarose beads for 3 hours. The beads were then washed with lysis buffer for 4 times. Immunoblotting analyses were performed with the indicated antibodies.

B. C8161 cells were transfected with or without Flag-TRIM63 and treated with or without EGF (100ng/ml). C8161 cells and measured the migration ability of these cells by migration assay. Representative images were shown, scale bar, 100 μm.

C. A375 cells transfected with or without Flag-TRIM63 and treated with or without U0126 (20 μM). Immunoprecipitation was performed by incubating the Flag antibodies (10 mg) with 1 mg of cell lysates at 4 °C overnight, followed by incubation of protein G-agarose beads for 3 hours. The beads were then washed with lysis buffer for 4 times. Immunoblotting analyses were performed with the indicated antibodies.

D. A375 cells transfected with or without Flag-TRIM63 and treated with or without U0126 (20 μM). A375 cells and measured the migration ability of these cells by migration assay. Representative images were shown, scale bar, 100 μm.

E. A375 cells transfected with or without TRIM63 shRNA and then transfected with Flag-TRIM63 WT or S69A mutant. Immunoblotting analyses were performed with the indicated antibodies.

F. A375 cells transfected with or without TRIM63 shRNA and then transfected with Flag-TRIM63 WT or S69A mutant. A375 cells and measured the migration ability of these cells by migration assay. Representative images were shown, scale bar, 100 μm.

G. C8161 cells transfected with or without TRIM63 shRNA and then transfected with Flag-TRIM63 WT or S69E mutant. Immunoblotting analyses were performed with the indicated antibodies.

H. C8161 cells transfected with or without TRIM63 shRNA and then transfected with Flag-TRIM63 WT or S69E mutant. C8161 cells and measured the migration ability of these cells by migration assay. Representative images were shown, scale bar, 100 μm.

**Supplementary figure 3. Referred to figure 6.**

A. A375 and A875 cells were transfected with Flag-IRF8 WT or K250R mutant (2 μg). The migration ability of A375 and A875 cells were measured by the migration assay. Representative images were shown.

B. A375 and A875 cells were transfected with TRIM63 shRNA together transfected with IRF8 shRNA or not. The migration ability of A375 and A875 cells were measured by the migration assay. Representative images were shown.

C. B16F10A cells were transfected with Trim63 shRNA . Immunoblotting analyses were performed with the indicated antibodies.

D-E. C57BL/6 mice were subcutaneously injected with shCtrl and shTrim63 B16F10A cells. The FACS profiles of CD8^+^ T cells (D) and GZMB production (E) were shown.

F-H. B16F10A cells (5 × 10^5^) with or without TRIM63 overexpression were subcutaneously injected into C57BL/6 mice (n = 5 per group). Images of tumor in mice were shown (F). Histograms represent the analysis of the tumor weight. ** P <0.001 (G). The growth of tumors was counted by tumor volume (H).

I. B16F10A cells were transfected with Irf8 shRNA. Immunoblotting analyses were performed with the indicated antibodies.

J-K. C57BL/6 mice were subcutaneously injected with shCtrl and shIrf8 B16F10A cells. The FACS profiles of CD8+ T cells (J) and GZMB production (K) were shown.

L-N. B16F10A cells (5 × 10^5^) depletion of Trim63 with or without Irf8 overexpression were subcutaneously injected into C57BL/6 mice (n = 5 per group). Images of tumor in mice were shown (L). Histograms represent the analysis of the tumor weight. ** P <0.001 (M). The growth of tumors was counted by tumor volume (N).
