# Supplementary material for: TRIM63/IRF-8 axis promotes tumor progression and immunosuppression of melanoma with BRAF mutation
Source: Cell Death Dis. 2025 Nov 28;16(1):869. doi: 10.1038/s41419-025-08216-5 (PMC12663380; doi:10.1038/s41419-025-08216-5)

Figure 1F

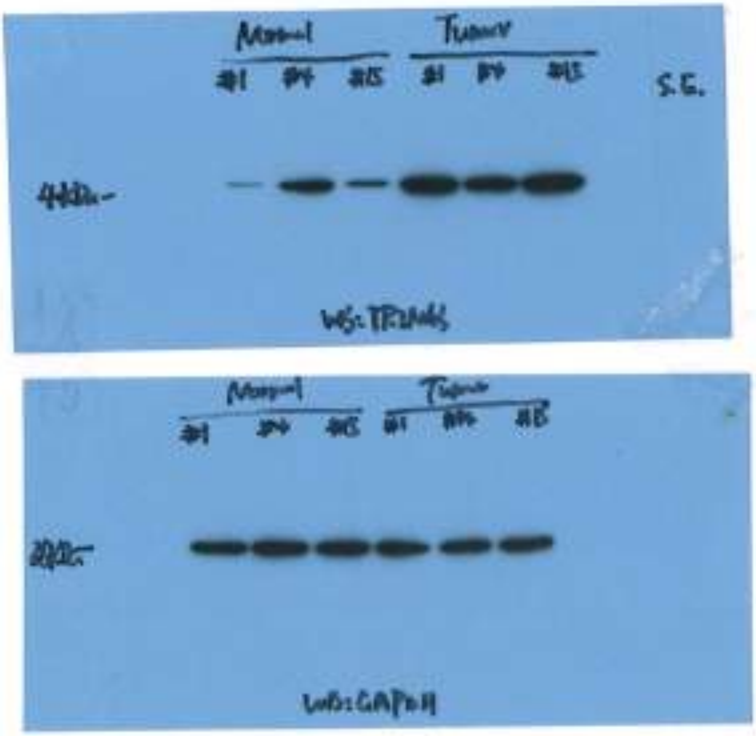

Figure 11

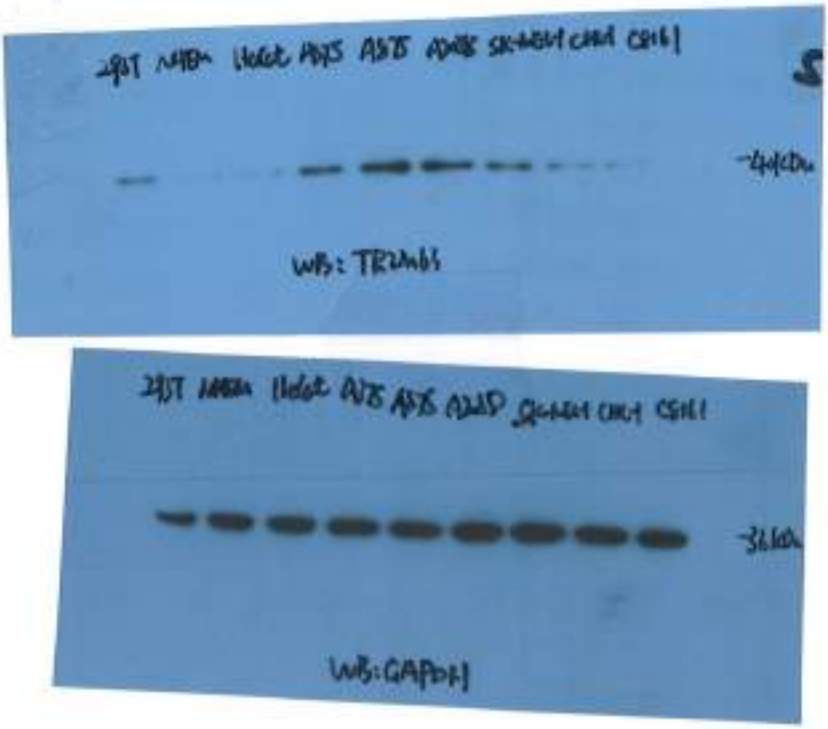

2A

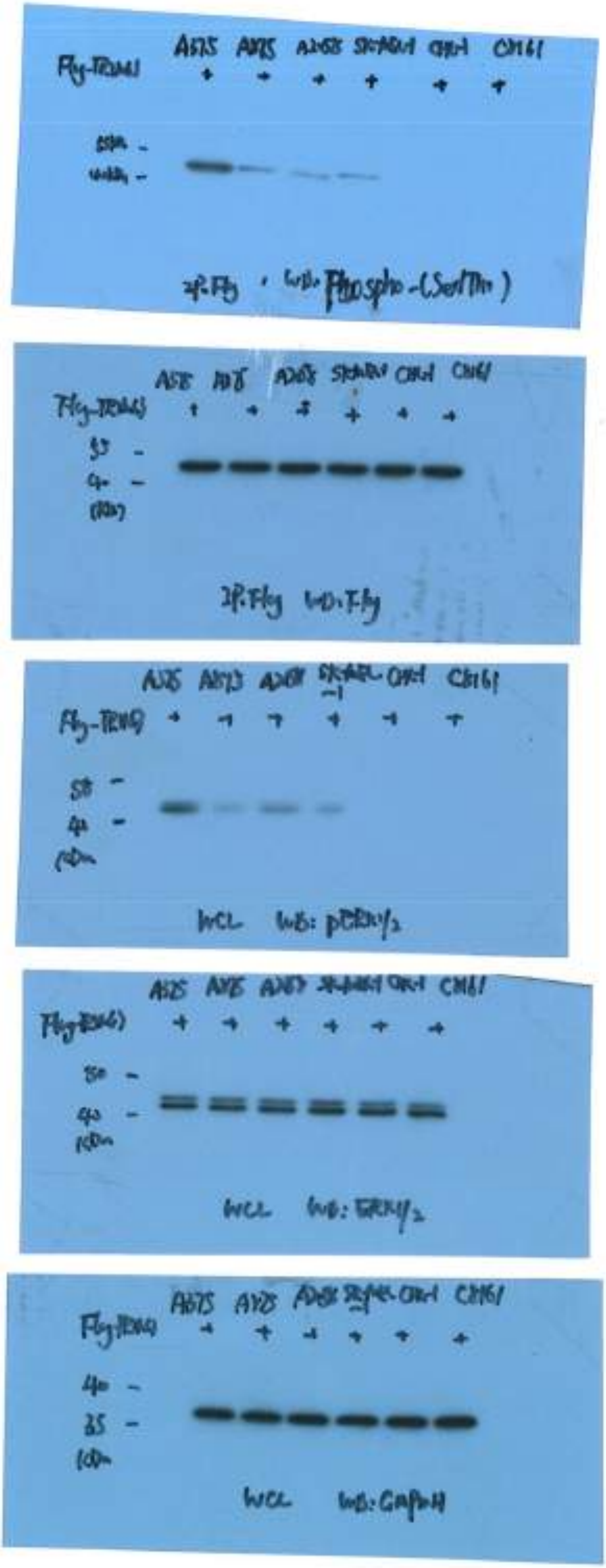

Figure 2A

2B

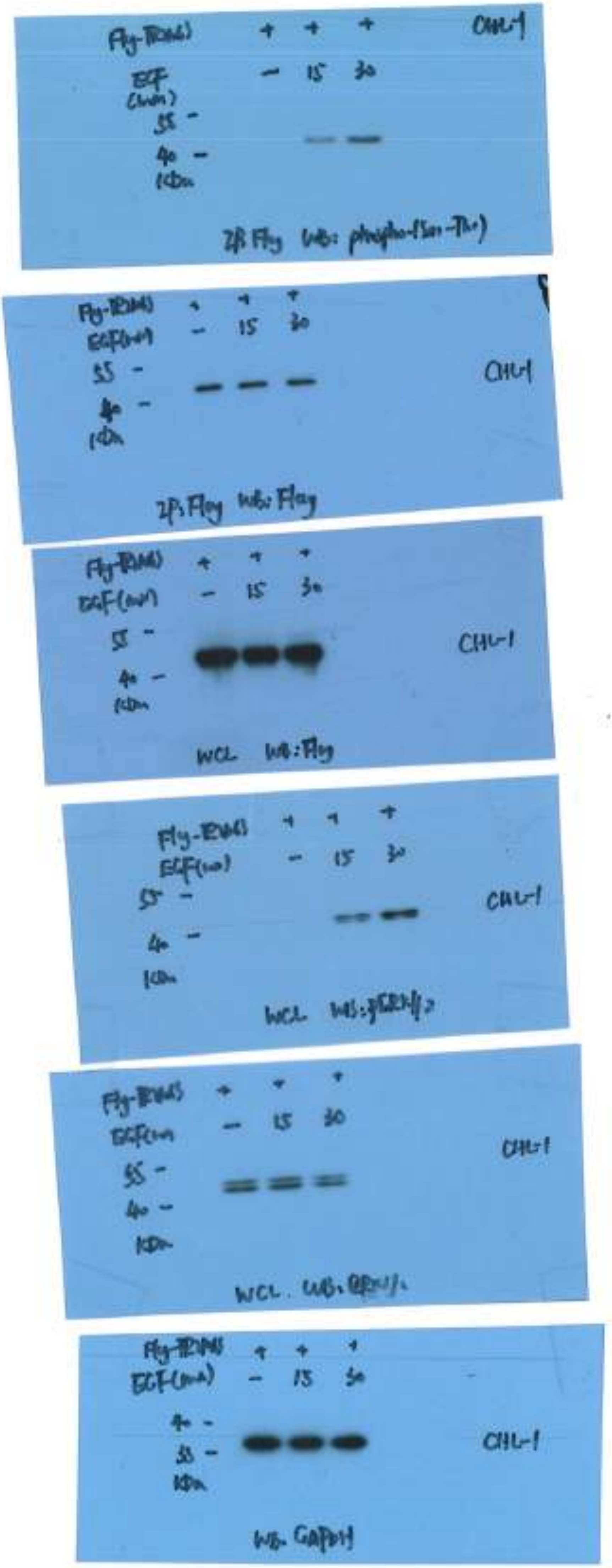

Figure 2B

2C

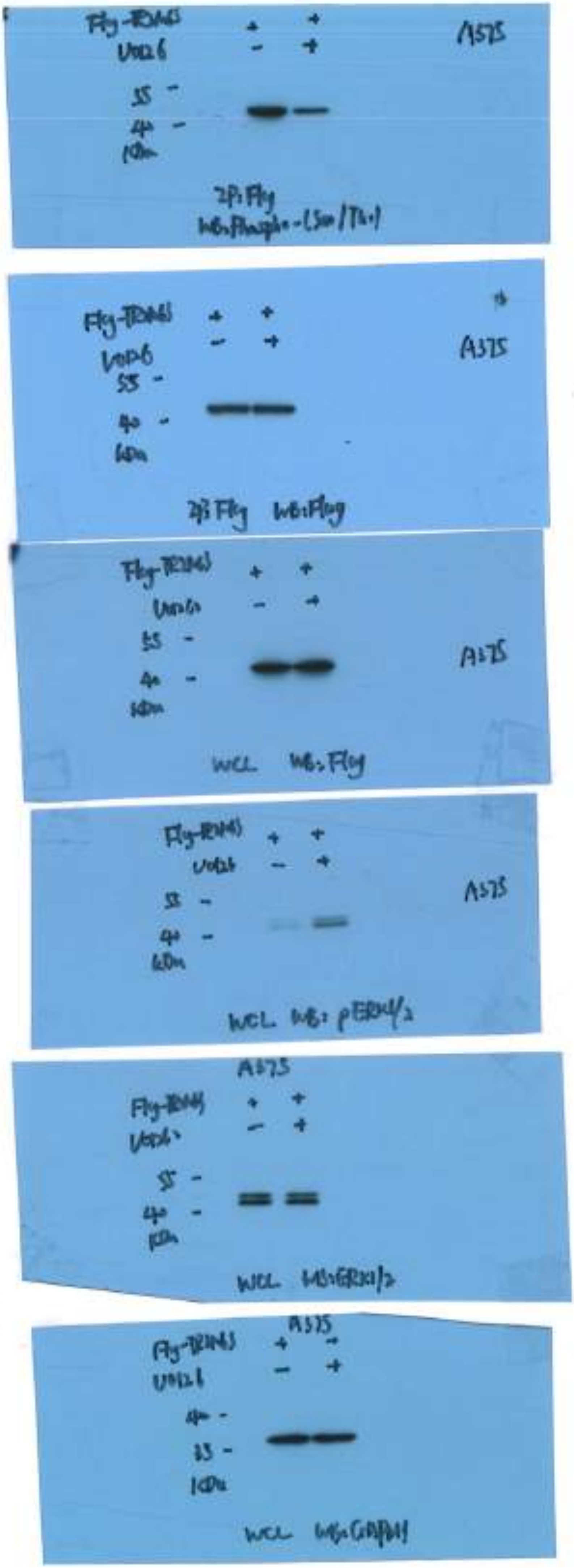

Figure 2C

Fig 2C

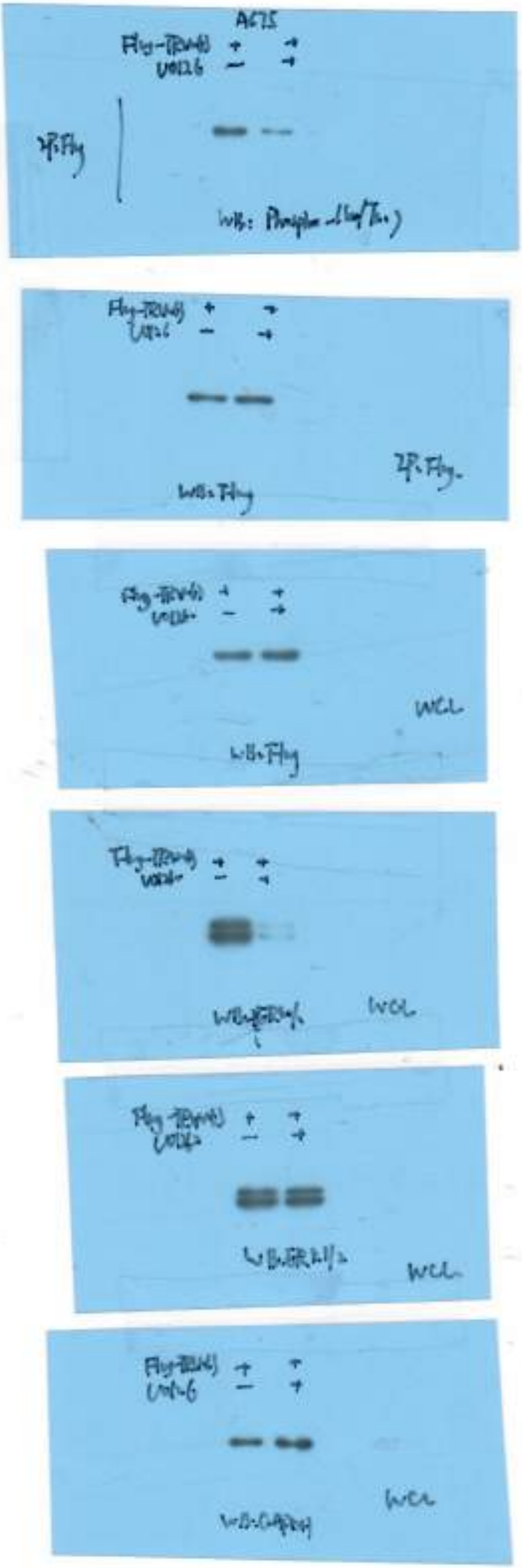

2D

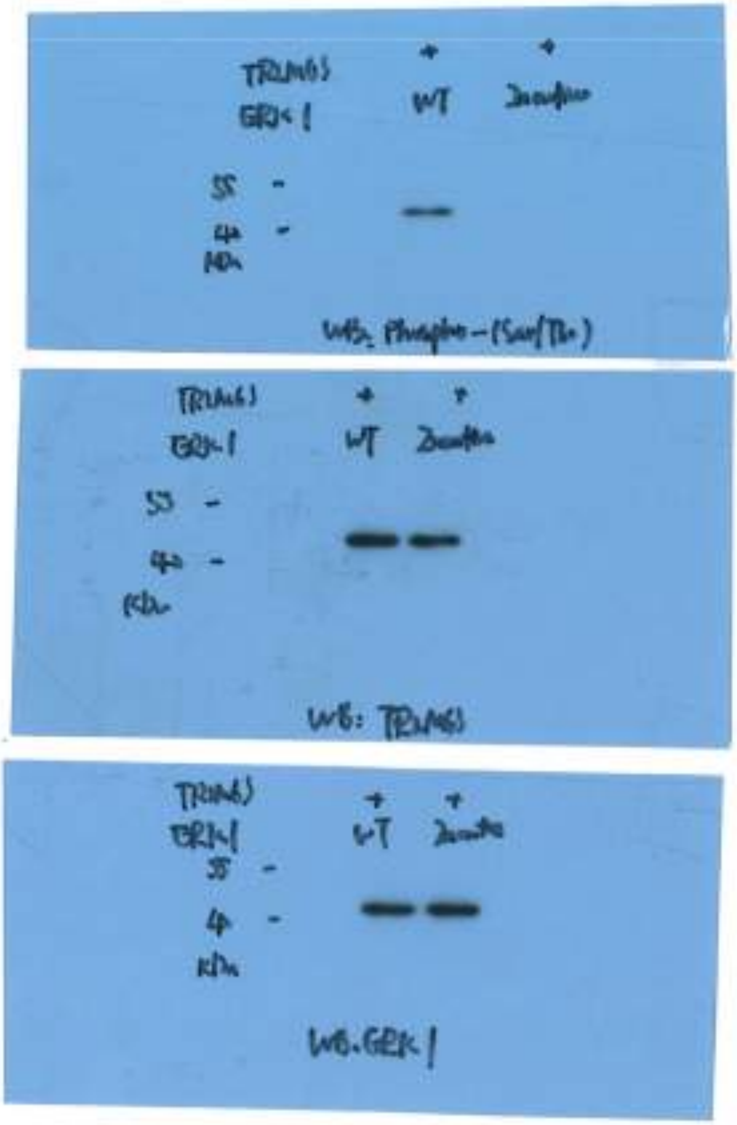

Figure 2D

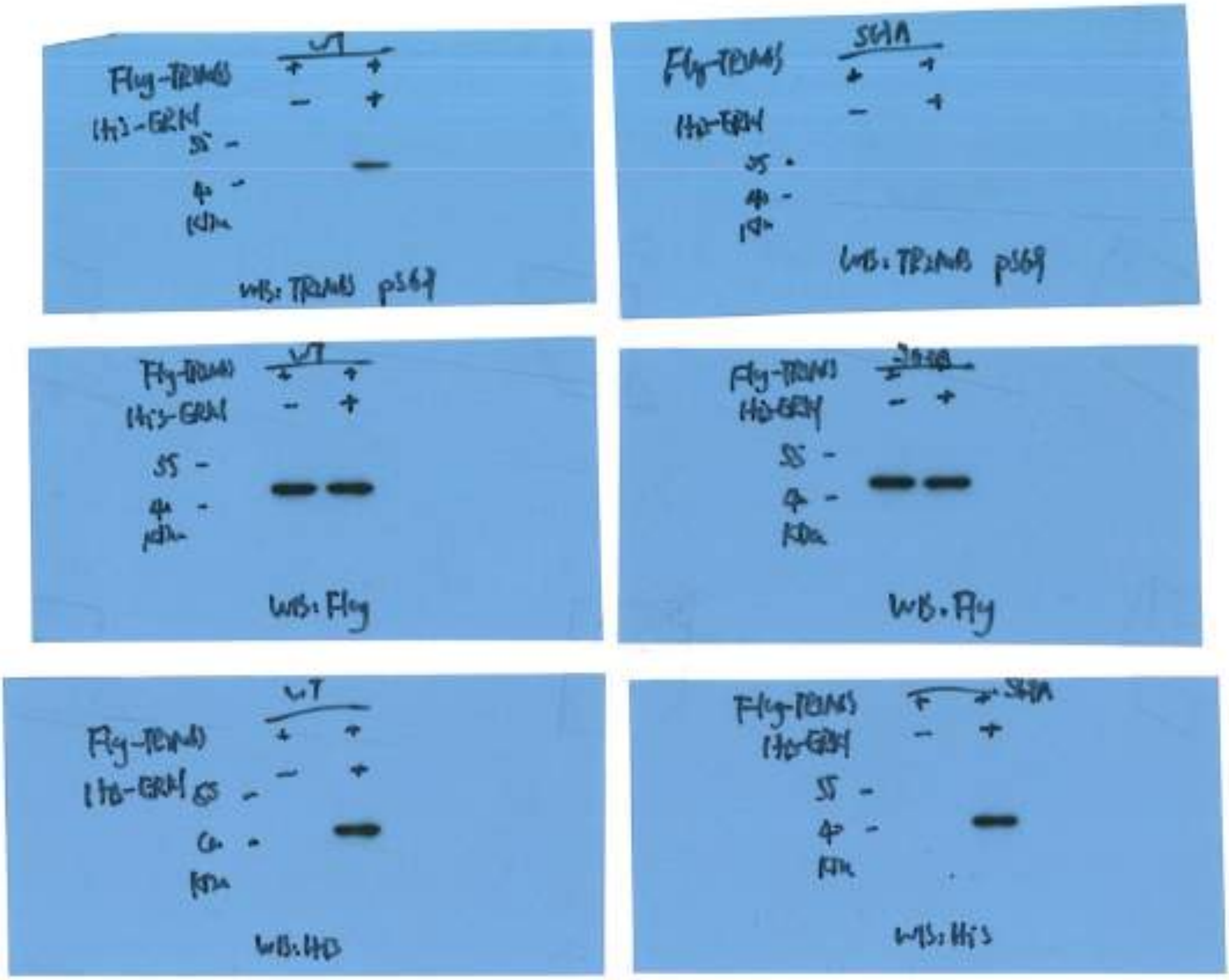

Figure 2G

2H

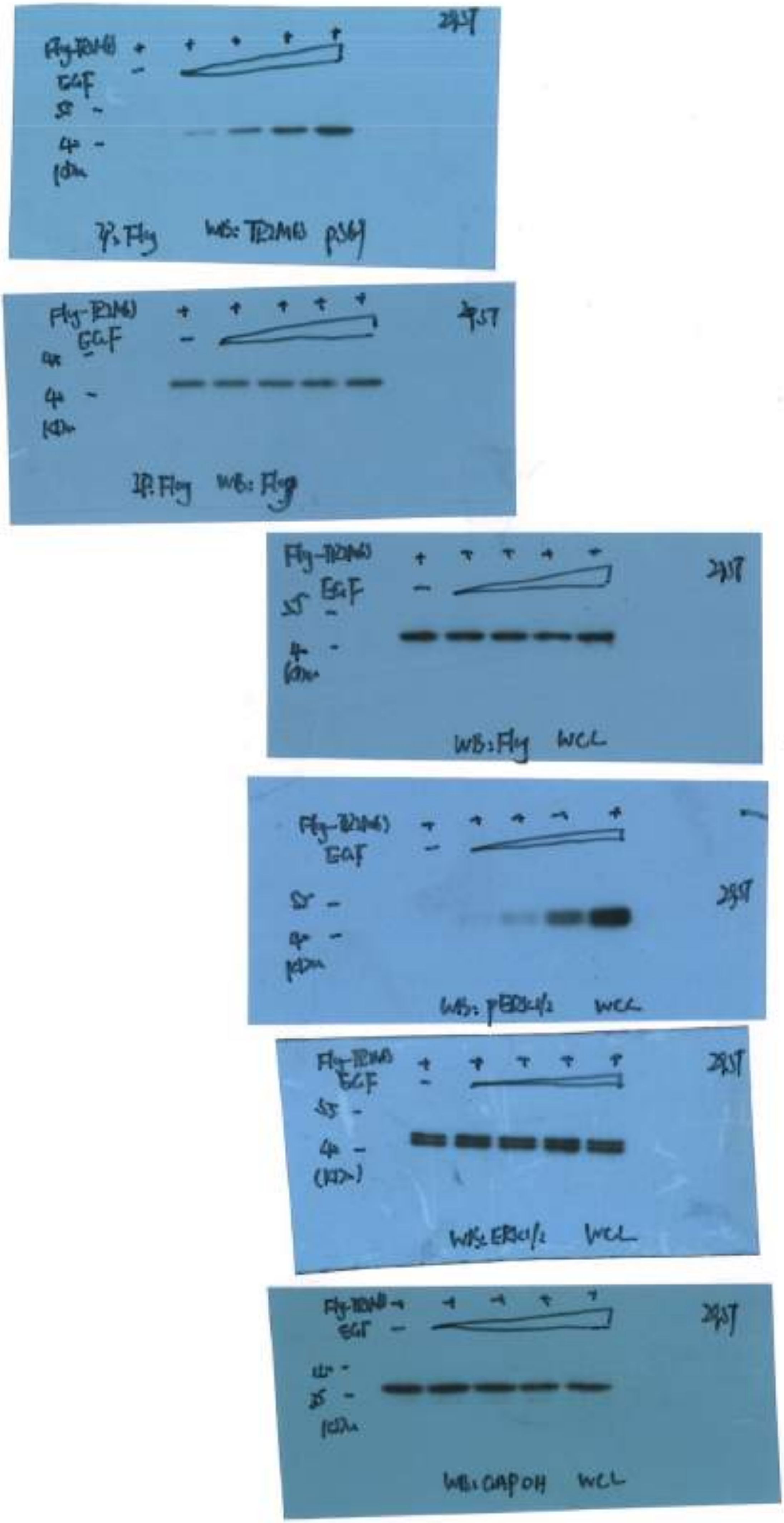

293T
IP: Flag WB: Flag
WB: Flag WCL
WB: pERK1/2 WCL
WB: ERK1/2 WCL
WB: GAPDH WCL

Figure 2H

21

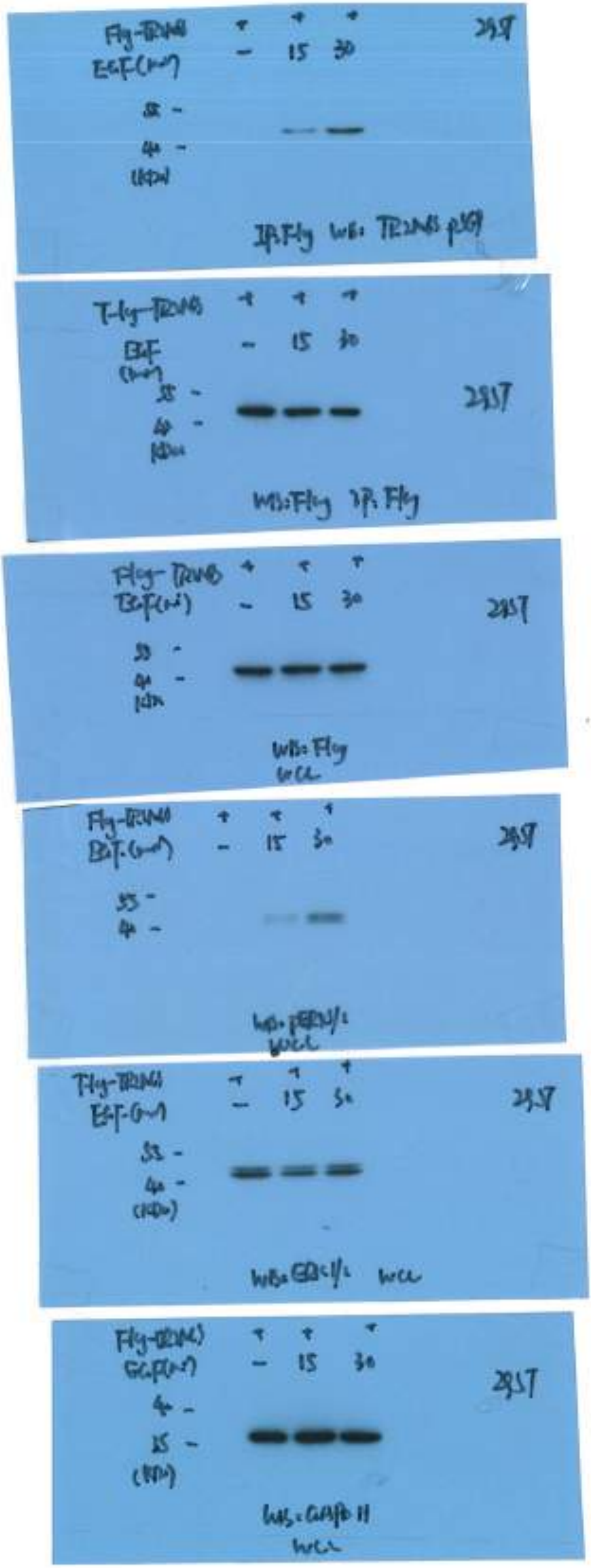

Figure 21

4J

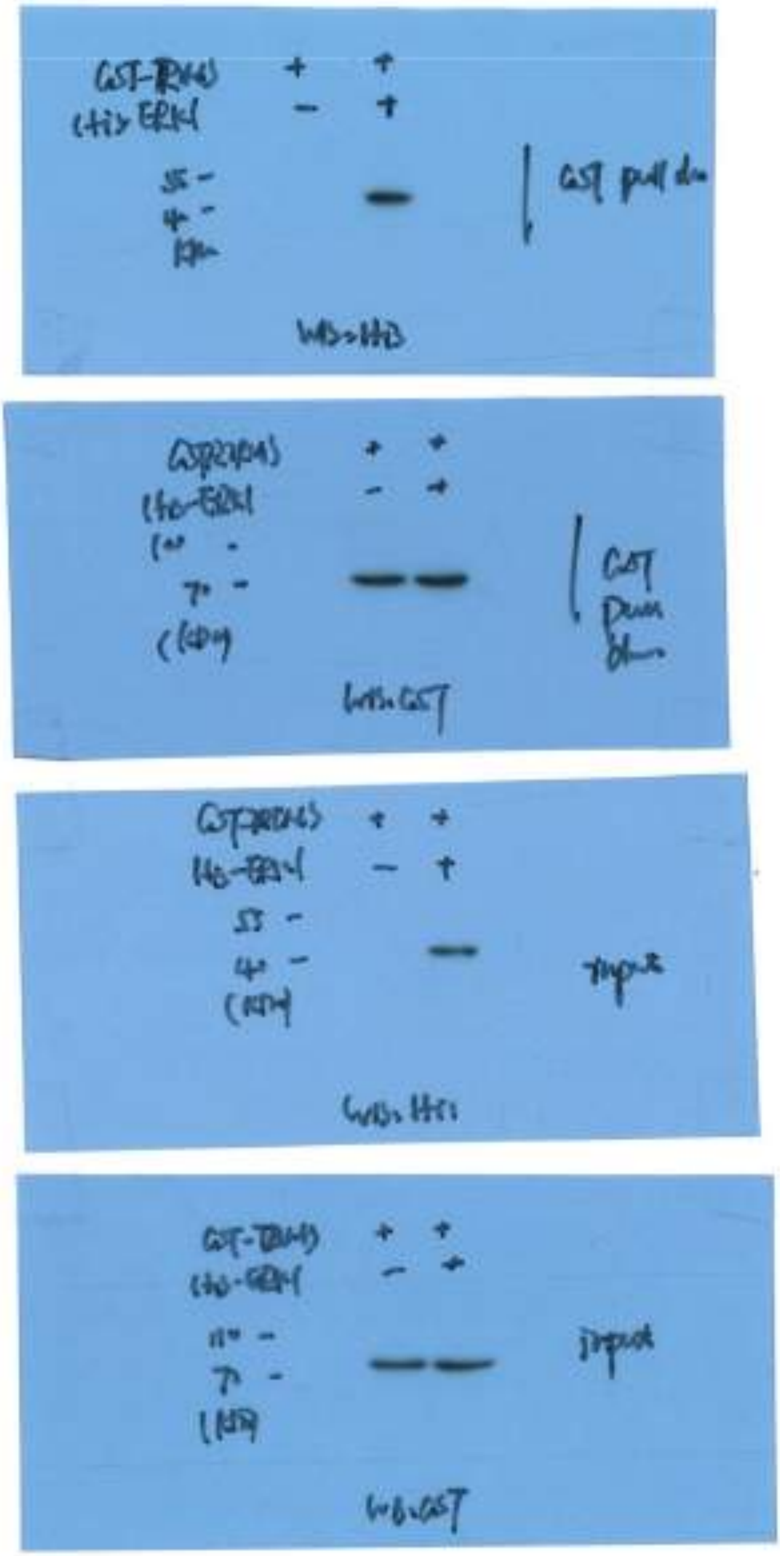

Figure 2J

2K

Figure 2K

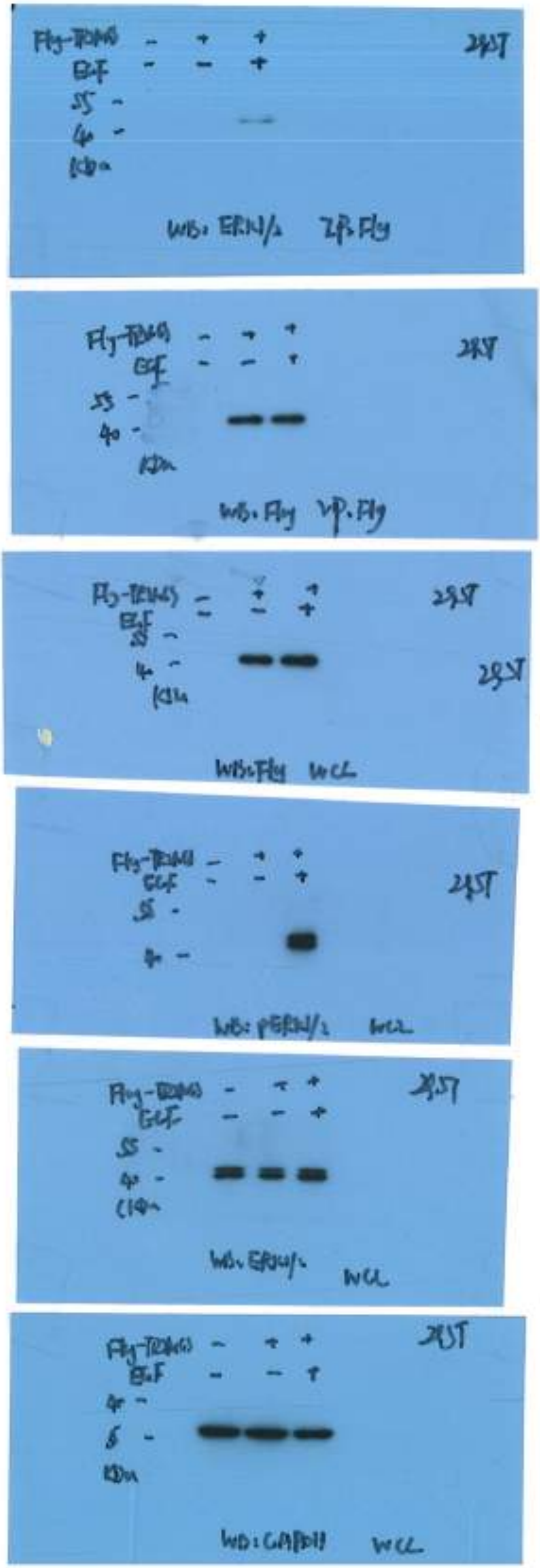
EGF
55
40
KDa
293T
WB: ERK1/2 IP: Flag
WB: Flag IP: Flag
WB: Flag WCL
WB: pERK1/2 WCL
WB: ERK1/2 WCL
WB: GAPDH WCL

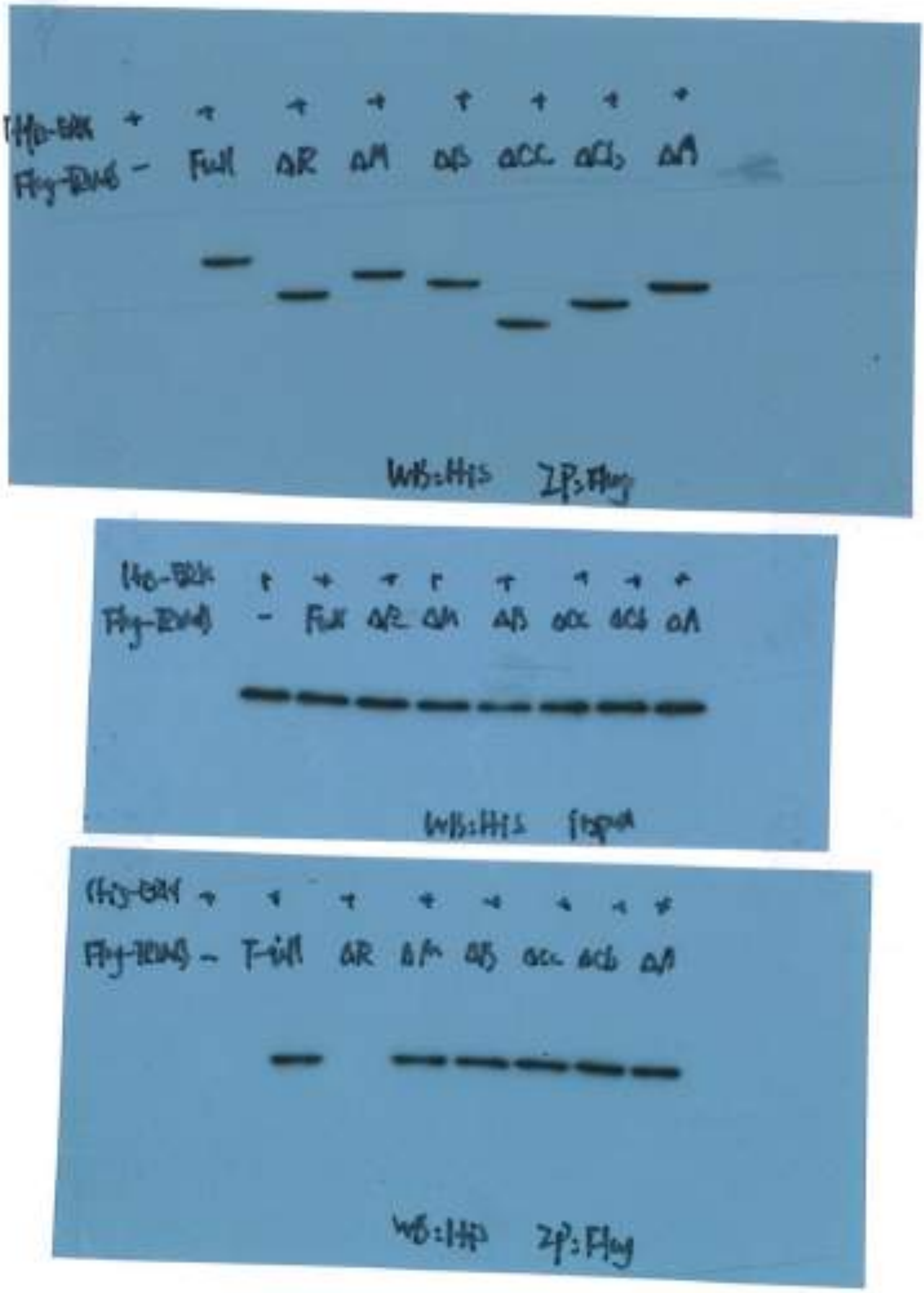

Figure 2M

W

2N

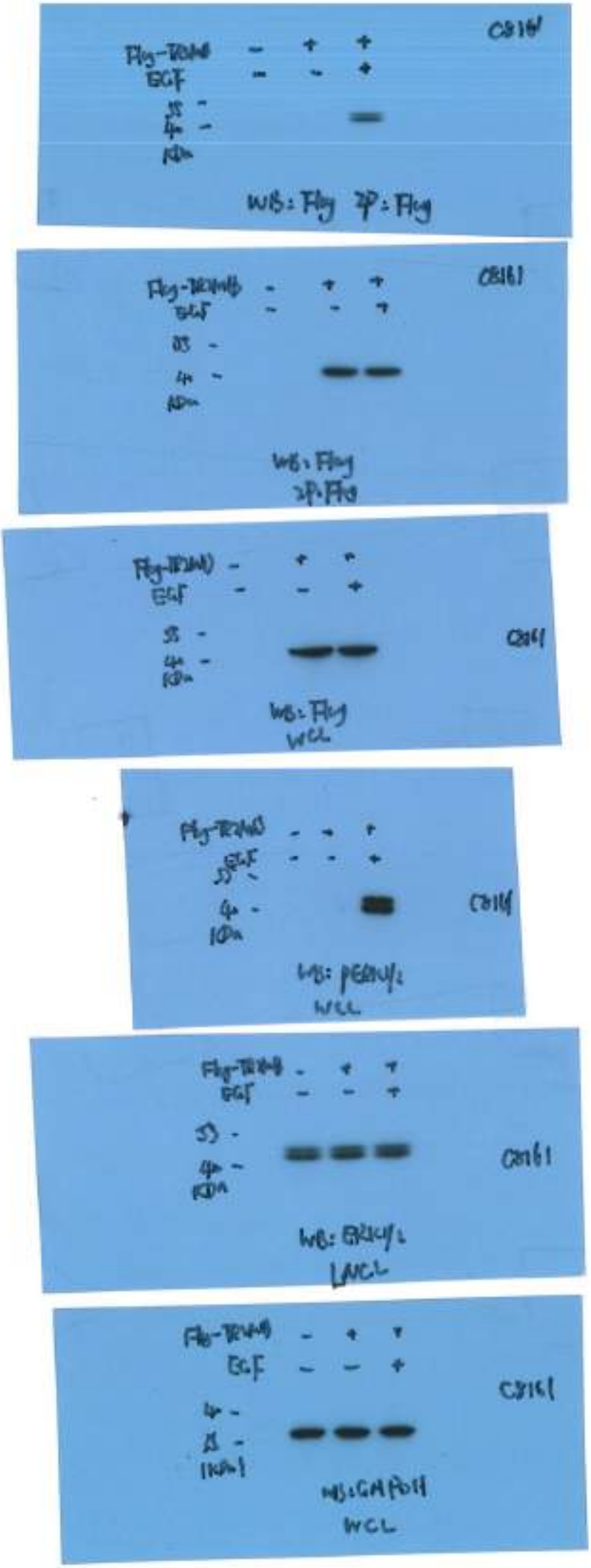

Figure 2N

20

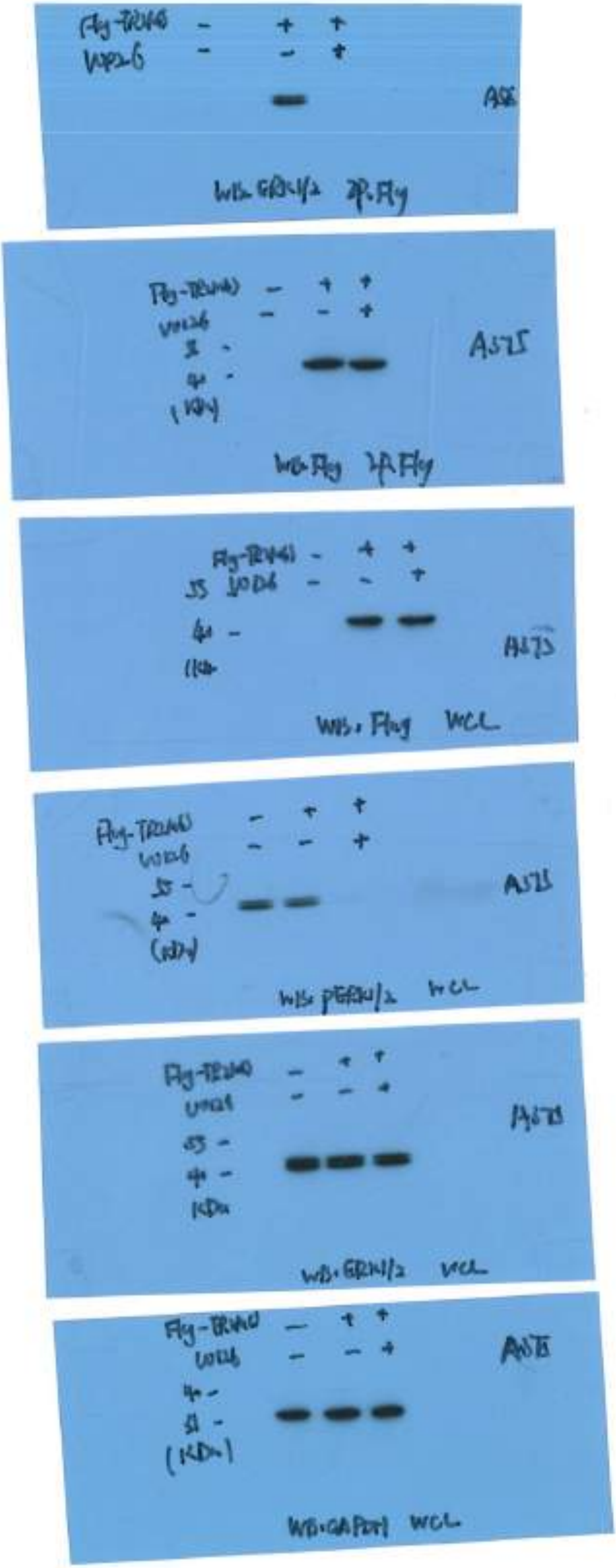

Figure 20

2P

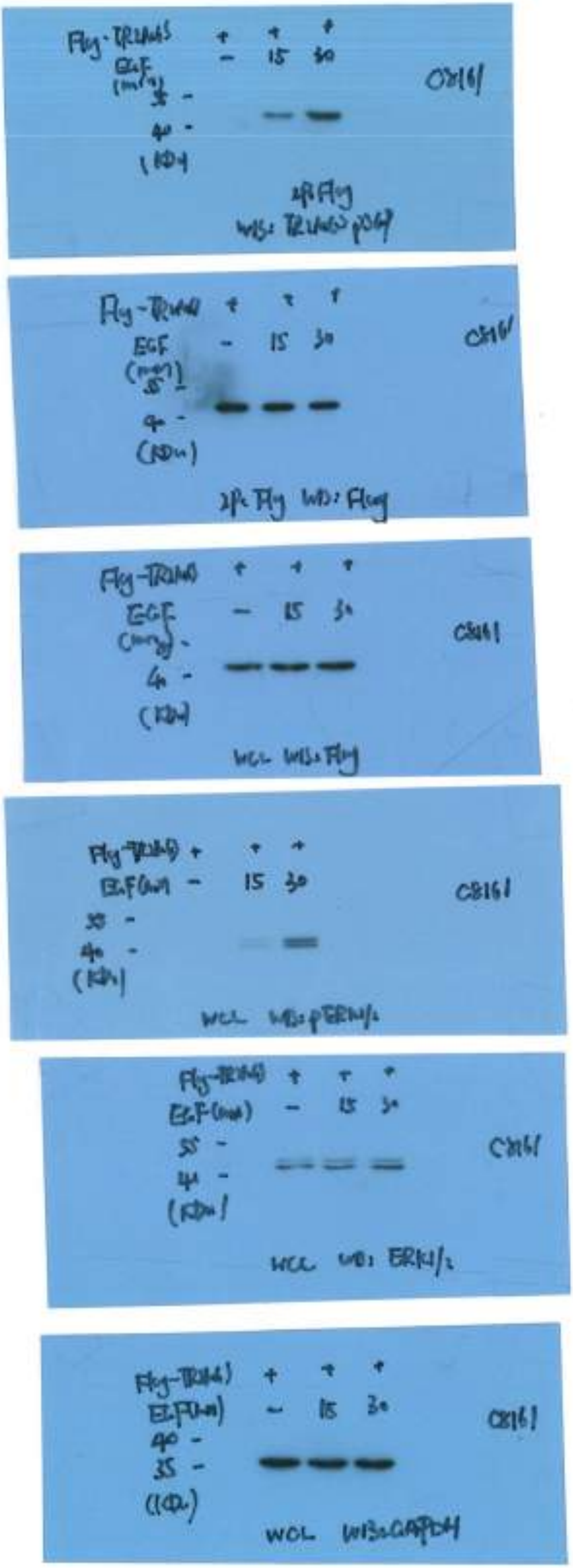

Figure 2P

2Q

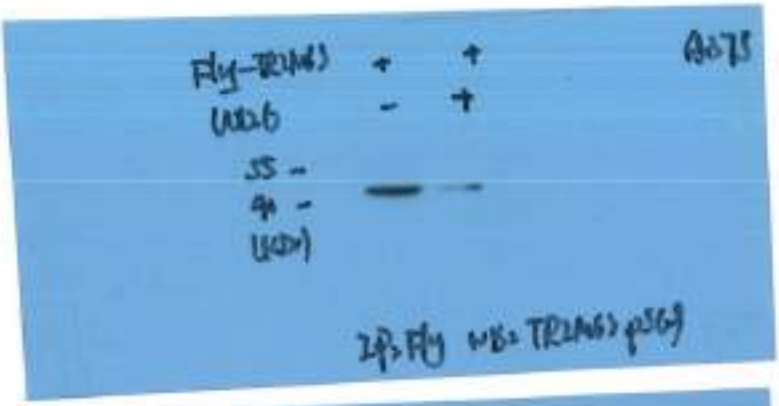
A375
35 -
40 -
(KDa)

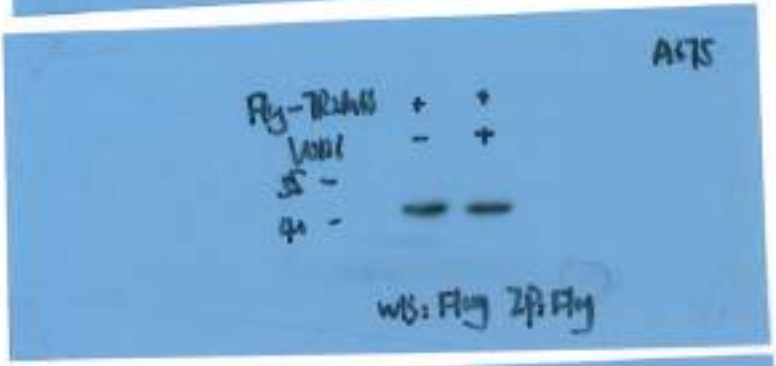
A375
35 -
40 -
WB: Flag IP: Flag

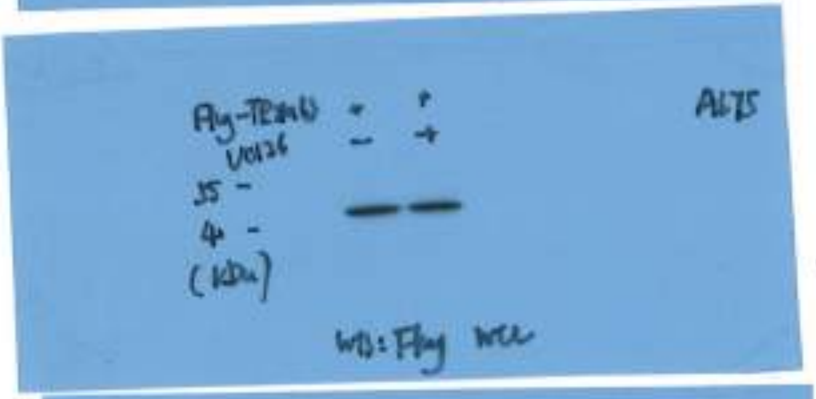
A375
35 -
40 -
(KDa)
WB: Flag WCL

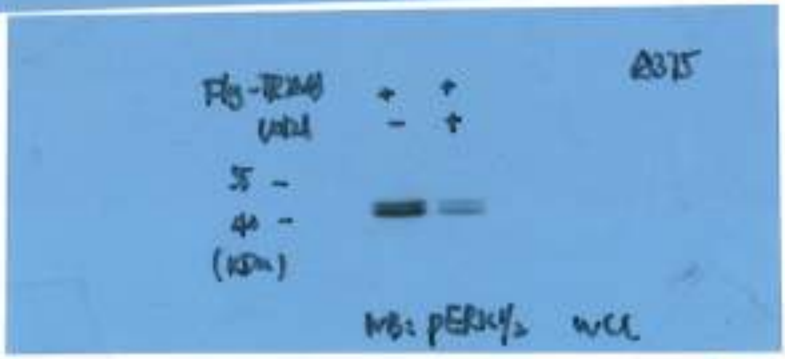
35 -
40 -
(KDa)
WB: pERK1/2 WCL

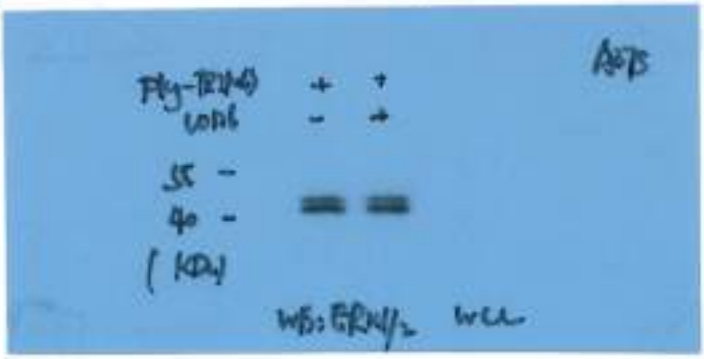
35 -
40 -
(KDa)
WB: ERK1/2 WCL

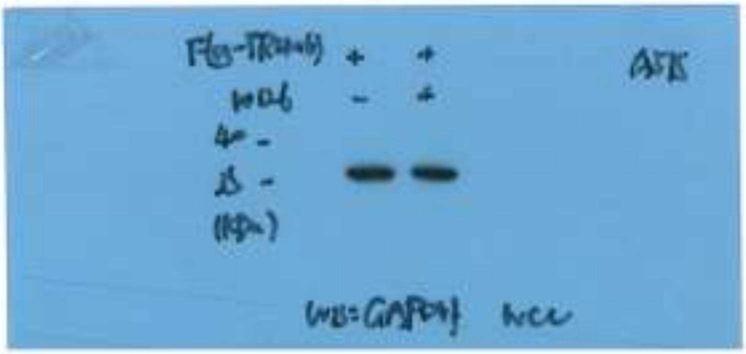
A375
40 -
25 -
(KDa)
WB: GAPDH WCL

Fig 5A

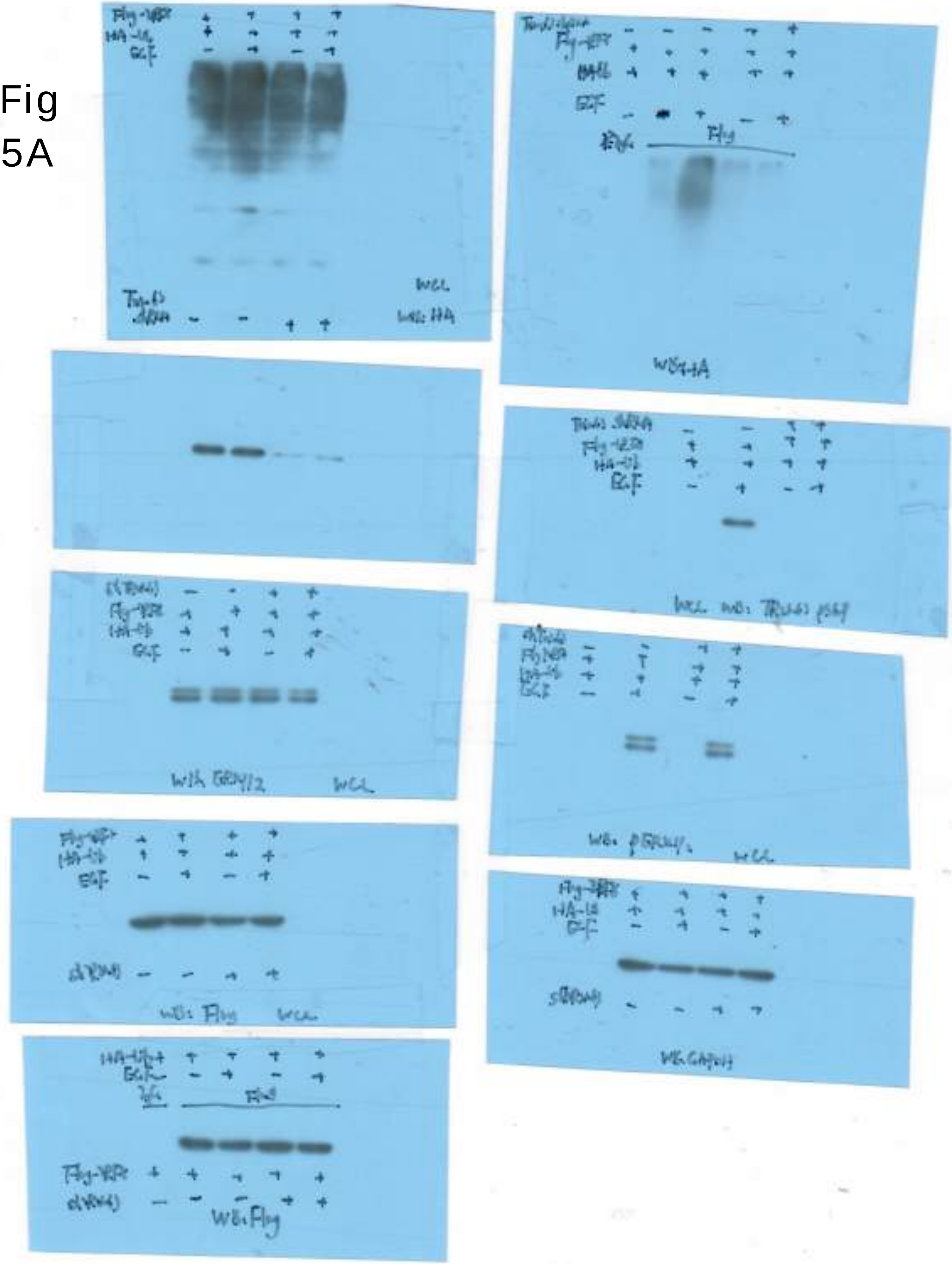

5D

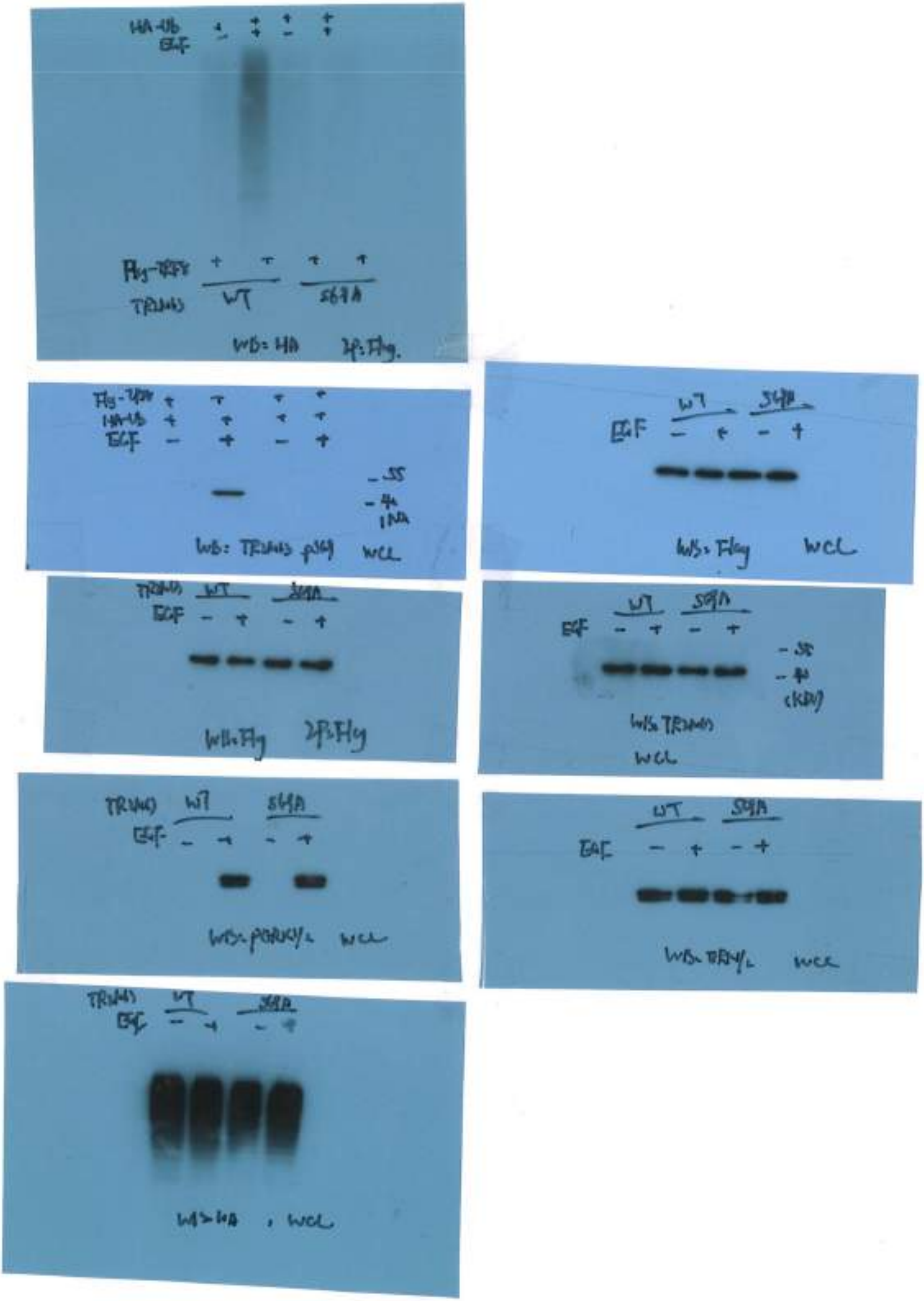

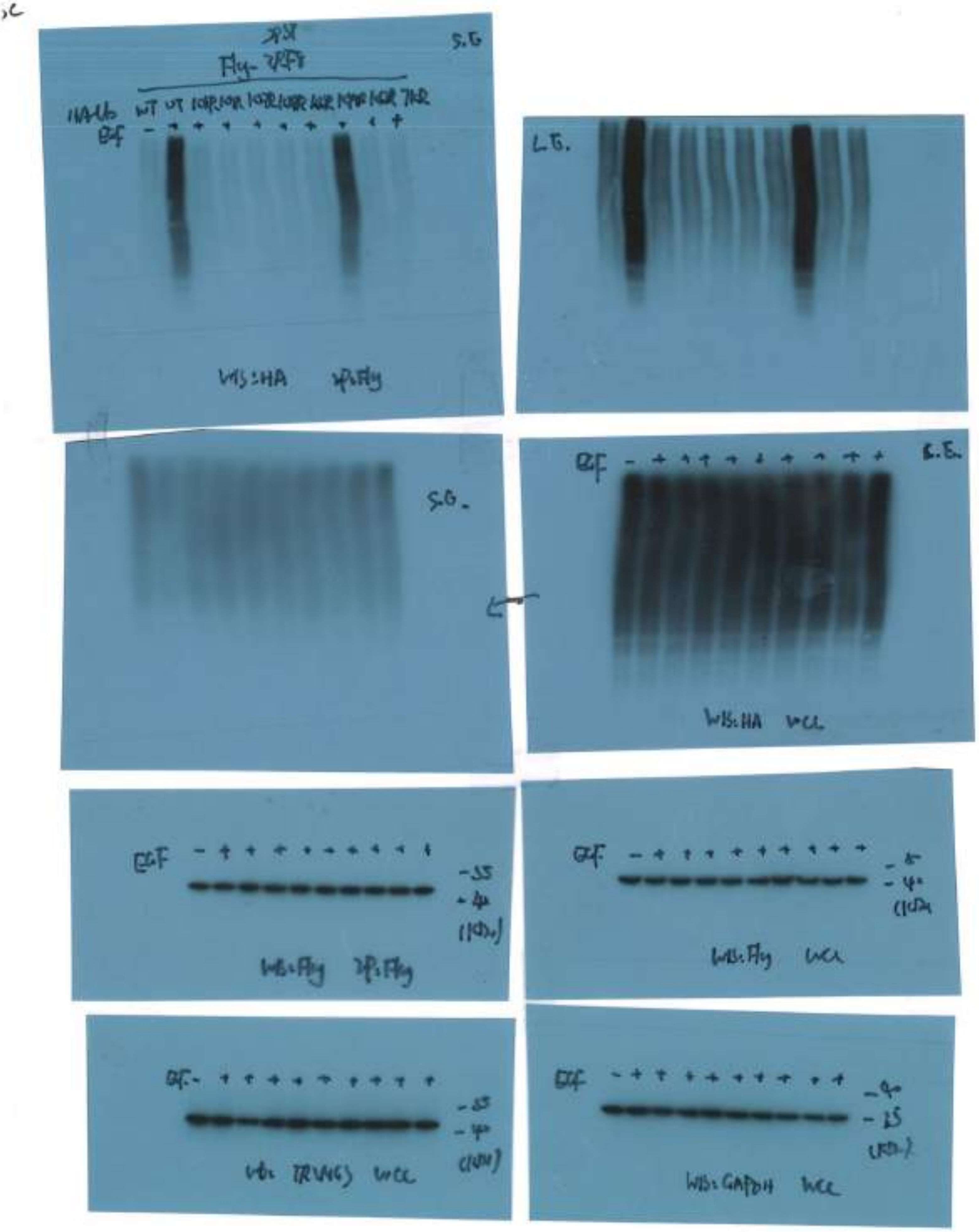
S.G
WT
EGF
L.E.
WB: HA
S.G.
S.E.
WB: HA WCL
(KDa)
GAPDH WCL

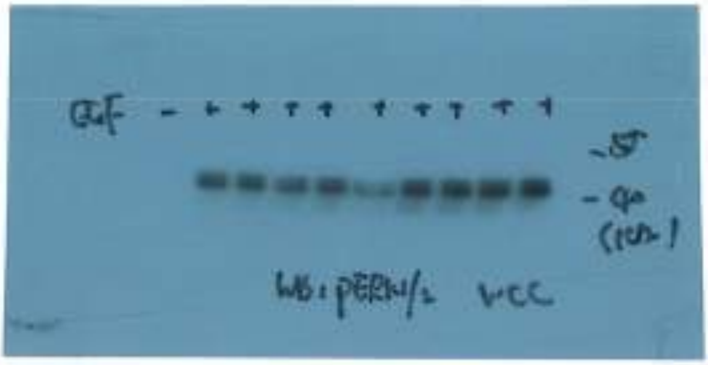
EGF - + + + + + + + + +
55
40
(kDa)
WB: pERK1/2 WCL

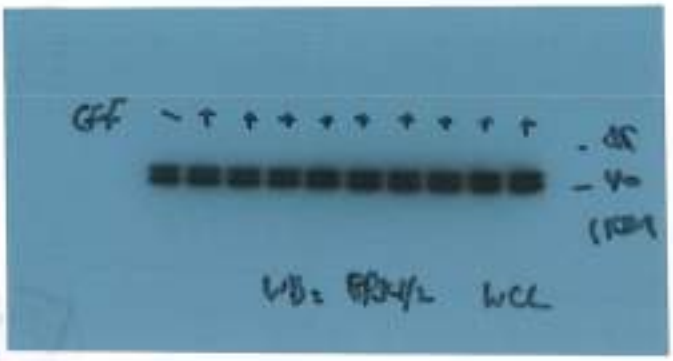
EGF - + + + + + + + + +
55
40
WB: ERK1/2 WCL

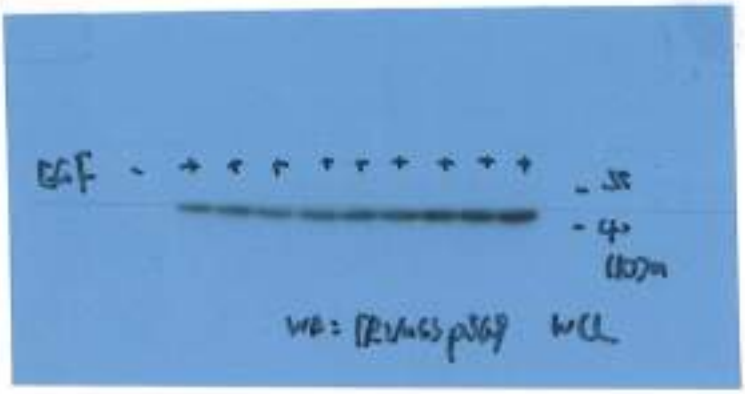
EGF - + + + + + + + + +
55
40
(kDa)

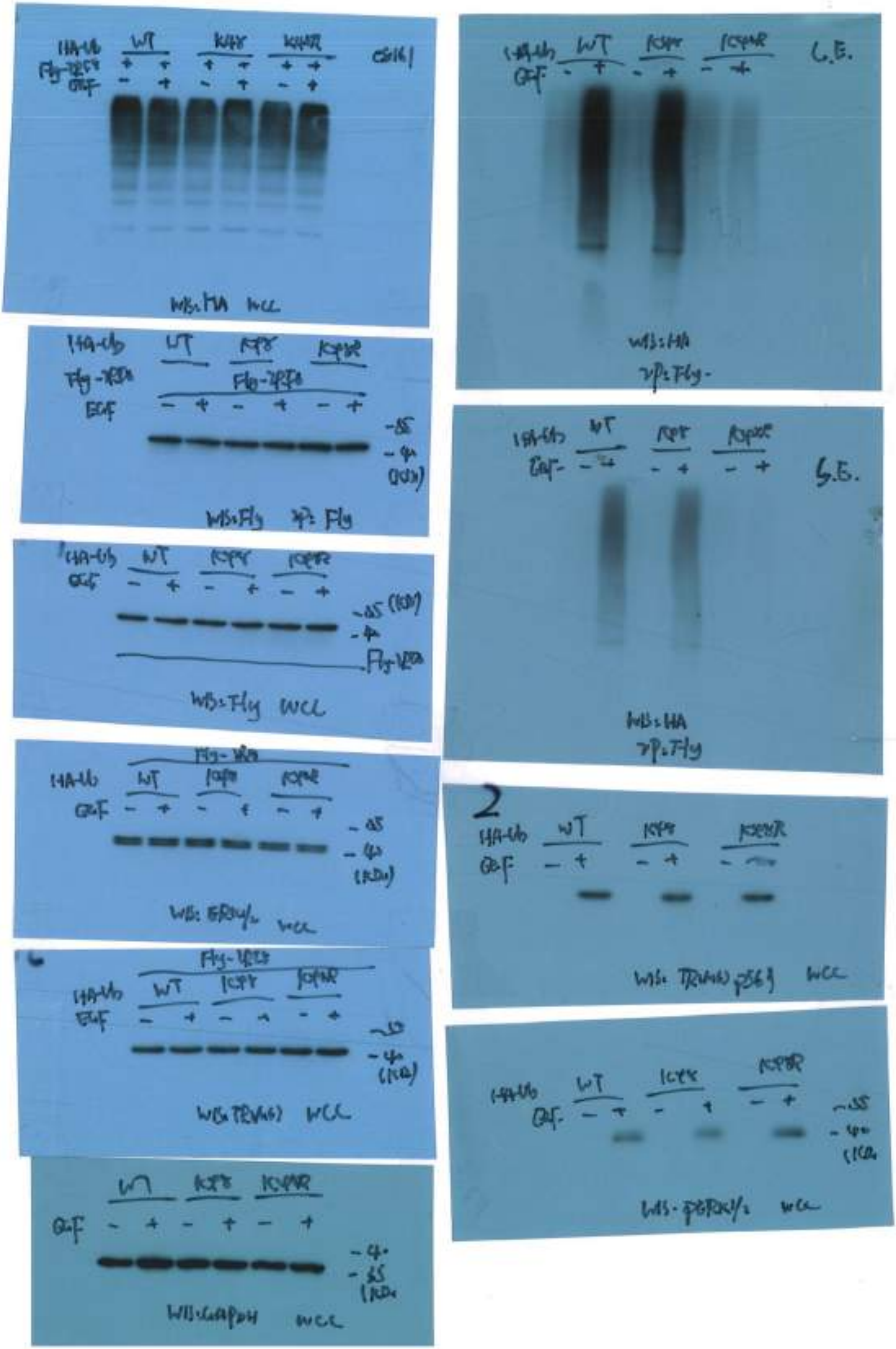

HA-Ub
WT
WB: HA WCL
L.E.
WB: HA
IP: Flag
S.E.
WB: Flag IP: Flag
WB: Flag WCL
WB: GAPDH WCL
2

5F

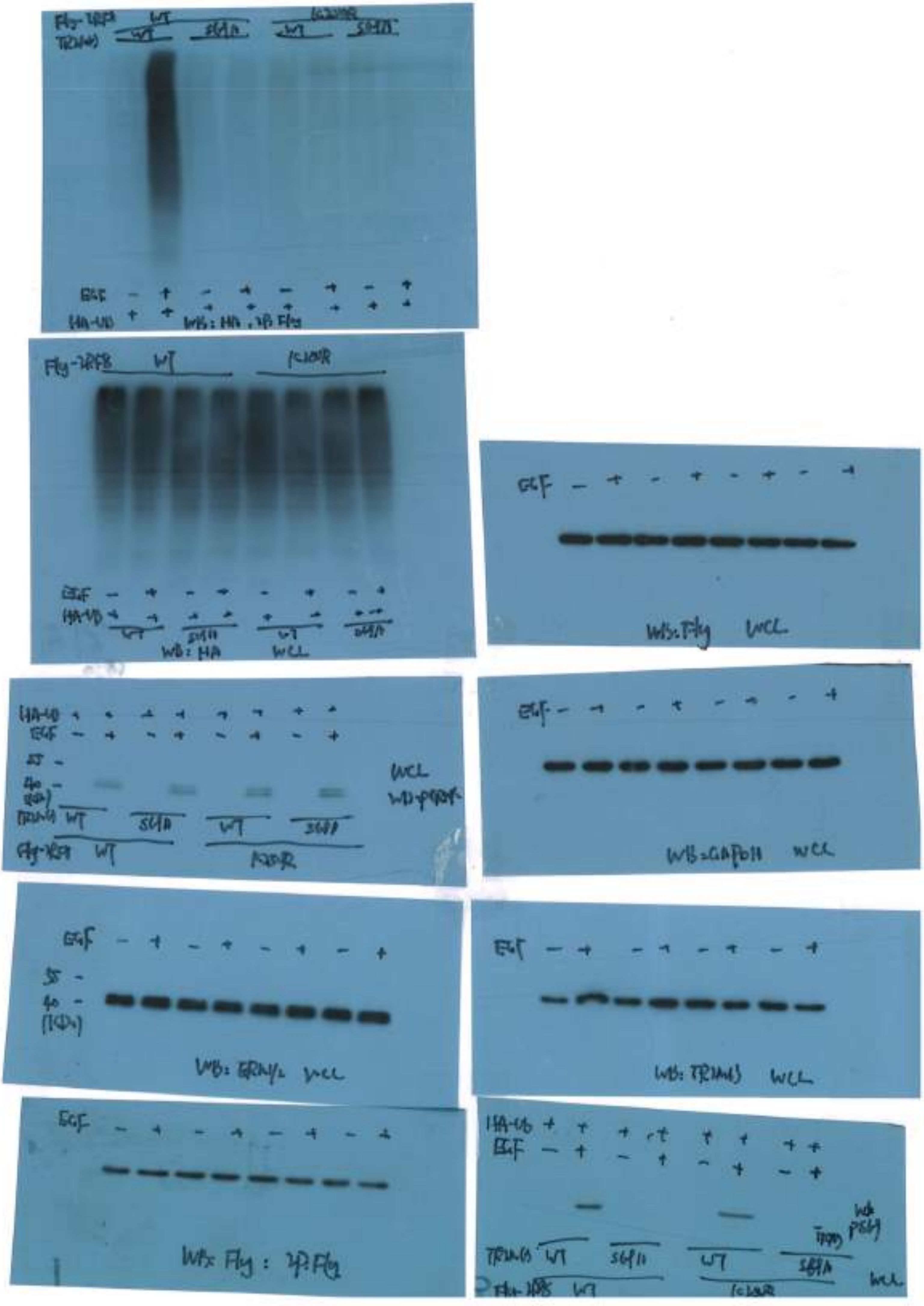

5G

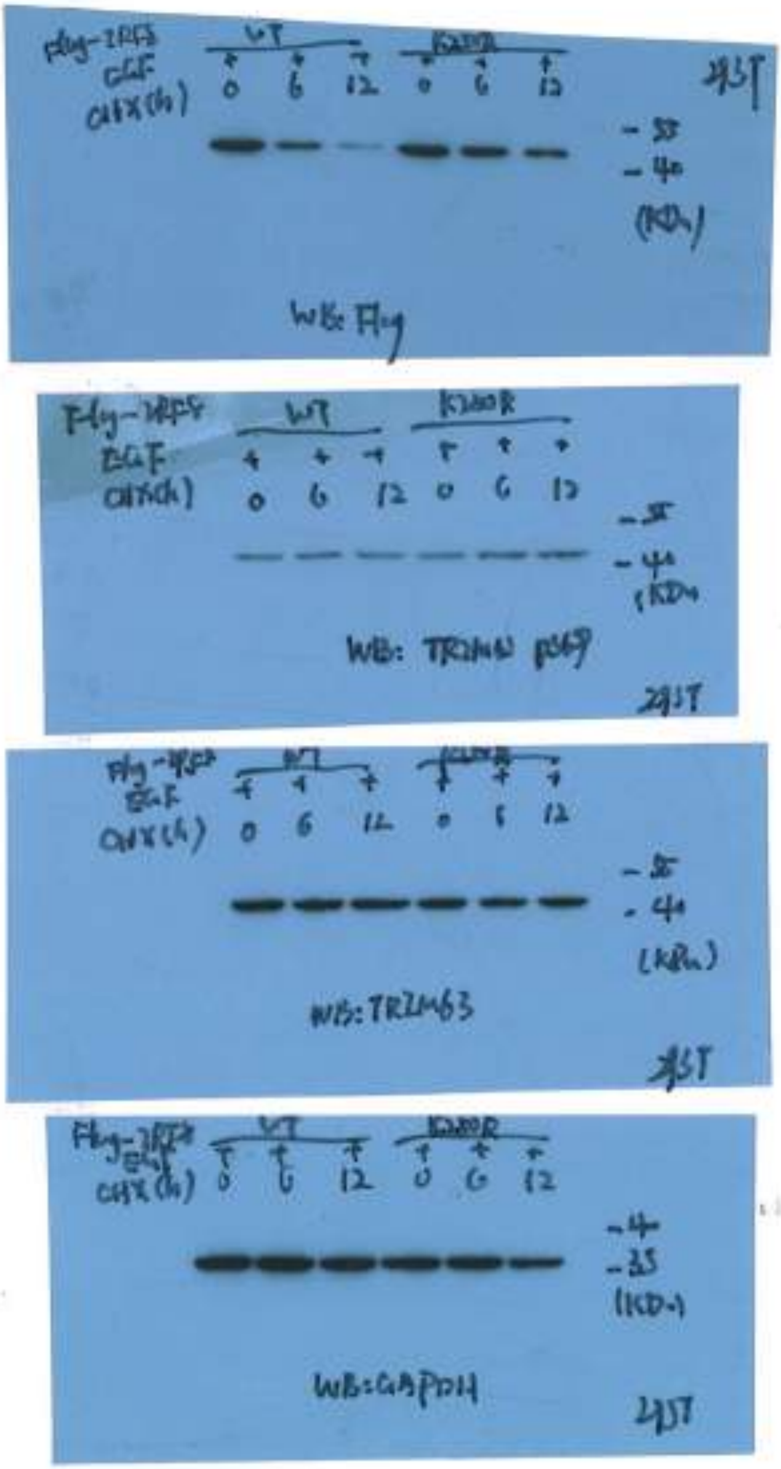

514

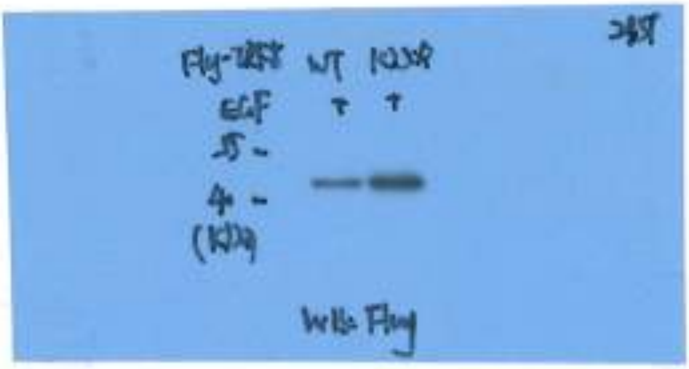
WT
EGF + +
55 -
40 -
WB: Flag

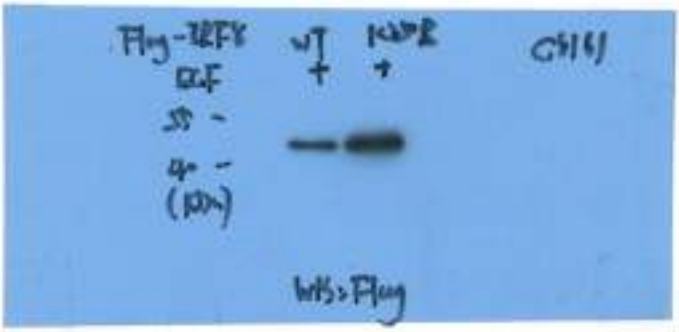
WT
EGF + +
55 -
40 -
WB: Flag

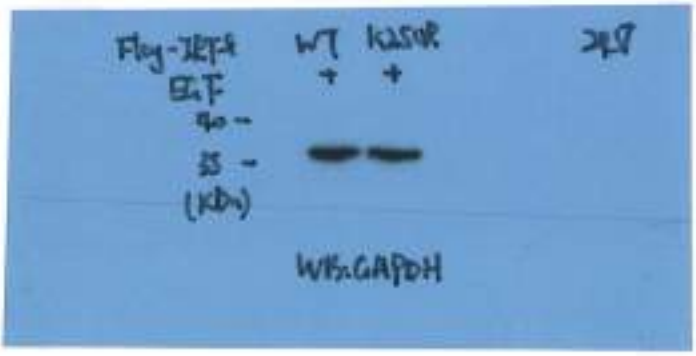
WT K250R
EGF + +
35 -
WB: GAPDH

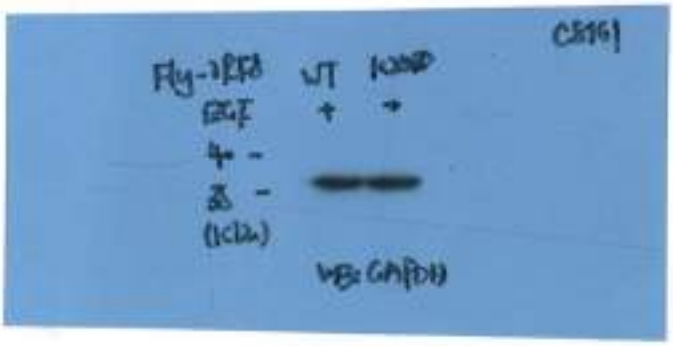
WT
EGF + +
40 -
35 -
WB: GAPDH

S1A

Fig. S1A

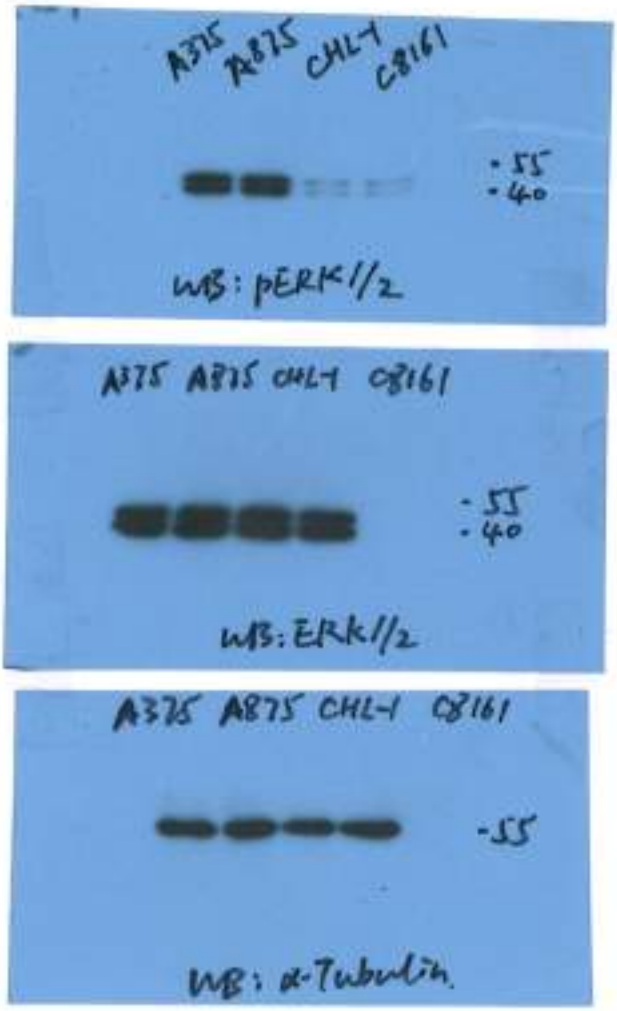
A375 A875 CHL-1 C8161
·55
·40
WB: pERK1/2
A375 A875 CHL-1 C8161
·55
·40
WB: ERK1/2
A375 A875 CHL-1 C8161
-55
WB: α-Tubulin

Fig S1G

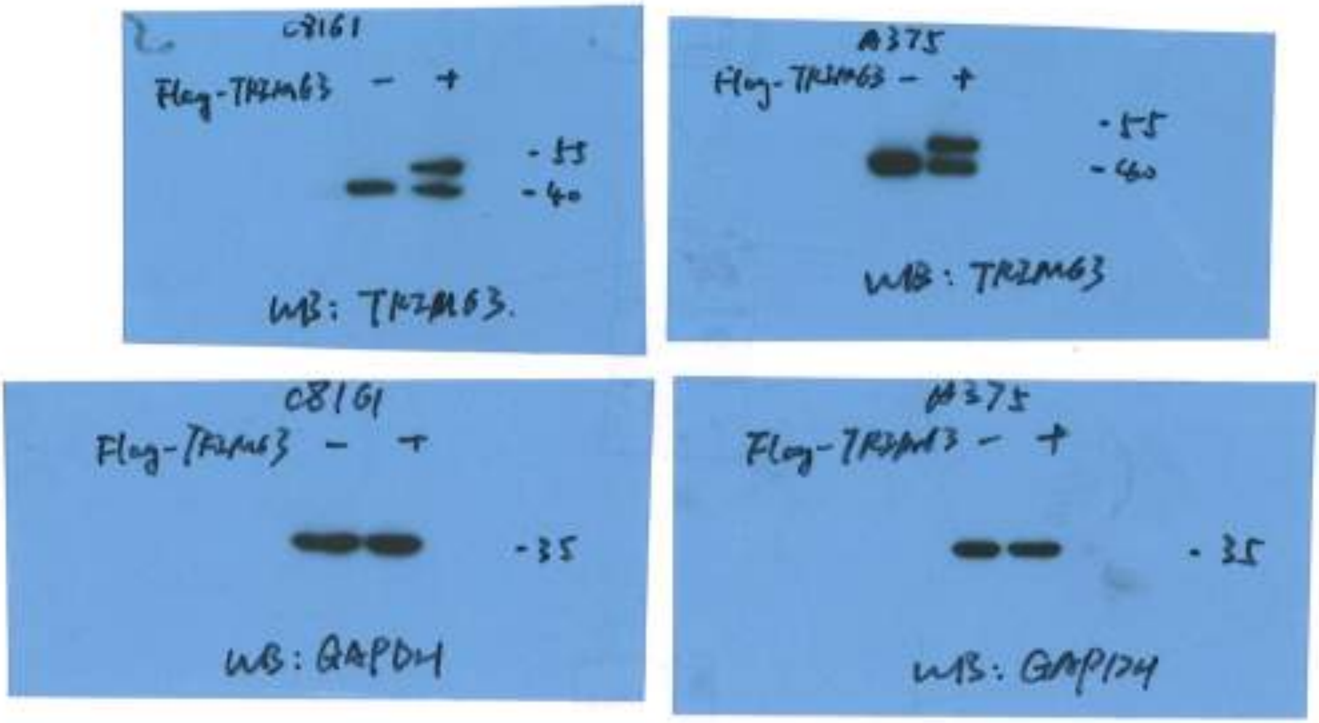
C8161
Flag-TRIM63 - +
- 55
- 40
WB: TRIM63.
A375
Flag-TRIM63 - +
- 55
- 40
WB: TRIM63
C8161
Flag-TRIM63 - +
- 35
WB: GAPDH
A375
Flag-TRIM63 - +
- 35
WB: GAPDH

Fig S1B-E

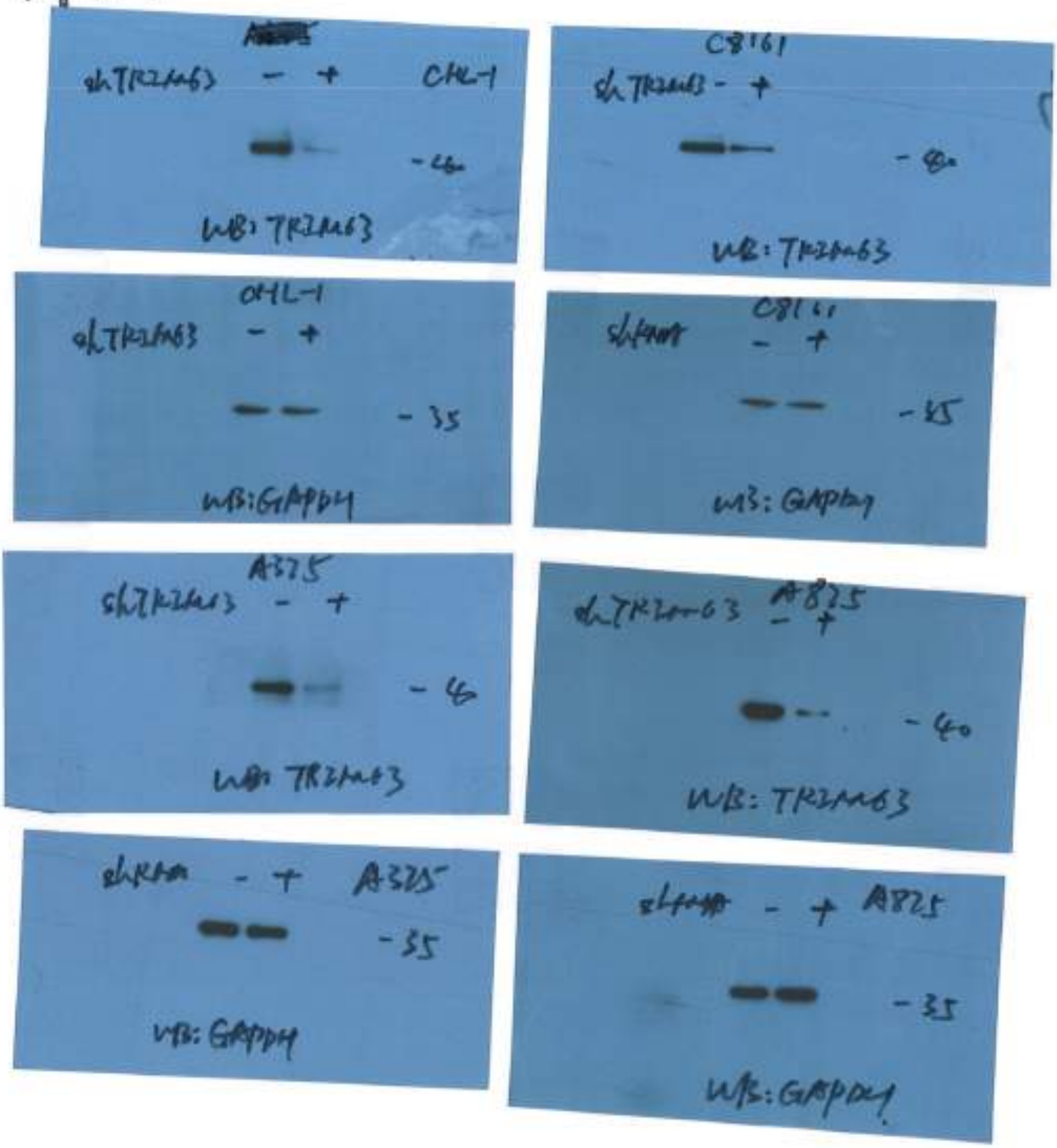

S2A

Fig S2A

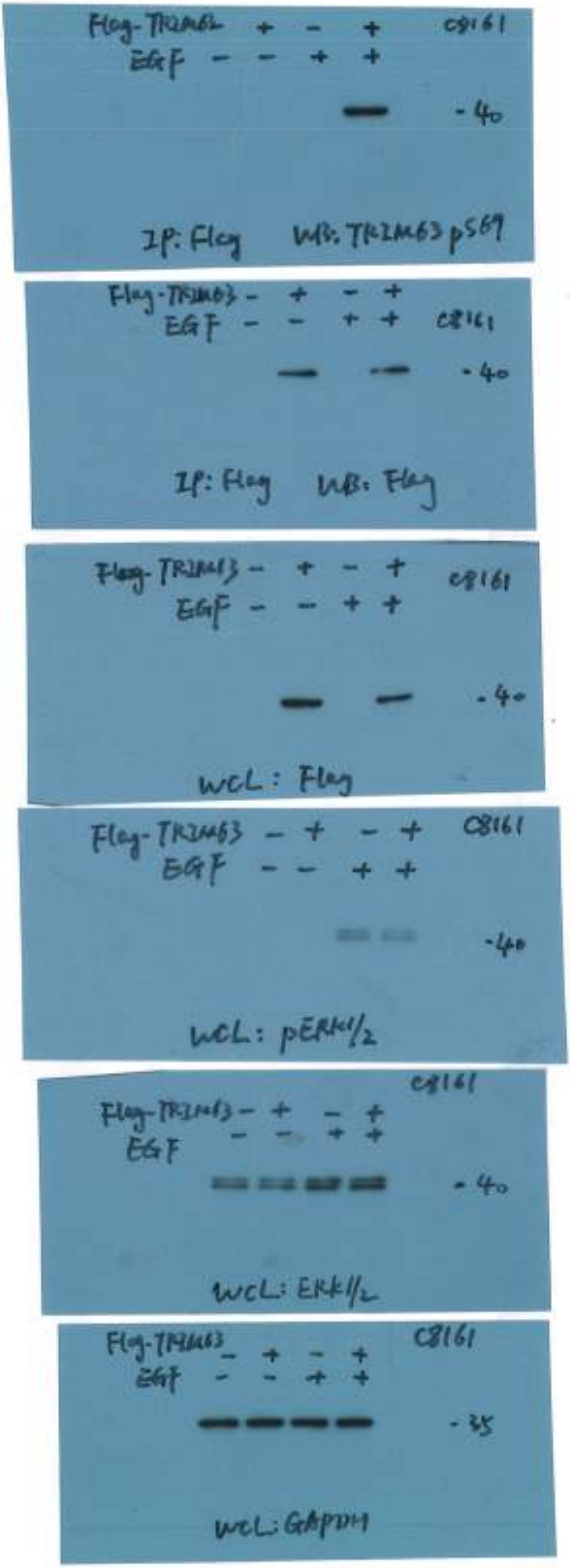
Flag-TRIM63 + − +
EGF − − + +
C8161
-40
IP: Flag WB: TRIM63 pS69
Flag-TRIM63 − + − +
EGF − − + +
C8161
-40
IP: Flag WB: Flag
Flag-TRIM63 − + − +
EGF − − + +
C8161
-40
WCL: Flag
Flag-TRIM63 − + − +
EGF − − + +
C8161
-40
WCL: pERK1/2
C8161
Flag-TRIM63 − + − +
EGF − − + +
-40
WCL: ERK1/2
Flag-TRIM63 − + − +
EGF − − + +
C8161
-35
WCL: GAPDH

S2C

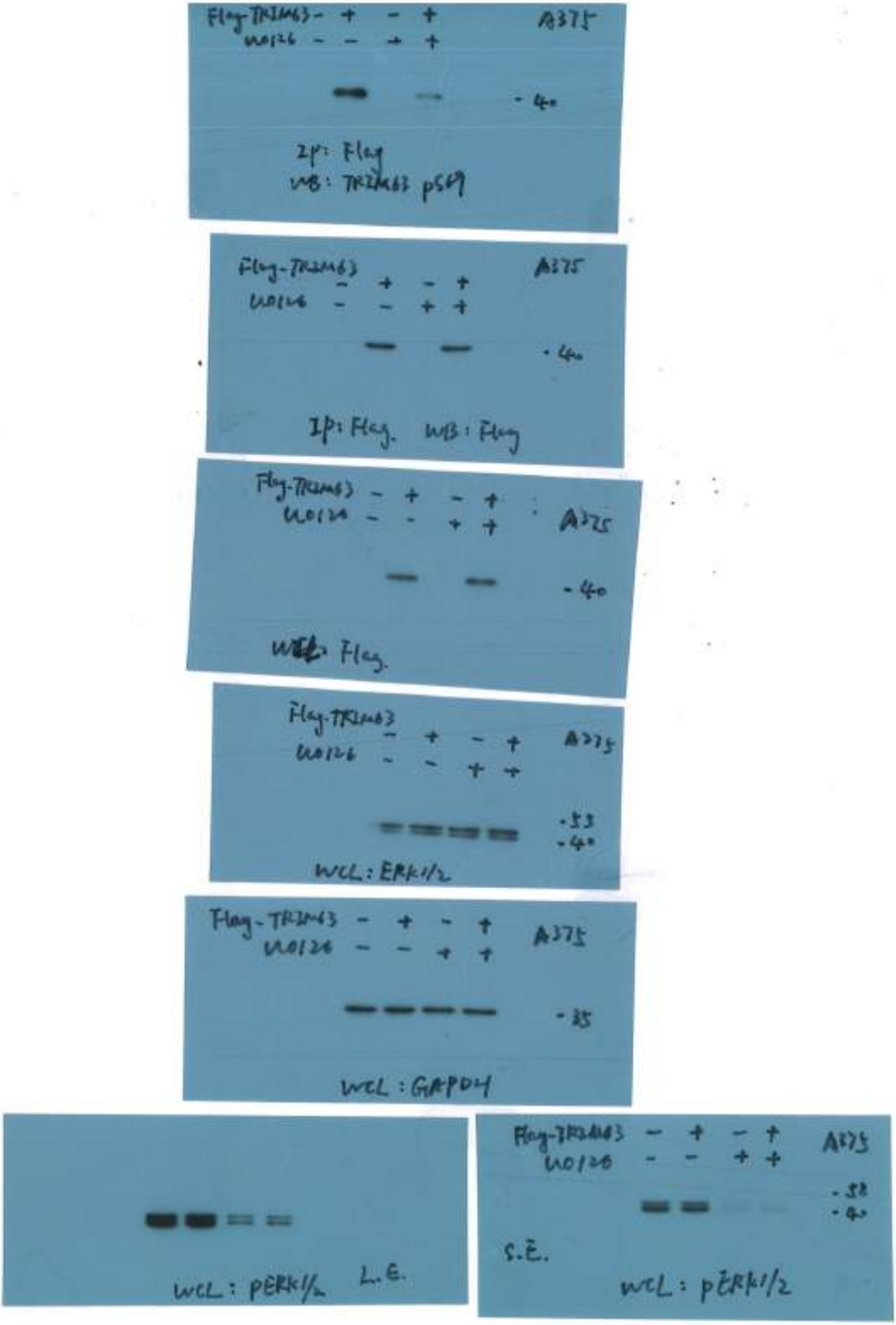
Flag-TRIM63 - + - +
W0126 - - + +
A375
40
IP: Flag
WB: TRIM63 pS?9
Flag-TRIM63 - + - +
W0126 - - + +
A375
40
IP: Flag. WB: Flag
Flag-TRIM63 - + - +
W0126 - - + +
A375
40
WB: Flag.
Flag-TRIM63 - + - +
W0126 - - + +
A375
53
40
WCL: ERK1/2
Flag-TRIM63 - + - +
W0126 - - + +
A375
35
WCL: GAPDH
WCL: pERK1/2 L.E.
Flag-TRIM63 - + - +
W0126 - - + +
A375
58
40
S.E.
WCL: pERK1/2

S2E

Fig. S2-E

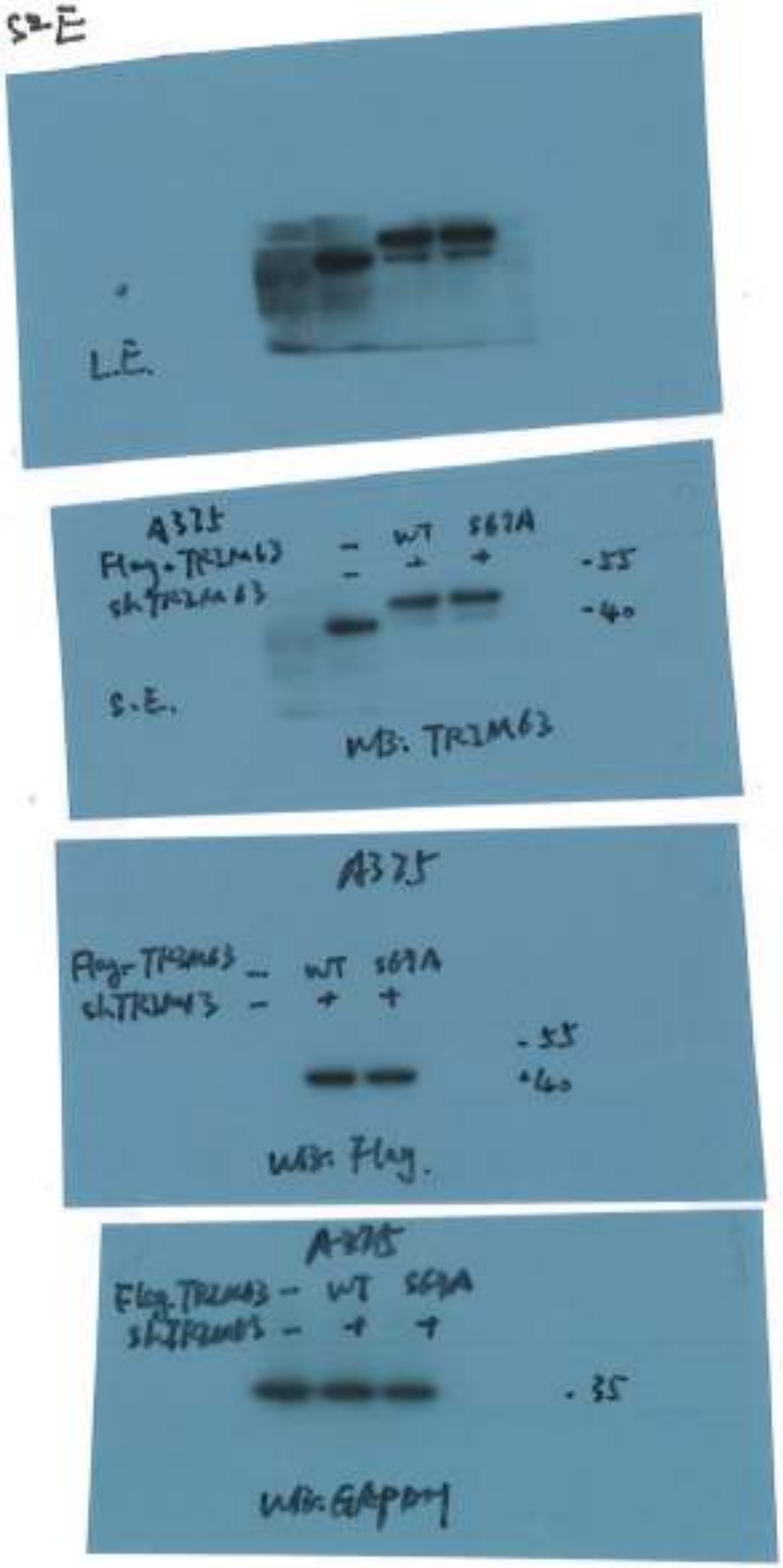

L.E.
A375
Flag-TRIM63 – WT S67A
shTRIM63 – + +
-55
-40
S.E.
WB: TRIM63
A375
Flag-TRIM63 – WT S67A
shTRIM63 – + +
-55
-40
WB: Flag.
A375
Flag-TRIM63 – WT S67A
shTRIM63 – + +
-35
WB: GAPDH

S2G

Fig. S2G

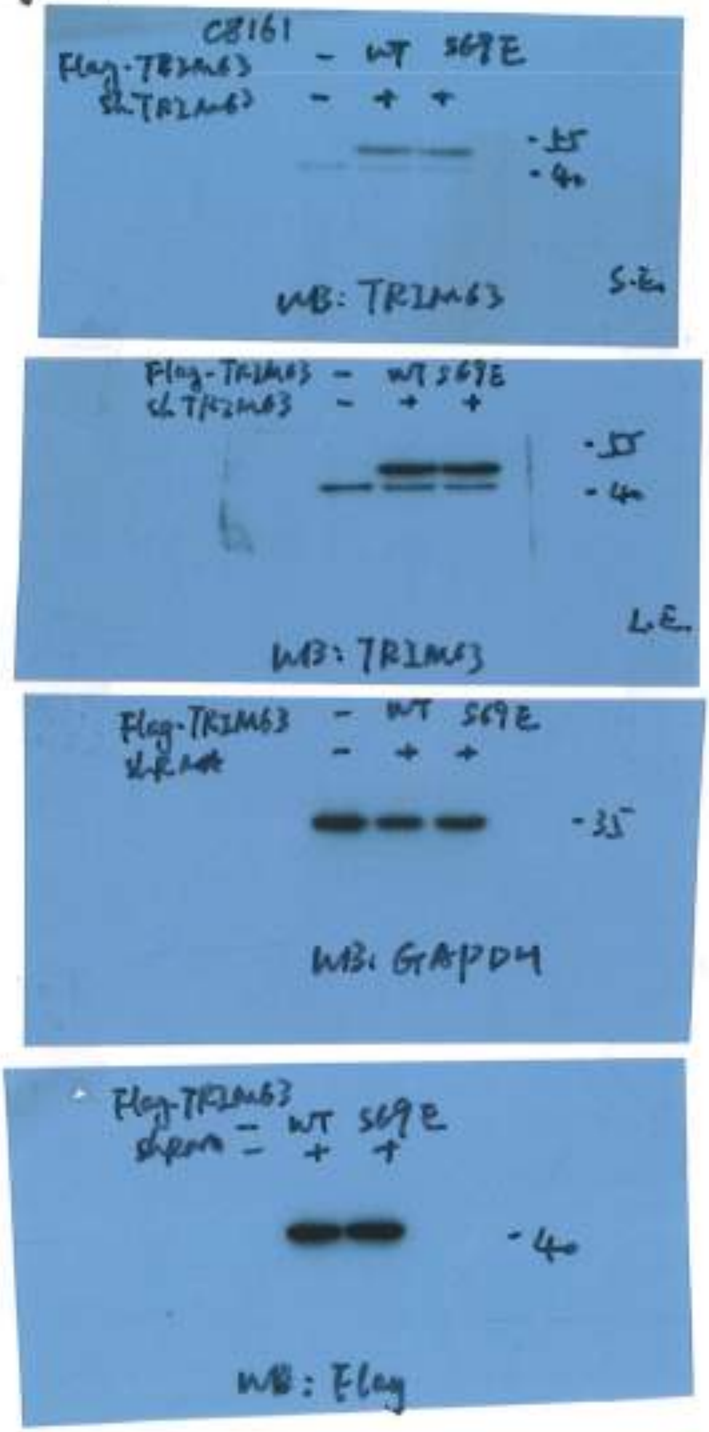
C8161
Flag-TRIM63 - WT S69E
sh.TRIM63 - + +
-55
-40
WB: TRIM63
S.E.
Flag-TRIM63 - WT S69E
sh.TRIM63 - + +
-55
-40
L.E.
WB: TRIM63
Flag-TRIM63 - WT S69E
- + +
-35
WB: GAPDH
Flag-TRIM63 - WT S69E
- + +
-40
WB: Flag

S3C and I

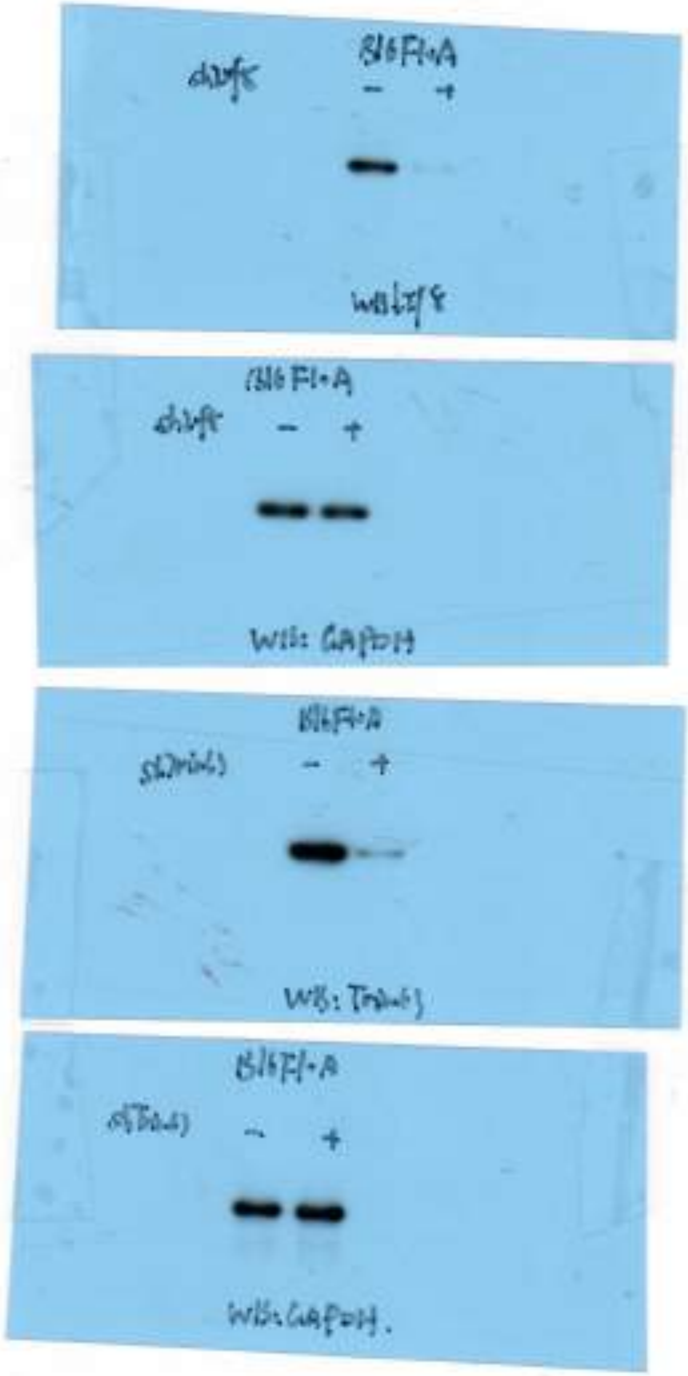

4c

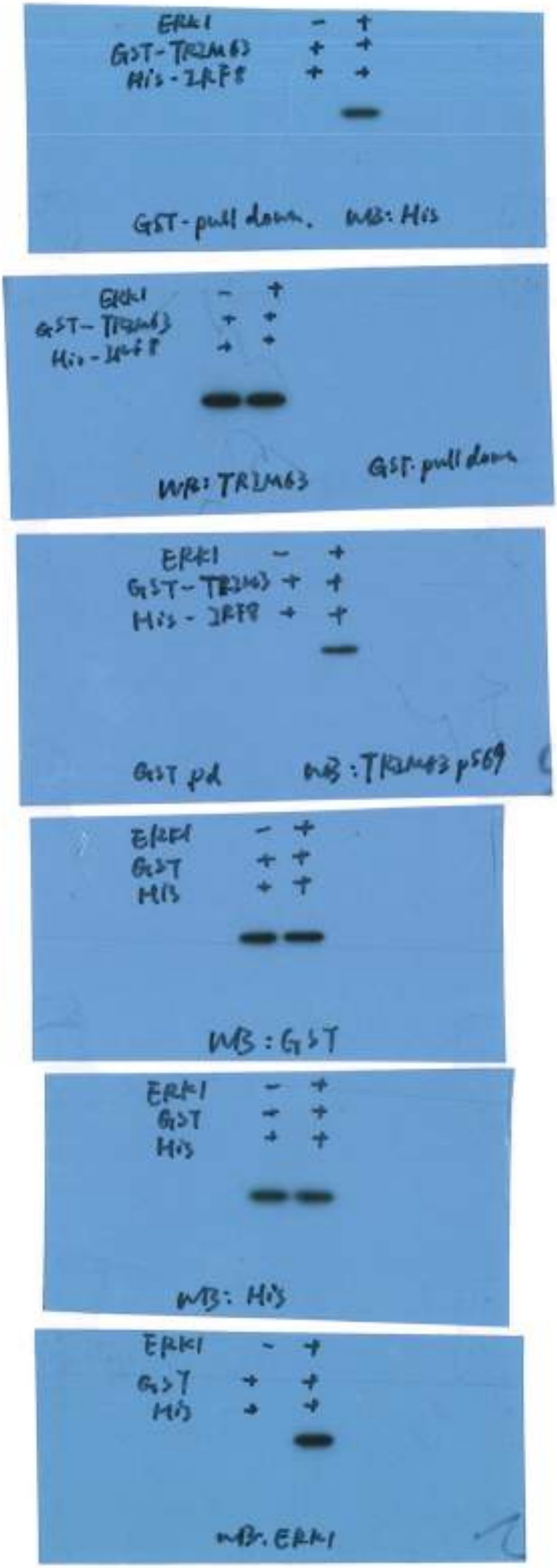

ERK1
GST-TRIM63
His-IRF8
GST-pull down. WB: His
ERK1
GST-TRIM63
His-IRF8
WB: TRIM63
GST-pull down
ERK1
GST-TRIM63
His-IRF8
GST pd
WB: TRIM63 pS69
ERK1
GST
His
WB: GST
ERK1
GST
His
WB: His
ERK1
GST
His
WB: ERK1

4D

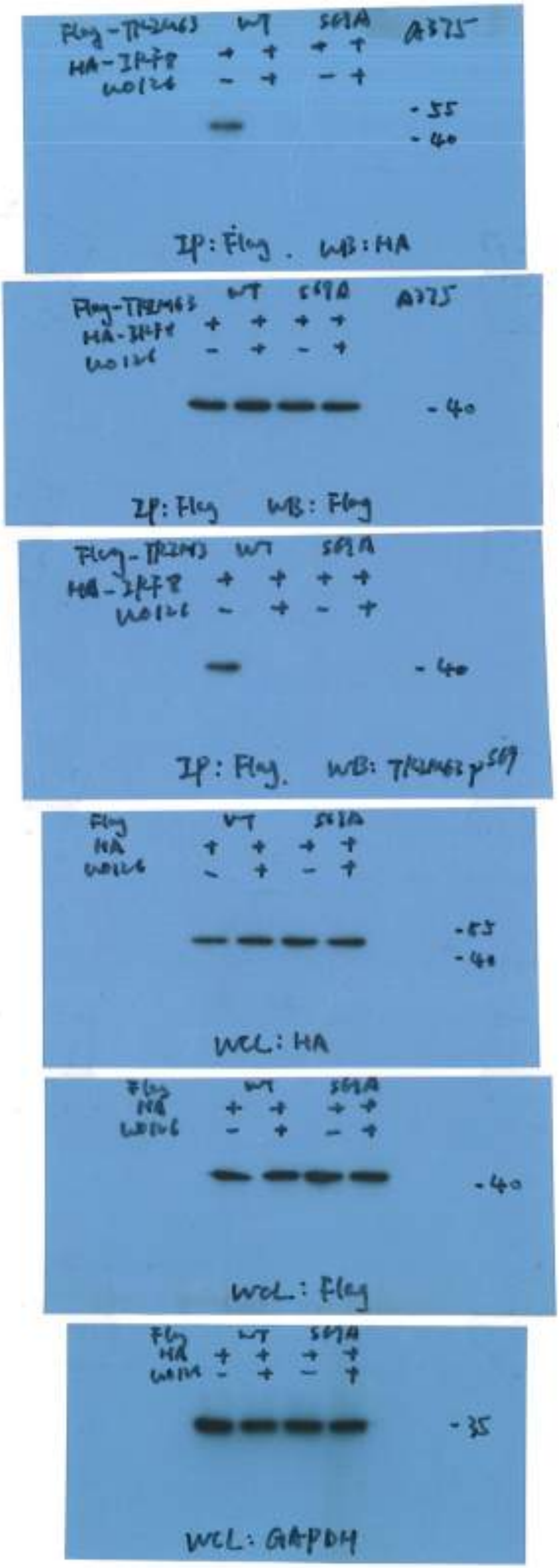
A375
- 55
- 40
IP: Flag. WB: HA
IP: Flag WB: Flag
IP: Flag. WB: TRIM63 p
WCL: HA
WCL: Flag
- 35
WCL: GAPDH

Fig.4E

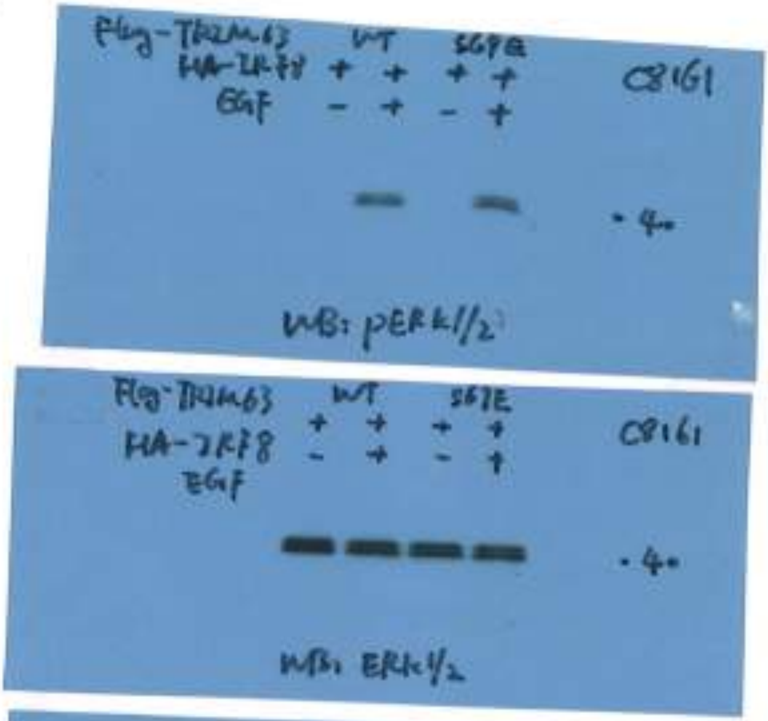
Flag-TRIM63 WT S67E
HA-ZKF8 + + + +
EGF - + - +
C8161
-40
WB: pERK1/2
Flag-TRIM63 WT S67E
HA-ZKF8 + + + +
EGF - + - +
C8161
-40
WB: ERK1/2

Fig 4D

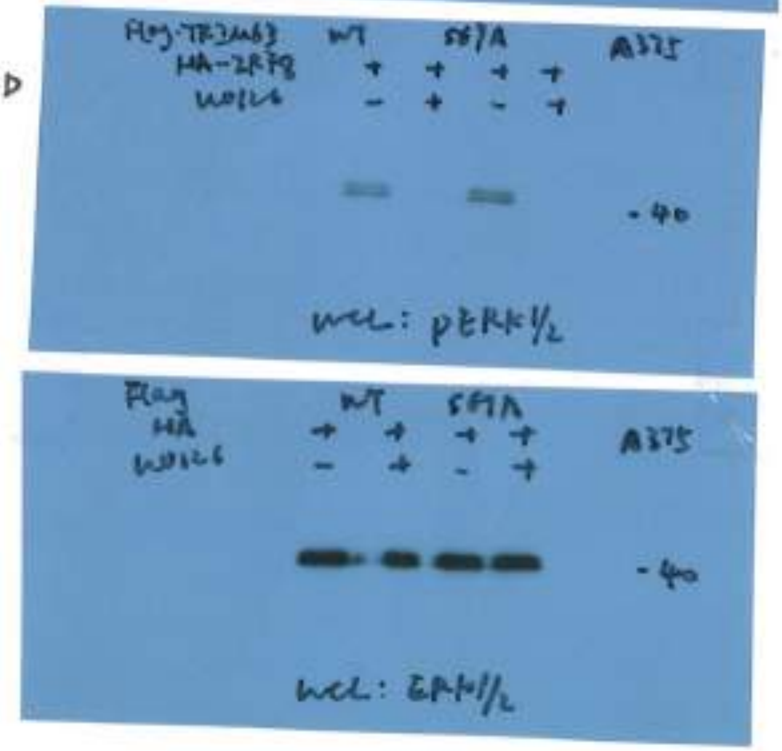
Flag-TRIM63 WT S67A
HA-ZKF8 + + + +
- + - +
A375
-40
WB: pERK1/2
Flag WT S67A
HA + + + +
- + - +
A375
-40
WB: ERK1/2

4E

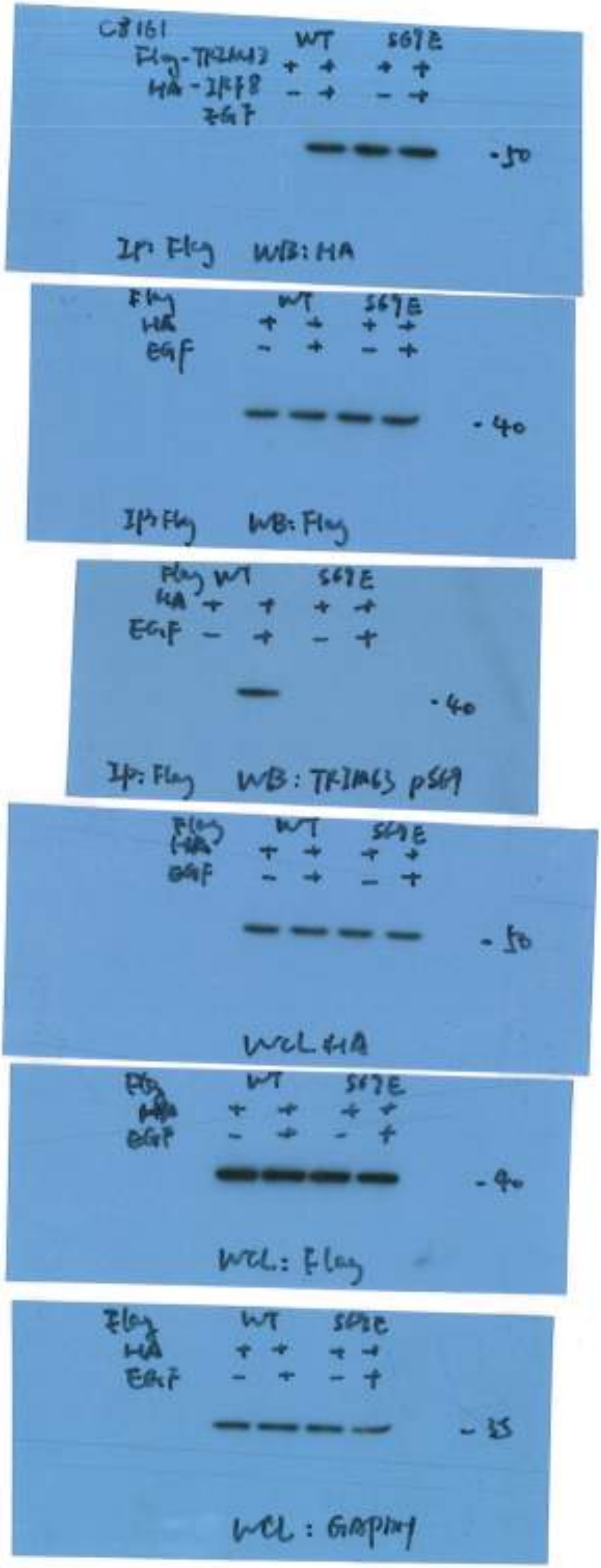

4F

Fig.4F

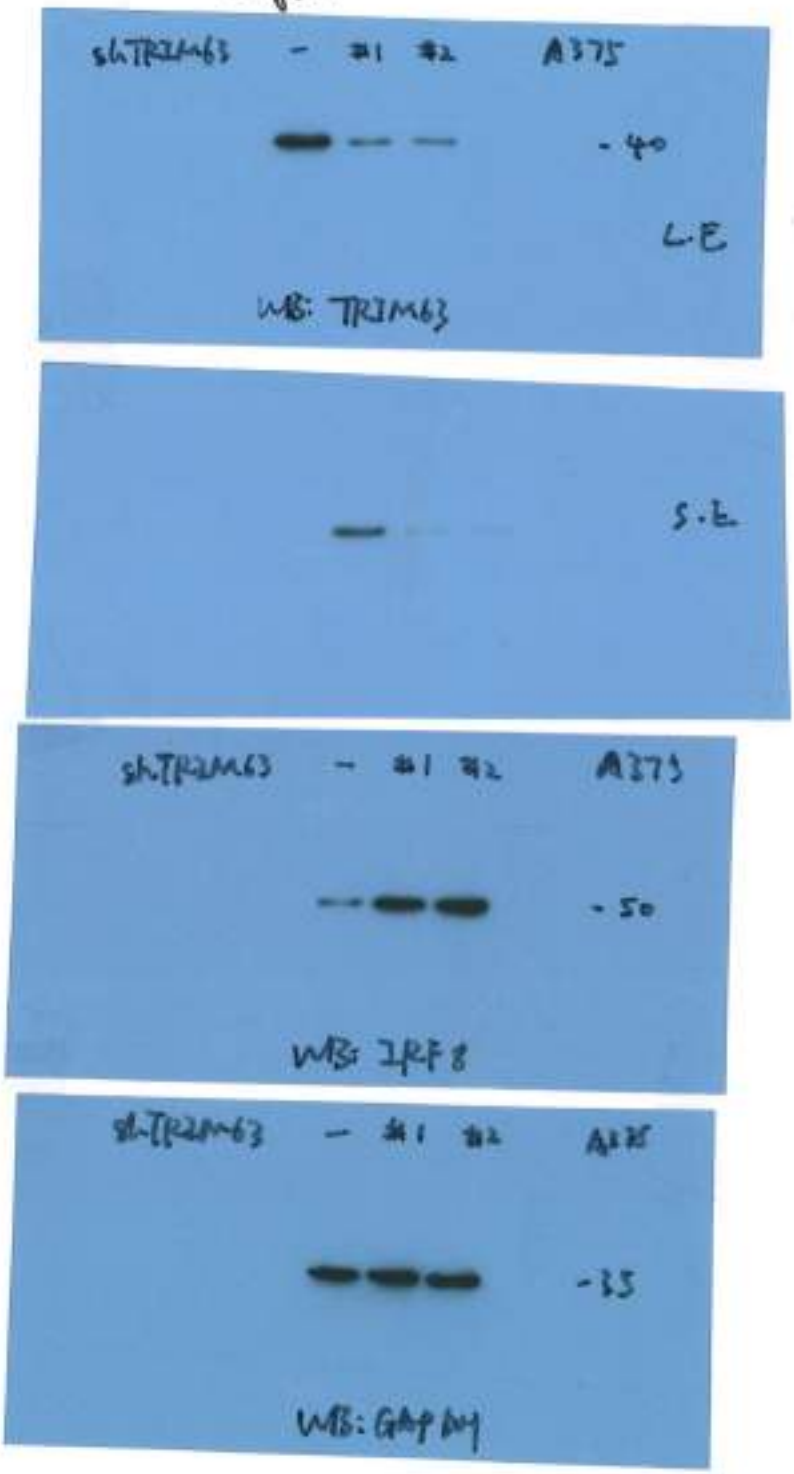
shTRIM63 – #1 #2 A375
-40
L.E
WB: TRIM63
S.E
shTRIM63 – #1 #2 A375
-50
WB: IRF8
shTRIM63 – #1 #2 A375
-35
WB: GAPDH

47

Fig.4F

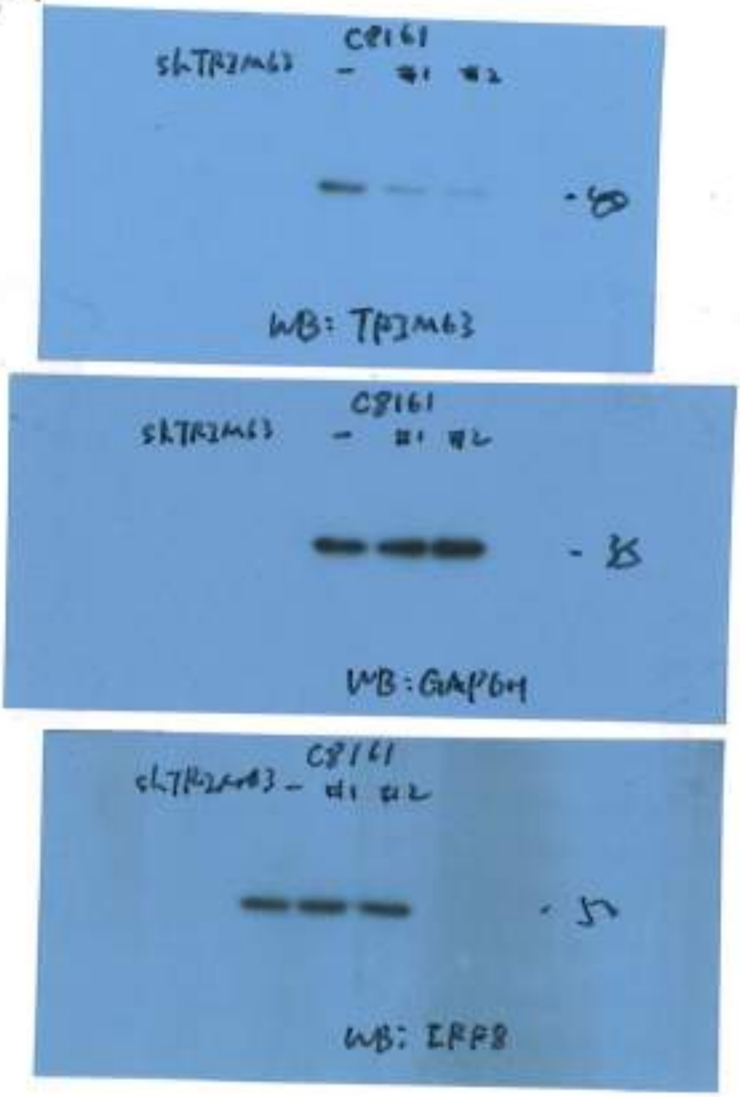
C8161
shTRIM63 - #1 #2
-60
WB: TRIM63
C8161
shTRIM63 - #1 #2
- 35
WB: GAPDH
C8161
shTRIM63 - #1 #2
- 55
WB: IRF8

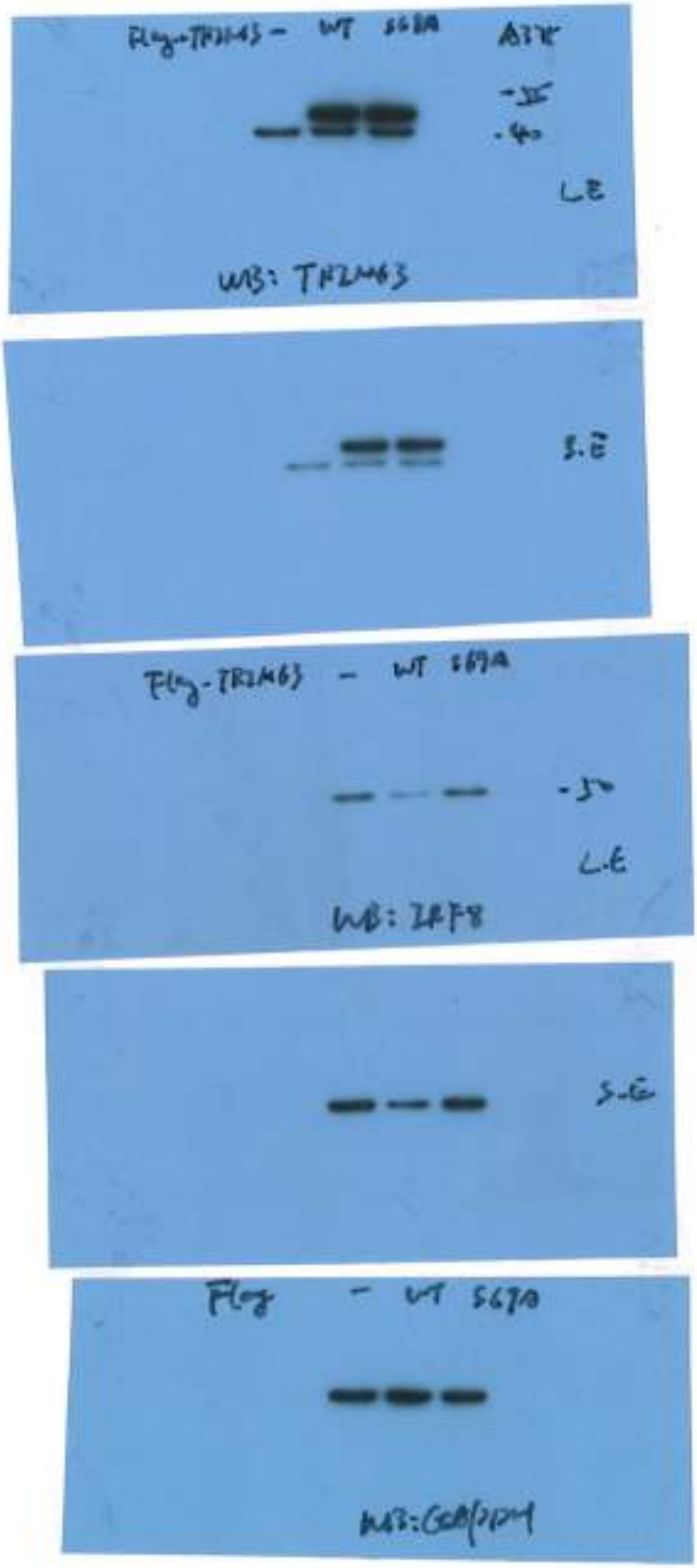
WT
-55
-40
L.E
WB: TRIM63
S.E
WT
-50
L.E
WB: IRF8
S-E
Flag
- WT

4G

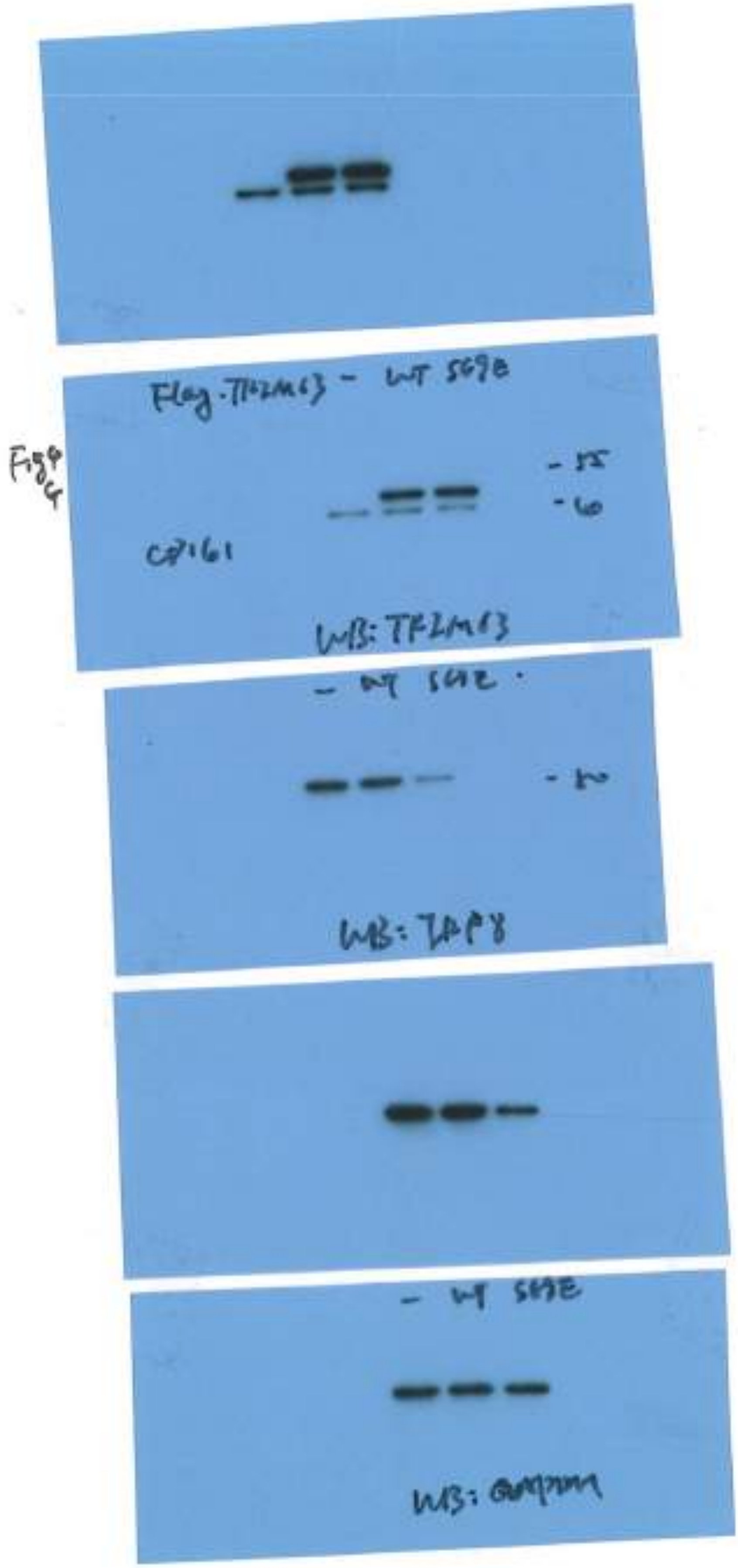

4H

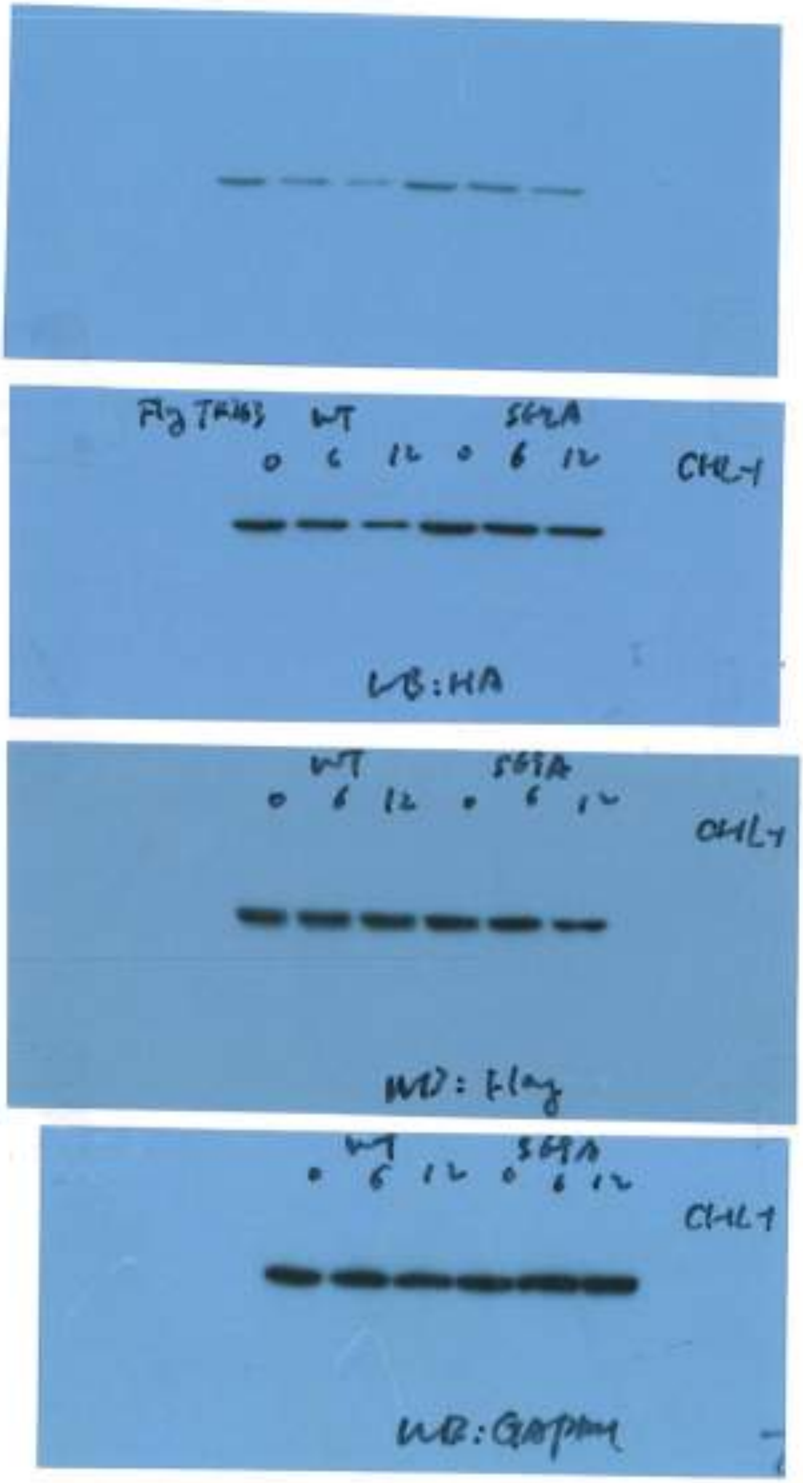

Fig 4H

Fig.44

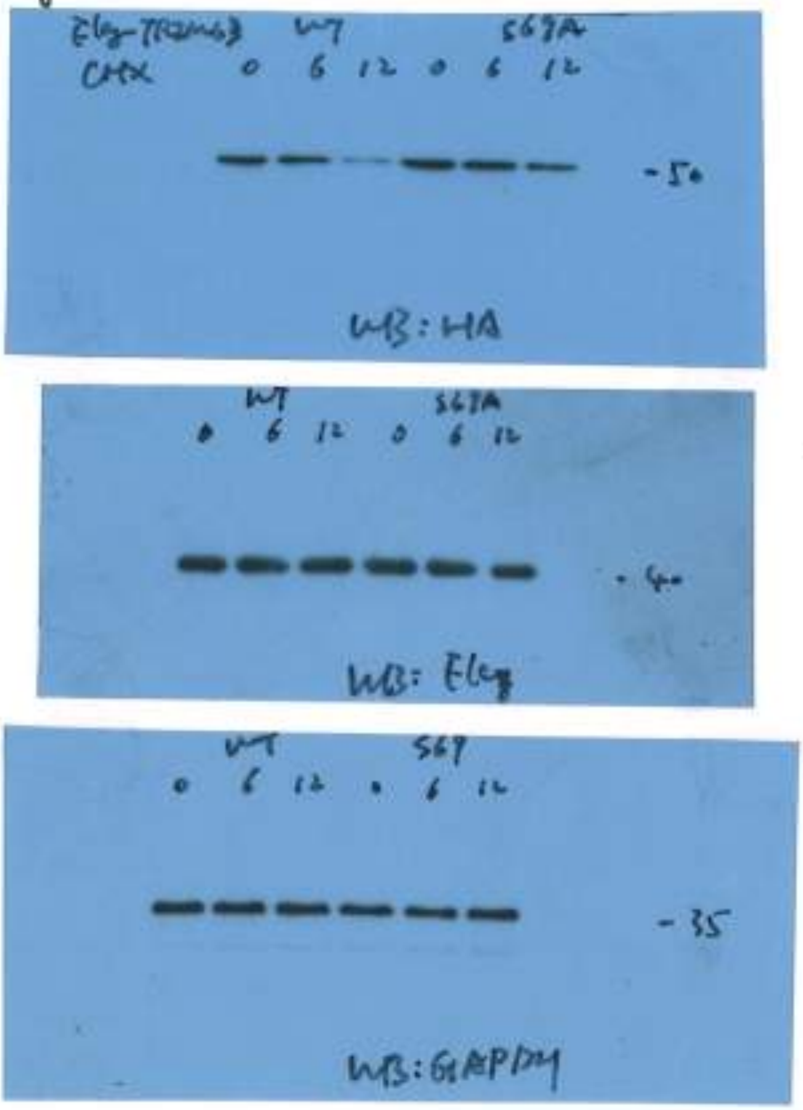

Fig4 I

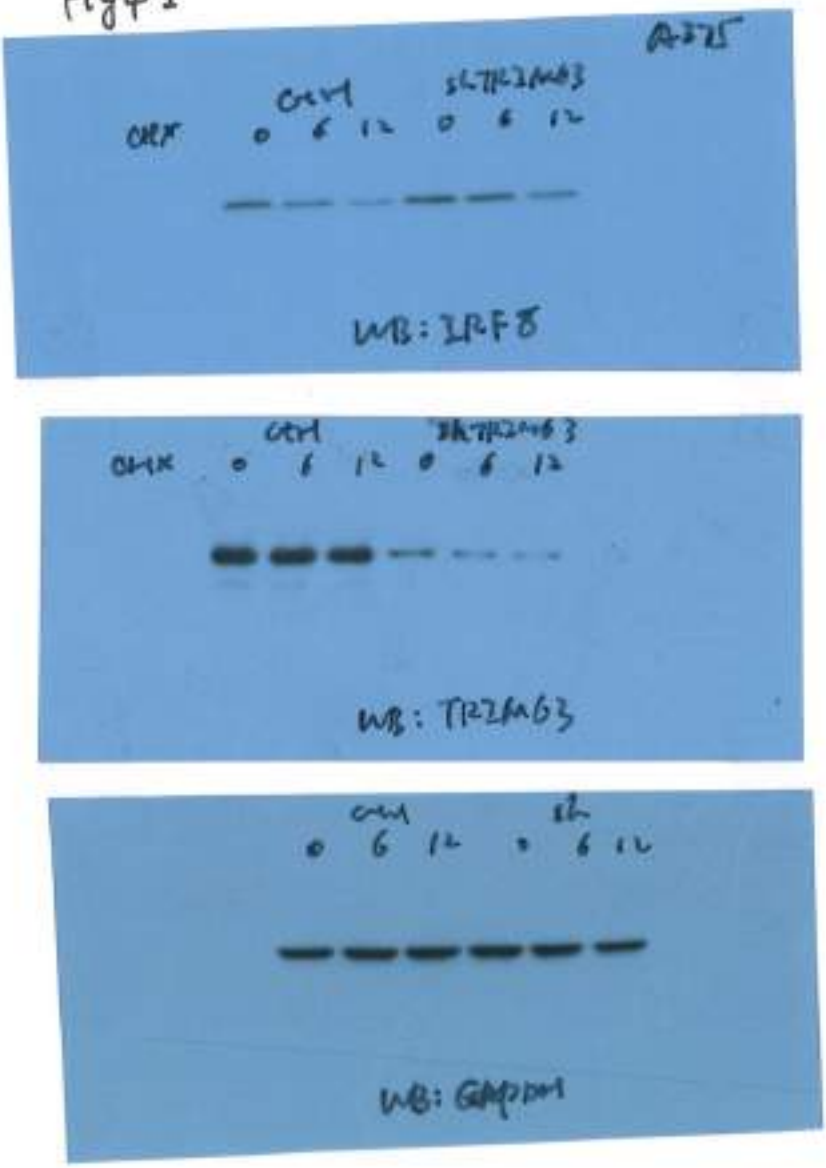
A375
Ctrl
CHX 0 6 12 0 6 12
WB: IRF8
Ctrl
CHX 0 6 12 0 6 12
Ctrl
0 6 12 0 6 12
WB: GAPDH

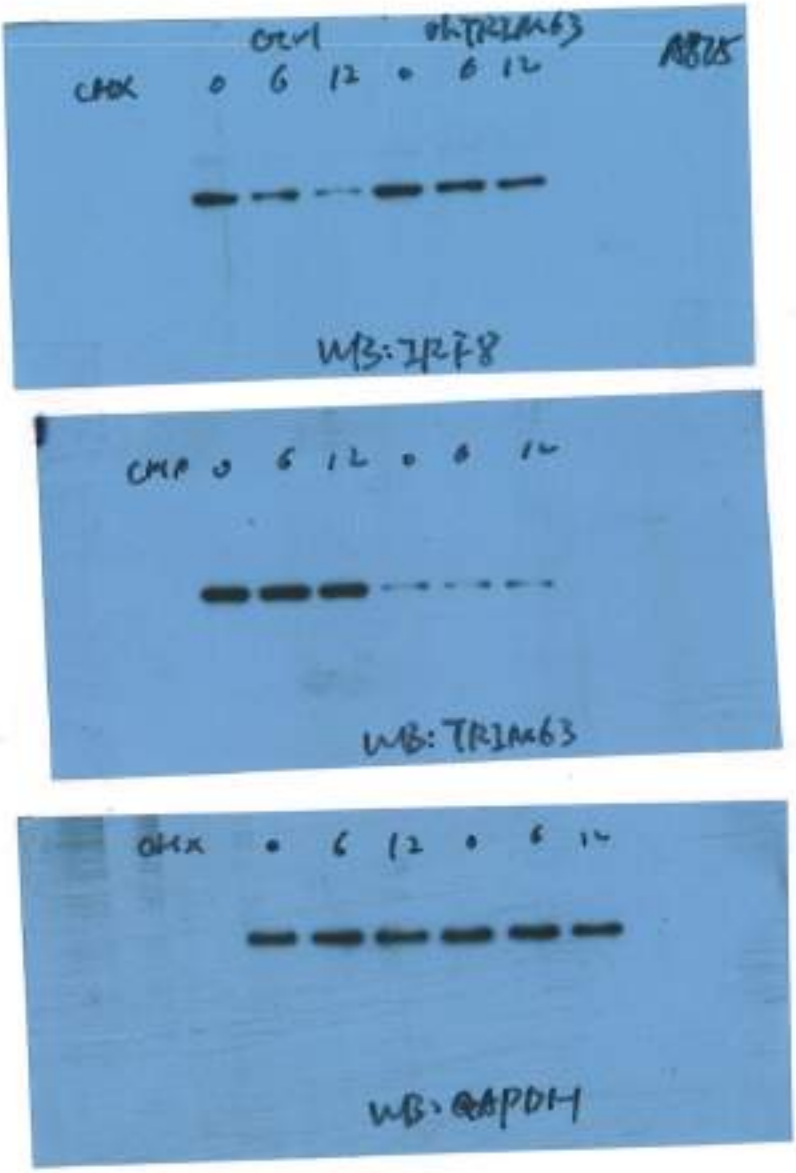
Ctrl shTRIM63
CHX 0 6 12 0 6 12
WB: IRF8
WB: TRIM63
CHX 0 6 12 0 6 12
WB: GAPDH

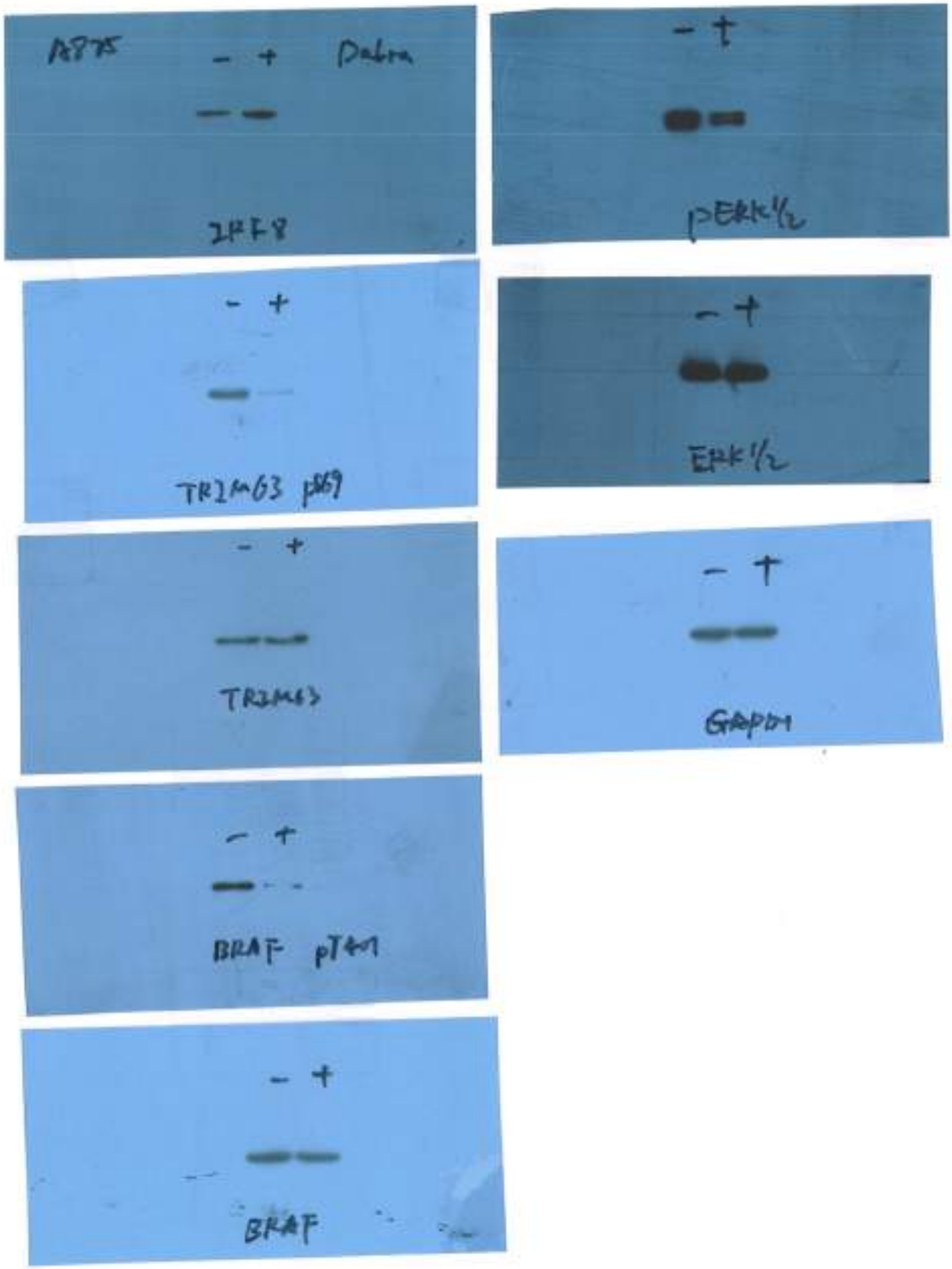
A375
Dabra
IRF8
pERK1/2
TRIM63 pS69
ERK1/2
TRIM63
GAPDH
BRAF pT401
BRAF

4J

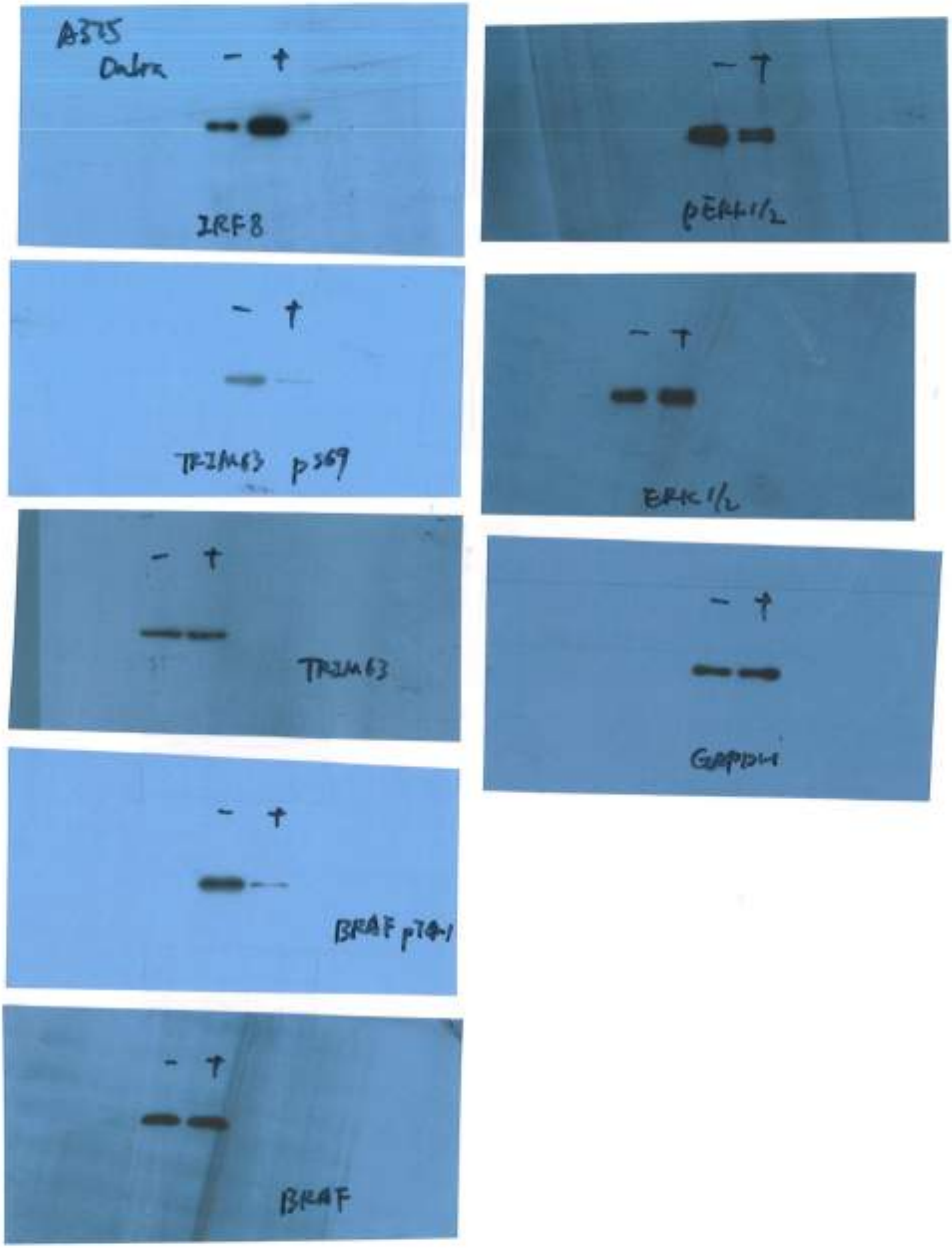

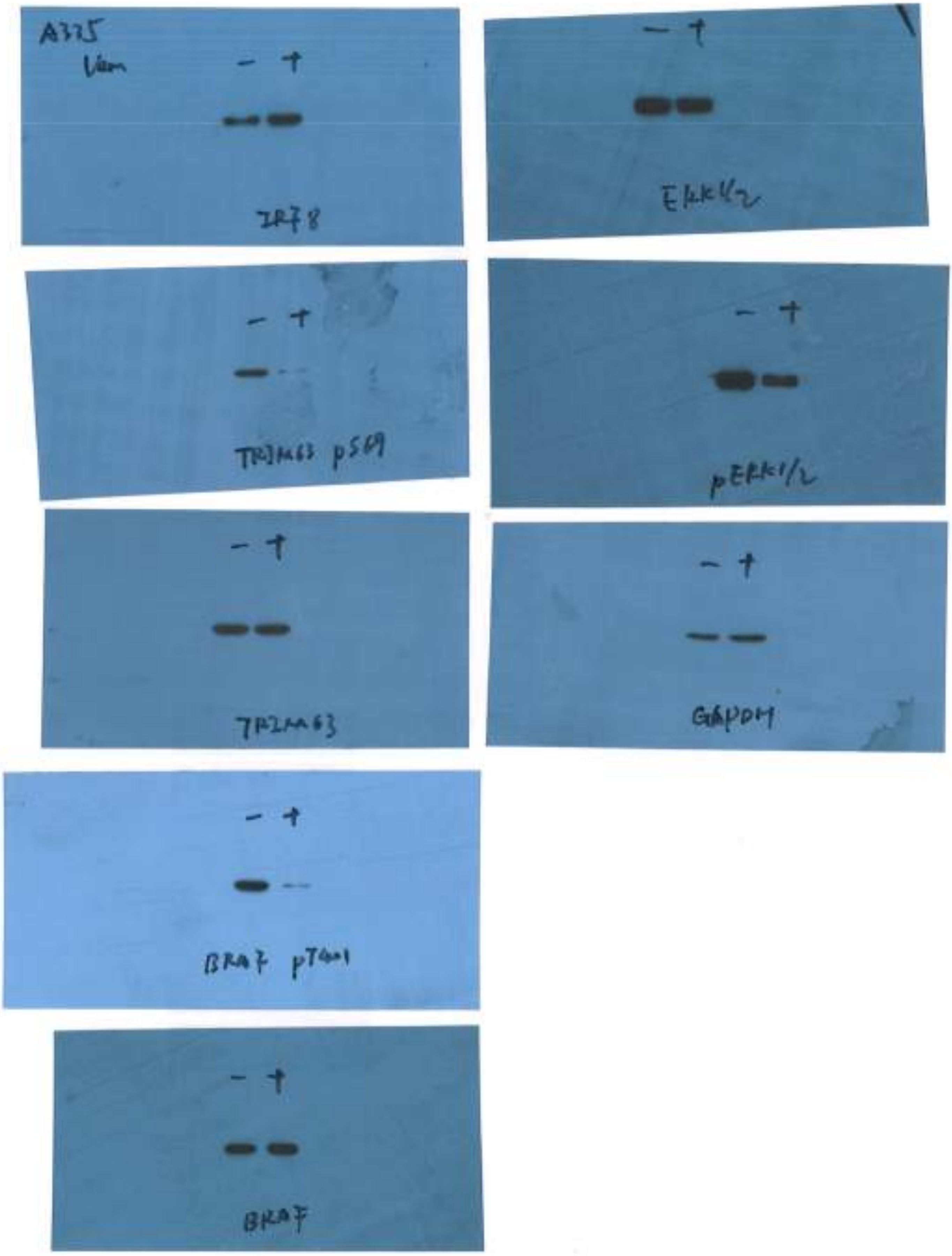
A375
Vem
ERK1/2
pERK1/2
GAPDH
BRAF

HK

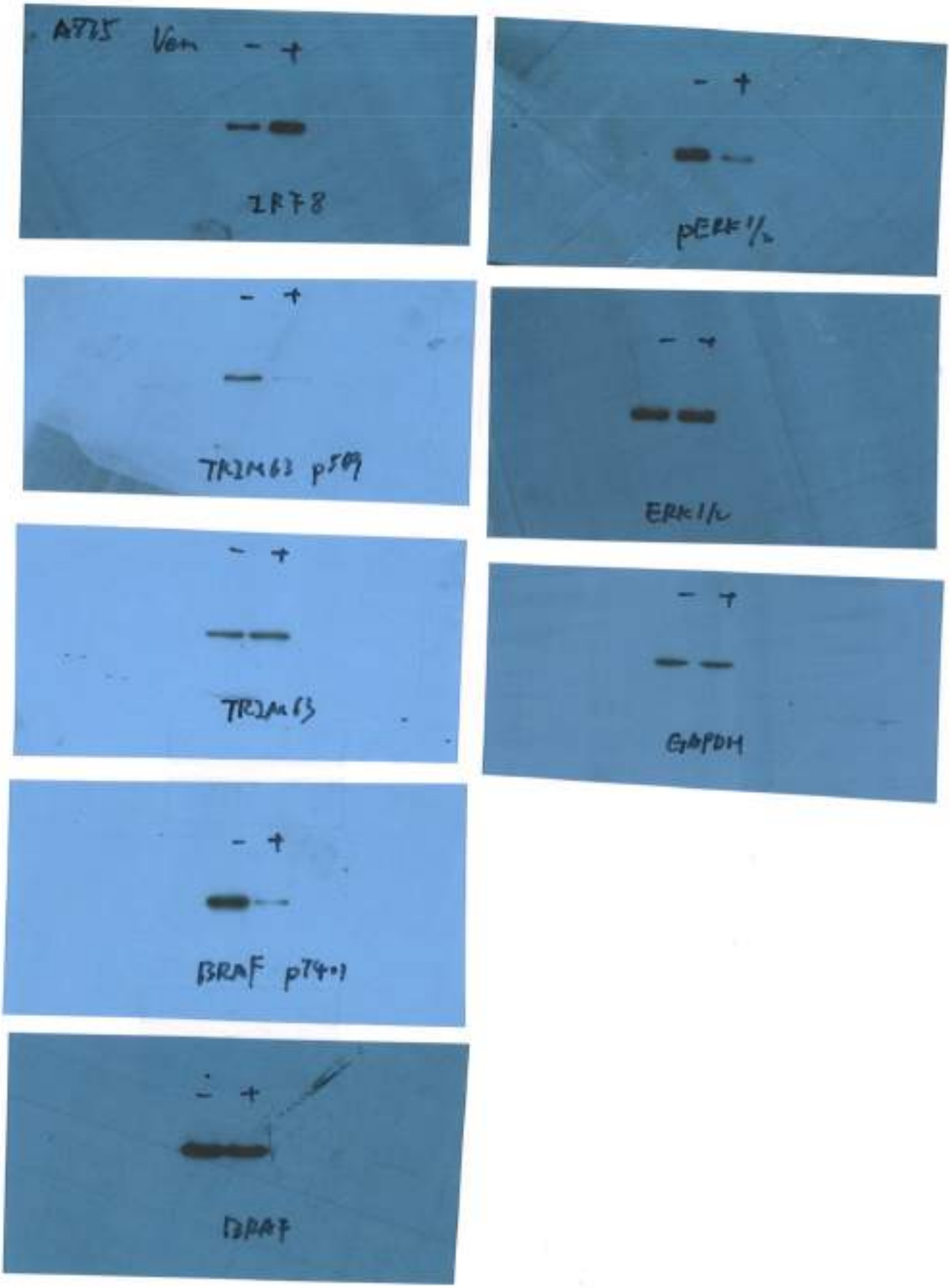

6C

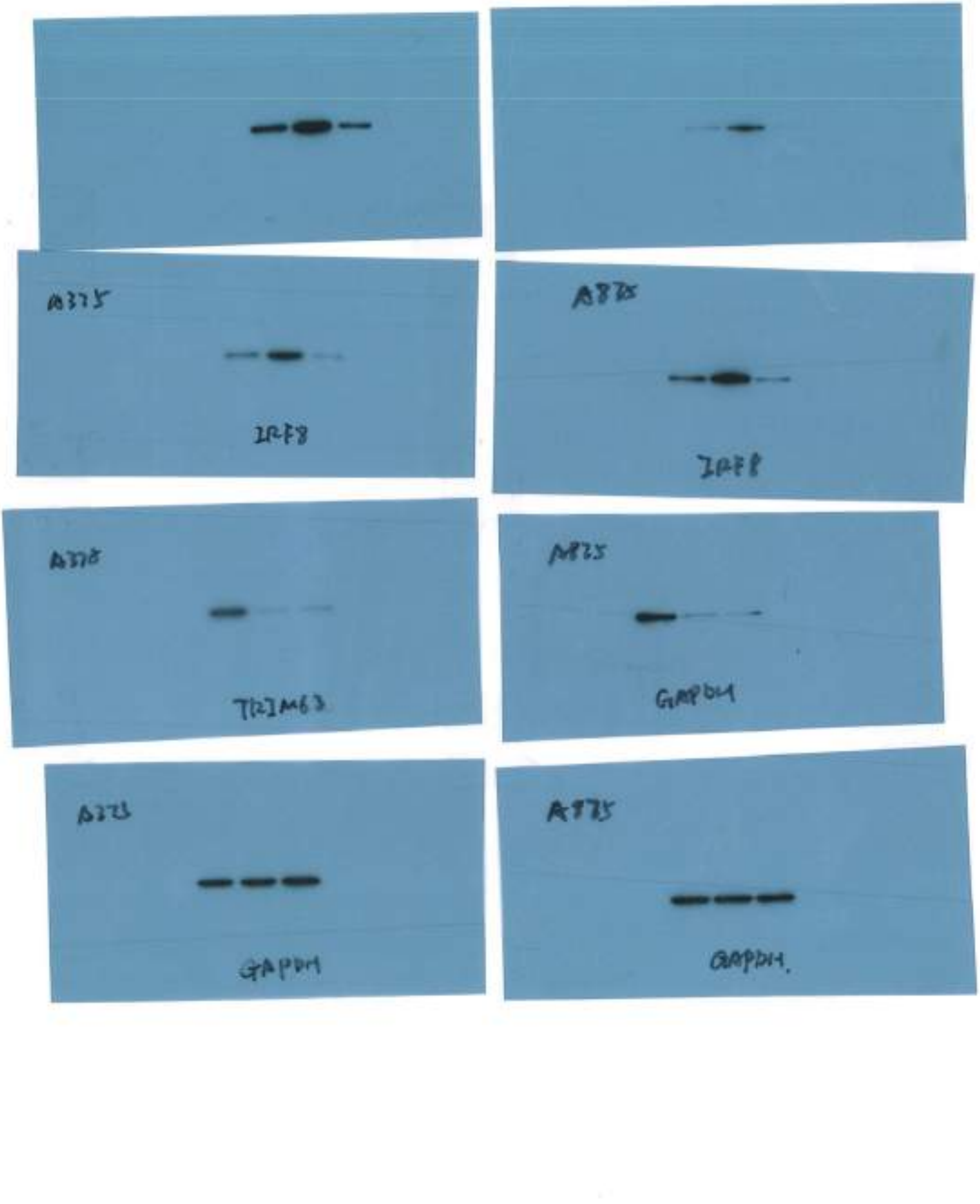
A375
IRF8
A875
IRF8
A375
TRIM63
A875
GAPDH
A375
GAPDH
A875
GAPDH.

6A

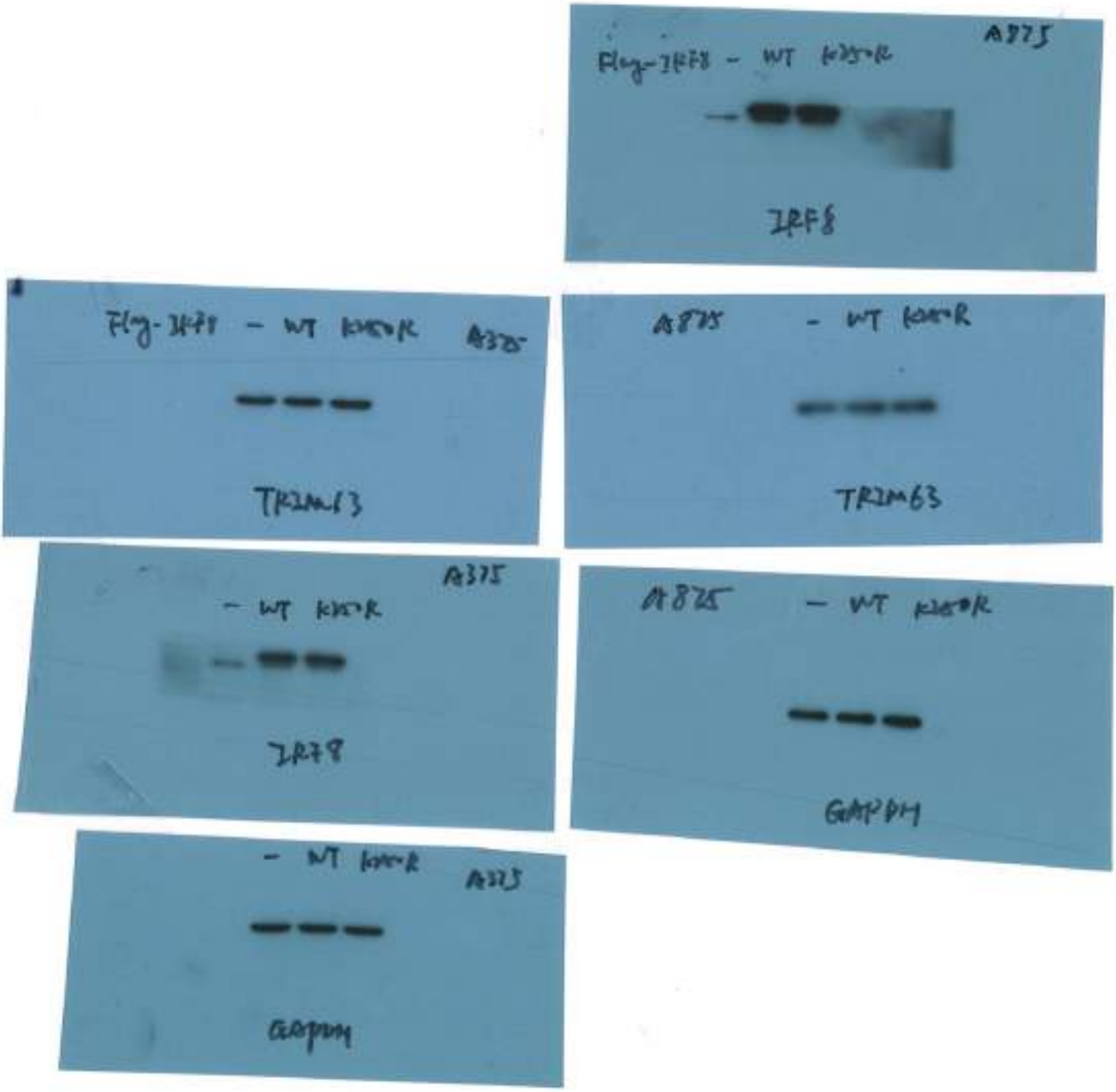

Supplement: Supplementary file 5 — Raw data (wb) [file 41419_2025_8216_MOESM5_ESM.pdf]
